# Core-Labeling (Radio) Synthesis of Phenols

**DOI:** 10.1021/acs.orglett.3c02838

**Published:** 2023-09-26

**Authors:** Colin F. Lynch, Joseph W. Downey, Yongliang Zhang, Jacob M. Hooker, Mark D. Levin

**Affiliations:** † Department of Chemistry, University of Chicago, Chicago, Illinois 60637, United States; ‡ Athinoula A. Martinos Center for Biomedical Imaging, Massachusetts General Hospital, Charlestown, Massachusetts 02129, United States; § Department of Radiology, Harvard Medical School, Boston, Massachusetts 02115, United States; ∥ Lurie Center for Autism, Massachusetts General Hospital, Lexington, Massachusetts 02421, United States

## Abstract

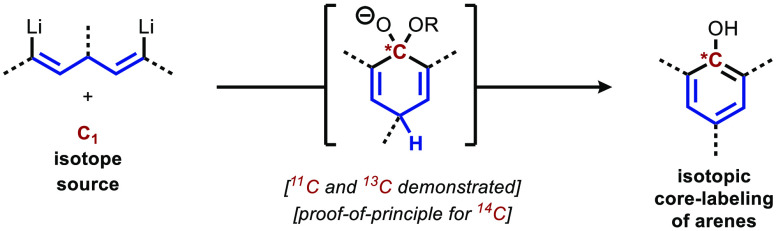

We report a method that enables the fast incorporation of carbon isotopes into the *ipso* carbon of phenols. Our approach relies on the synthesis of a 1,5-dibromo-1,4-pentadiene precursor, which upon lithium–halogen exchange followed by treatment with carbonate esters results in a formal [5 + 1] cyclization to form the phenol product. Using this strategy, we have prepared 12 1-^13^C-labeled phenols, show proof-of-concept for the labeling of phenols with carbon-14, and demonstrate phenol synthesis directly from cyclotron-produced [^11^C]CO_2_.

Site-specific carbon-isotope labeling of small molecules has wide ranging applications across fundamental biology, drug and agrochemical development, and medical imaging, with each isotope suited for different applications (Figure 1a).^1^ Radioligands labeled with the short-lived carbon-11 isotope can be tracked using positron emission tomography (PET), a powerful and quantitative imaging modality for probing molecular interactions *in vivo*.^2^ The carbon-13 isotope has found far-reaching utility in the labeling of mass spectrometry internal standards and NMR spectroscopy probes in many fields. Additionally, carbon-13-labeled molecules are gaining utility in the emerging molecular imaging technique of hyperpolarized magnetic resonance imaging.^3^ Meanwhile, radioactive carbon-14-labeled compounds have long been considered the gold standard for adsorption–distribution–metabolism–excretion (ADME) studies, a vital development stage for determining the safety and efficacy of drug and agrochemical candidates.^4^ Though incorporation of each isotope presents very different logistic challenges (*e.g*., carbon-13 is stable whereas carbon-11 has a half-life of 20 min), they share a similar need for synthetic approaches that incorporate readily available single-carbon synthons into molecular scaffolds.

**Figure 1:**
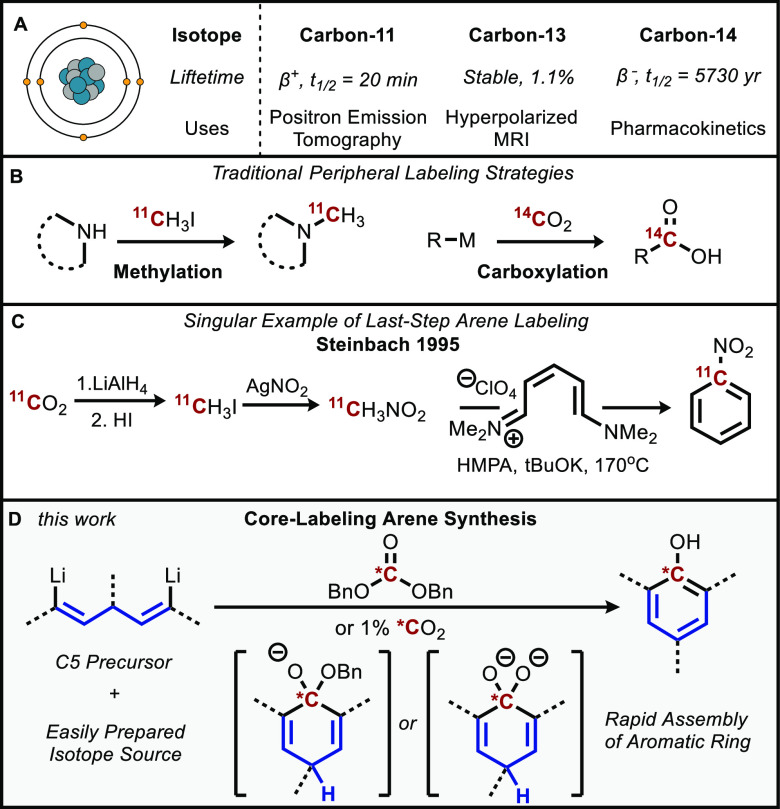
Introduction. (A) Properties and uses for carbon isotopes. (B) Common strategies for peripheral carbon isotope labeling. (C) Prior art in last-step arene labeling. (D) This work, core labeling of phenols.

While there are many reported methods for the efficient incorporation of carbon isotopes into small molecules, most of these methods install the desired isotope on the periphery of the molecule (Figure 1b). For example, the most common approach for carbon-11 radiolabeling is methylation of pendant alcohols or amines with [^11^C]CH_3_I or [^11^C]CH_3_OTf.^5^ Similarly, carbon-14 is frequently incorporated into molecules via carboxylation, traditionally from direct fixation of organometallic precursors with [^14^C]CO_2_,^6^ though recent advances in isotopic exchange of carboxylic acids have also been reported.^7−10^ These strategies in turn make the isotopic label more prone to metabolic cleavage; while this can have some advantages when detectable radiometabolites are unwanted, it can also be a hindrance, either for the timing of imaging studies with radiotracers or when metabolite visibility is desirable, such as in pharmacokinetic assays.^11^ Particularly for carbon-14 ADME studies, placement of the radioactive label dictates which radioactive metabolites are detected, with peripheral incorporation limiting the observable downstream metabolites.^12^ Moreover, not all molecules of interest possess such peripheral functional handles, limiting the number of molecules that can be prepared via late-stage isotopic labeling. Even those that are amenable to peripheral incorporation would stand to benefit from the accessibility of additional isotopomers.

Thus, incorporation of carbon isotopes into the core position of molecules remains an important challenge.^13^ While per-^13^C-labeled arenes are commercially available (e.g., [^13^C_6_]benzene), and some reported methods offer access to bislabeled arenes, monolabeled compounds are far rarer and have complementary applications. The majority of existing approaches to monolabeled arenes incorporate the isotope early and involve multiple subsequent manipulations.^14−18^ As the isotopic label is often the most valuable component, it is preferable to install it as late as possible to limit the depletion of the isotope over iterative yield losses. This is especially true for the short-lived carbon-11 isotope, where subsequent manipulations are limited by decay.^19,20^ In this vein, Steinbach reported a singular example of the preparation of [1-^11^C]nitrobenzene and aniline by the reaction of [^11^C]nitromethane (produced in three steps from [^11^C]CO_2_) with a pentamethinium salt and KO^*t*^Bu in HMPA solvent at 170 °C (Figure 1c).^21^ These reports underscore the necessity for further developments in this space.

We report here a method that allows for a core-labeling synthesis of phenolic compounds with an isotopic incorporation in the final step. The protocol detailed below enables the incorporation of carbon isotopes into the aromatic *ipso* carbon of phenols (Figure 1d).

We were inspired by a recent report by Sparr and co-workers, who found that a 1,5-dimagnesiopentadiene reagent could undergo double addition to esters, with 1,4-elimination upon acidic workup affording a new aromatic ring.^22^ We envisioned that a similar 1,5-diorganometallic reagent could react with simple 1-carbon electrophiles at the formal +4 oxidation state to form a new isotopically labeled phenol. Sparr’s precursor synthesis, however, was amenable only to the unsubstituted parent reagent, which in this instance would yield an unsubstituted phenol, necessitating the development of an alternative protocol. A representative synthesis of the necessary 1,5-dibromo precursor is shown in Figure 2a. We began from the 1,4-dialkyne alcohol (**1a**) which was prepared in one step from the overaddition of a lithium acetylide onto a formate ester (or acid anhydride for *para*-substituted phenols). Next, double *trans*-hydroalumination using sodium bis(2-methoxyethoxy) aluminum hydride (Red-Al) followed by quenching with *N*-bromosuccinimide formed the dibromide alcohol **2a** with the requisite *anti*-stereoselectivity. Such hydroaluminations are well studied for propargylic alcohols, but to our knowledge the simultaneous reduction of two alkynes in bis-propargylic alcohols is unprecedented.^23^ Finally, a hydrosilane reduction activated by trifluoroacetic acid resulted in the final vinylic 1,5-dibromo precursor **3a**. Full details regarding syntheses for the remaining dibromo precursors employed below can be found in the Supporting Information.

**Figure 2:**
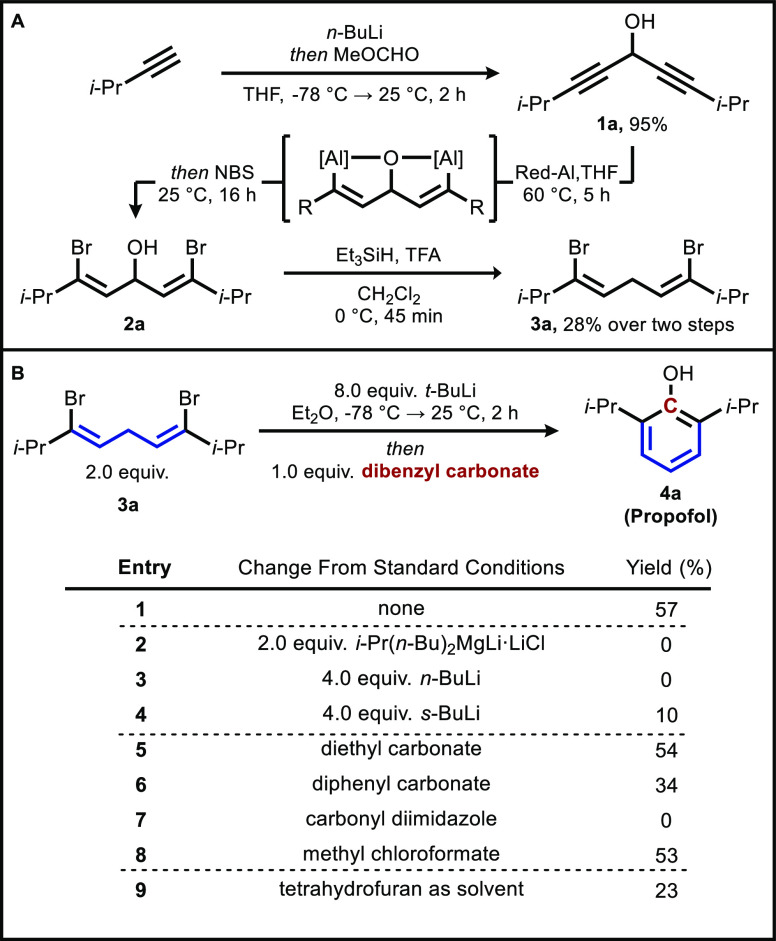
(A) Representative synthesis of the 1,5-dibromide precursor and (B) optimization of phenol cyclization. Reactions were performed under nitrogen on a 0.04 mmol scale, and yields were determined by ^1^H NMR using mesitylene as an internal standard.

With the precursor in hand, we then screened different conditions for metal–halogen exchange^24^ and subsequent cyclization with limiting one-carbon electrophiles to form our model substrate 2,6-diisopropylphenol (propofol, **4a**, Figure 2b). Our optimized conditions use 8 equiv of *tert*-butyllithium (four relative to the dibromide precursor, or two per bromide, as is typical for *t*-BuLi halogen exchange) to produce a dilithiate intermediate, followed by treatment with dibenzyl carbonate to form the desired phenol.

Commonly used bromine–magnesium exchange reagents (*e.g.*, *i*-PrMgCl·LiCl and *i*-Pr(*n*-Bu)_2_MgLi)^25^ and *n*-butyllithium did not result in effective metalation and afforded no phenol product. The stronger *sec*-butyllithium could form the necessary intermediate; however, *tert*-butyllithium was found to be far superior. Of the electrophiles tested, alkyl carbonate esters and chloroformates proved to be the most effective, with an aryl carbonate and carbonyl diimidazole giving much lower yields. Interestingly, the reaction proceeds with the highest yields in diethyl ether, and solvents that coordinate more strongly with lithiates (*e.g*., tetrahydrofuran) give lower yields of phenol.

Ultimately, the stability of carbonate esters compared to chloroformates led us to choose dibenzyl carbonate (**5**) as our optimized carbon isotope source, and we used carbon-13 to test our isotopic labeling method on a preparative scale. First, we prepared **[carbonyl**-**^13^C]5** from benzyl chloride and potassium carbonate, an economic source of carbon-13, on 5 mmol scale in 72% yield.^26^ Our optimized synthesis relies on the combination of two phase transfer catalysts (18-crown-6 and Aliquat-336) to afford the product rapidly and reproducibly.

Delightfully, we found that the yield of our model substrate, propofol (**[1-**^**13**^**C]**4a****), improved to 79% on a larger scale, and in total we have prepared 12 different carbon-13-labeled phenol products (Figure 3). 2,6-Diphenyl phenol ([**1-**^**13**^**C]**4e****) was produced in a high yield, and two unsymmetric phenols featuring aromatic and alkyl ethers (**[1-**^**13**^**C]**4g**** and **[1-**^**13**^**C**]**4h**) were also isolated. Additionally, an *ortho*-unsubstituted example (4-phenyl phenol, **[1-**^**13**^**C]**4f****) was well tolerated under the reaction conditions and produced in a 74% yield. A series of *para*-substituted 2,6-diphenylphenols (**[1-**^**13**^**C]**4i**** through **[1-**^**13**^**C]**4l****) were also prepared in good yields. Naturally, functional groups incompatible with organolithium reagents (acids and electrophiles) were not tolerated. In all cases, ^13^C-enrichment (as measured by quantitative ^13^C NMR) was greater than 97%.

**Figure 3:**
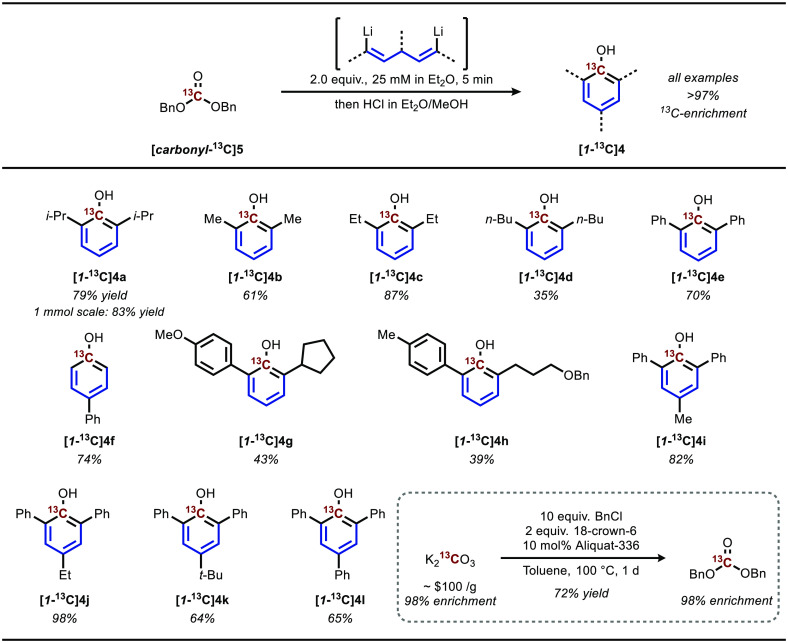
Synthesis of [*carbonyl*-^13^C]dibenzyl carbonate and scope of 1-^13^C phenols. Isolated yields on 0.20 mmol scale.

This series of labeled phenols could be particularly useful as hyperpolarized MRI probes. An effective HP-MRI probe should have a carbon-13 enriched site with a long longitudinal relaxation time (*T*_1_) to preserve the MR signal and provide more accurate quantification. The 1-^13^C label of these phenols is ideal, as the carbon-13 isotope lacks directly attached hydrogen atoms that could shorten its *T*_1_ time.^3,27^ The *T*_1_ of [**1-**^**13**^**C]**4a**** was measured to be 29.4 s at 11.7 T, which is similar to other studied HP-MRI probes.

The success of our results with carbon-13 led us to pursue the labeling of phenols from readily available sources of carbon-14 (Figure 4a). Since carbon-14 decays very slowly, we first sought to synthesize dibenzyl carbonate from commercially available sources of carbon-14. Sodium carbonate can also be used to produce dibenzyl carbonate in a similar manner as that described for potassium carbonate; however, use of *N,N*-dimethylformamide as a solvent and the addition of cesium chloride were found to perform much better than our parent conditions due to the differing solubility properties of the sodium salt. While [^14^C]Na_2_CO_3_ is commercially available, it is significantly more expensive than bulk [^14^C]BaCO_3_. Indeed, [^14^C]BaCO_3_ is the universal starting material for carbon-14-labeled compounds, from which [^14^C]CO_2_ can be released upon acidolysis.^6,28^ Thus, we additionally demonstrated the synthesis of dibenzyl carbonate from barium carbonate in a COware two-chamber setup,^29^ modifying conditions from Jang and co-workers.^30^

**Figure 4:**
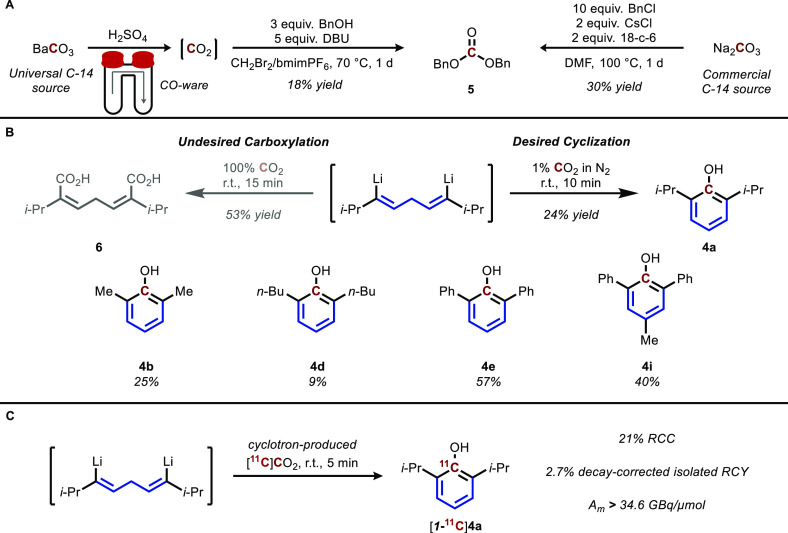
Demonstration of feasibility for carbon radioisotopes. (A) Synthesis of dibenzyl carbonate from BaCO_3_ and Na_2_CO_3_, common carbon-14 sources. (B) Synthesis of phenols directly from dilute CO_2_, a model system for carbon-11. (C) Radiosynthesis of [1-^11^C]propofol from cyclotron-produced [^11^C]CO_2_.

Contrary to the other isotopes, carbon-11 has an exceptionally short half-life of approximately 20 min, making direct use of cyclotron-produced [^11^C]CO_2_ advantageous to preserve radiochemical yield. Fixation of carbon-11 with organometallic precursors has been well precedented,^31^ and methods for direct incorporation of [^11^C]CO_2_ have seen a recent renewed interest.^32−35^ Thus, we were interested in producing phenols directly from CO_2_ as the source of *ipso* carbon. Initial experiments treating the reactive propofol dilithiate precursor directly with an atmosphere of CO_2_ gas resulted in simple double carboxylation to produce the undesired acid **6**. However, decreasing the concentration in the gaseous stream to 1% CO_2_ resulted in the desired cyclization and propofol product in a modest yield of 24% (Figure 4b). These conditions were repeated with a small group of precursors, with results following similar trends in yield seen for the carbonate protocol. Since this phenol synthesis occurs in <10 min and works well with low concentrations of CO_2_, it is particularly well suited to the low-nanomole scale of carbon-11 radiosynthesis.^36^

Indeed, the propofol dilithiate precursor was able to react with cyclotron-produced [^11^C]CO_2_ to form [1-^11^C]propofol ([1-^11^C]**4a**) with a moderate radiochemical conversion of 21% (31% relative to 69% trapping efficiency), and the decay-corrected radiochemical yield for [1-^11^C]propofol after isolation was 2.7% (Figure 4c).^37^ Despite the low isolated RCY, this direct incorporation of [^11^C]CO_2_—in comparison with ^11^C-methylation with [^11^C]CH_3_I—provides more rapid access to radiolabeled products (isolation within 27 min from end of bombardment). Thus, at typical clinical [^11^C]CO_2_ production scales (approximately 1.5–2 Ci), this reaction should provide sufficient quantities of radiotracer for imaging studies. Due to the low UV absorbance of propofol, the molar activity of [1-^11^C]propofol could not be determined; however, a lower bound of 34.6 GBq/μmol at end of synthesis could be determined, roughly in line with the 37 GBq/μmol desired range for a PET radiotracer.^38^

## Data Availability

The data underlying this study are available in the published article and its Supporting Information.

## Supporting Information Available

Experimental procedures, characterization data and spectra (PDF)
