# Supplementary material for: Core-Labeling (Radio) Synthesis of Phenols
Source: Org Lett. 2023 Sep 26;25(39):7230–5. doi: 10.1021/acs.orglett.3c02838 (PMC10563162; doi:10.1021/acs.orglett.3c02838)

Core-Labeling (Radio)Synthesis of Phenols

Supporting Information

Authors: Colin F. Lynch,[1] Joseph W. Downey,[2] Yongliang Zhang,[1]
Jacob M. Hooker,[2,*] Mark D. Levin[1,*]

[1]Department of Chemistry, The University of Chicago, 5735 S. Ellis Ave., Chicago, IL 60637

[2]Athinoula A. Martinos Center for Biomedical Imaging, Massachusetts General Hospital, Charlestown, Massachusetts 02129, United States

*Email: marklevin@uchicago.edu and jhooker@mgh.harvard.edu

Table of Contents

General Considerations 2

Synthesis of Phenols 3

Optimization of Reaction Conditions 12

Carbon-11 Radiochemistry 15

Synthesis of Dibenzyl Carbonate from Carbonate Salts 20

Synthesis of Dibromide Precursors 22

Synthesis of Dialkyne Alcohols 29

Carbon-13 T_1 Measurement 35

References 37

NMR Spectra of New Compounds 38

General Considerations

Unless otherwise stated, all reactions were performed under a dry nitrogen atmosphere in oven-dried glassware equipped with a magnetic stir bar. Where noted, reactions performed in a glovebox were performed under a nitrogen atmosphere in an MBraun UniLab Pro SP system with residual oxygen and water maintained under 2.0 ppm. Diethyl ether (Et_2O), tetrahydrofuran (THF), dichloromethane (CH_2Cl_2), toluene (PhMe), acetonitrile (MeCN), triethylamine (Et_3N), and pentane were dried by passage through a column of activated alumina under an argon atmosphere using a Pure Process Technology solvent purification system. All other organic solvents were dried over activated molecular sieves (4Å) and degassed prior to use. Reaction temperatures are reported relative to the oil bath surrounding the reaction vessel.

Thin-layer chromatography (TLC) was performed on glass-backed plates of 250μm thickness coated with Silica Gel 60 F254 or neutral alumina F254 as noted. Plates were visualized with UV irradiation or staining as noted.

High resolution mass spectra were recorded on either an Agilent 6224 TOF High Resolution Accurate MS with electrospray ionization or an Agilent 7200B QTOF High Resolution Accurate Mass GCMS using an Agilent HP-5MS column with a temperature gradient of 50 °C to 200 °C over 30 minutes and electron ionization. All mass spectra were processed with an Agilent MassHunter Operating System.

Nuclear magnetic resonance spectra (^{1}H NMR, ^{13}C NMR) were recorded with Bruker spectrometers operating at 400 MHz or 500 MHz for ^{1}H. All NMR spectra were processed with Mestrelab Research MestReNova. Chemical shifts are reported in parts per million (ppm, δ), downfield from tetramethylsilane (TMS, δ = 0.00 ppm) and are referenced to residual solvent ($CDCl_3$, δ = 7.26 ppm (^{1}H) and 77.16 ppm (^{13}C)). Coupling constants are reported in Hertz (Hz). Data for ^{1}H-NMR spectra are reported as follows: chemical shift (ppm, s = singlet, d = doublet, t = triplet, q = quartet, quin = quintet, dd = doublet of doublets, td = triplet of doublets, ddd = doublet of doublet of doublets, m = multiplet, coupling constant (Hz), and integration).

Synthesis of Phenols

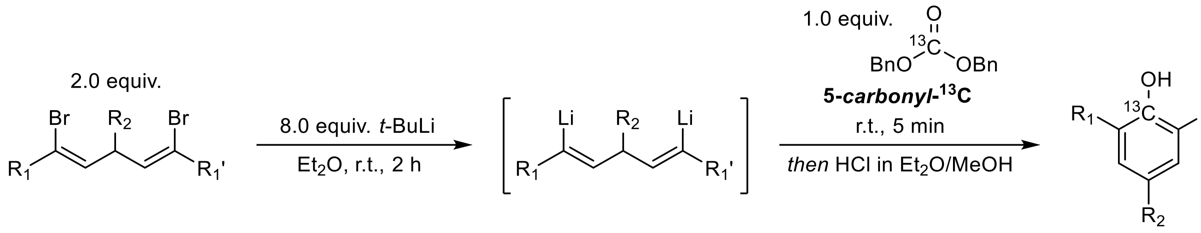

3 **4-*1*-^{13}C**

General procedure A for the synthesis of *1*-^{13}C phenols from dibenzyl carbonate-*carbonyl*-^{13}C

In a glovebox under a nitrogen atmosphere, 0.425 mmol (2.0 equiv.) dibromide precursor **3** was added to a scintillation vial and dissolved in 17 mL Et_2O. The reaction was cooled to –78 °C using a liquid-nitrogen jacketed cold well, and *tert*-butyllithium (1.7 M in pentane, 1.0 mL, 1.7 mmol, 8.0 equiv.) was added dropwise. After 30 minutes, the vial was removed from the cold well and the solution was stirred at room temperature for 2 hours. A solution of **5-*carbonyl*-^{13}C** (52 mg, 0.213 mmol) in 0.1 mL Et_2O was then added dropwise, and the mixture was stirred for 5 minutes. The vial was removed from the glovebox while sealed, and a solution of HCl (2M in Et_2O, 0.85 mL) and MeOH (0.85 mL) was added while stirring. Solvents were removed by vacuum, and the crude residue was purified by silica flash column chromatography as noted.

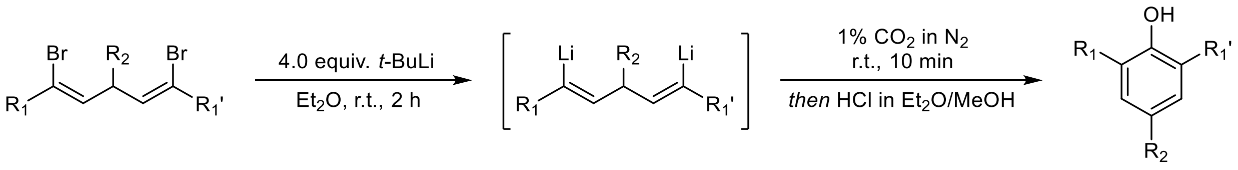

3 **4**

General procedure B for the synthesis of phenols from CO_2

In a glovebox under a nitrogen atmosphere, 0.425 mmol (2.0 equiv.) dibromide precursor **3** was added to a septum-capped 40 mL scintillation vial and dissolved in 17 mL Et_2O. The reaction was cooled to –78 °C using a liquid-nitrogen jacketed cold well, and *tert*-butyllithium (1.7 M in pentane, 1.0 mL, 1.7 mmol, 8.0 equiv.) was added dropwise. After 30 minutes, the vial was removed from the cold well and the solution was stirred at room temperature for 2 hours. The reaction mixture was then removed from the glovebox while sealed and 1% CO_2 in N_2 balance gas in a balloon was bubbled into the solution via 20G needle for 10 minutes. Afterwards, a solution of HCl (2M in Et_2O, 0.85 mL) and MeOH (0.85 mL) was added while stirring. Solvents were removed by vacuum, and the crude residue was purified by silica flash column chromatography as noted.

CAUTION: *Tert*-butyllithium solution in pentane is highly pyrophoric and must be handled with proper air-free technique. All manipulations for this study were performed on the smallest practical scale under a nitrogen atmosphere in a glovebox.

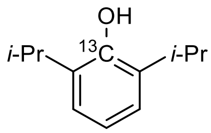

2,6-diisopropylphenol-*1*-^{13}C (**4a-*1*-^{13}C**)

Synthesized three times according to general procedure A from **3a** (132 mg, 0.425 mmol) with the following yields: (1) from 55 mg **5-*carbonyl*-^{13}C** (0.226 mmol) in 77% yield (31 mg, 0.173 mmol); (2) from 56 mg **5-*carbonyl*-^{13}C** (0.230 mmol) in 85% yield (35 mg, 0.195 mmol); (3) from 55 mg **5-*carbonyl*-^{13}C** (0.226 mmol) in 74% yield (30 mg, 0.167 mmol). Title compound was obtained after purification by silica flash column chromatography with an average yield of 79%. R_f = 0.43 (10% EtOAc in hexanes). *1*-^{13}C enrichment measured at 98.7% by quantitative ^{13}C NMR.

Scaled-up synthesis: Synthesized once according to a modified general procedure A. In a glovebox under a nitrogen atmosphere, **3a** (620 mg, 2.0 mmol) was added to a 200 mL Schlenk flask and dissolved in 80 mL Et_2O. The rest of the procedure was followed as is, using 4.7 mL *tert*-butyl lithium (1.7 M in pentane, 8.0 mmol) and 243 mg **5-*carbonyl*-^{13}C** (1.0 mmol). The title compound was obtained in 84% yield (150 mg, 0.836 mmol).

^{1}H NMR (400 MHz, $CDCl_3$) δ (ppm): 7.06 (dd, *J* = 9.1, 7.6 Hz, 2H), 6.91 (td, *J* = 7.6, 1.5 Hz, 1H), 4.77 (d, *J* = 3.0 Hz, 1H), 3.16 (hd, *J* = 6.9, 3.6 Hz, 2H), 1.28 (d, *J* = 6.8 Hz, 12H).

^{13}C{^{1}H} NMR (101 MHz, $CDCl_3$) δ (ppm): 149.9, 133.6 (d, *J* = 67 Hz), 123.4, 120.6, 27.2, 22.8.

HRMS (ESI-TOF) calculated 178.1318 for $C_{11}{}^{13}CH_{17}O^-$ [M-H]$^-$, found 178.1318. Error 0.2 ppm.

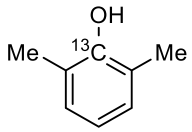

2,6-dimethylphenol-*1*-^{13}C (**4b-*1*-^{13}C**)

Synthesized twice according to general procedure A from **3b** (108 mg, 0.425 mmol) with the following yields: (1) from 52 mg **5-*carbonyl*-^{13}C** (0.213 mmol) in 60% yield (16 mg, 0.130 mmol); (2) from 54 mg **5-*carbonyl*-^{13}C** (0.222 mmol) in 62% yield (17 mg, 0.138 mmol). Title compound was obtained after purification by silica flash column chromatography with an average yield of 61%. R_f = 0.35 (25% CH_2Cl_2 in hexanes). *1*-^{13}C enrichment measured at 98.5% by quantitative ^{13}C NMR.

^{1}H NMR (400 MHz, $CDCl_3$) δ (ppm): 6.98 (t, *J* = 8.0 Hz, 2H), 6.76 (t, *J* = 7.5 Hz, 1H), 4.57 (d, *J* = 3.1 Hz, 1H), 2.25 (d, *J* = 4.0 Hz, 6H).

^{13}C{^{1}H} NMR (101 MHz, $CDCl_3$) δ (ppm): 151.2, 129.1 (d, *J* = 66.3 Hz), 126.7 (d, *J* = 1.9 Hz), 120.5 (d, *J* = 8.5 Hz), 14.0.

HRMS (ESI-TOF) calculated 122.0692 for $C_7{}^{13}CH_9O^-$ [M-H]$^-$, found 122.0687. Error 4.4 ppm.

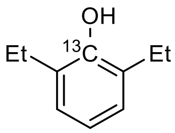

2,6-diethylphenol-*1*-^{13}C (**4c-*1*-^{13}C**)

Synthesized twice according to general procedure A from **3c** (120 mg, 0.425 mmol) with the following yields: (1) from 53 mg **5-*carbonyl*-^{13}C** (0.218 mmol) in 79% yield (26 mg, 0.172 mmol); (2) from 53 mg **5-*carbonyl*-^{13}C** (0.218 mmol) in 94% yield (31 mg, 0.205 mmol). Title compound was obtained after purification by silica flash column chromatography with an average yield of 87%. $\boldsymbol{R_f}$ = 0.20 (30% CH_2Cl_2 in hexanes). *1*-^{13}C enrichment measured at 99.3% by quantitative ^{13}C NMR.

^{1}H NMR (400 MHz, $CDCl_3$) δ (ppm): 7.01 (t, *J* = 8.2 Hz, 2H), 6.84 (td, *J* = 7.3, 1.7 Hz, 1H), 4.66 (d, *J* = 3.1 Hz, 1H), 2.64 (qd, *J* = 7.6, 4.0 Hz, 4H), 1.25 (t, *J* = 7.6 Hz, 6H).

^{13}C{^{1}H} NMR (101 MHz, $CDCl_3$) δ (ppm): 151.2, 129.1 (d, *J* = 66.3 Hz), 126.7 (d, *J* = 1.9 Hz), 120.5 (d, *J* = 8.5 Hz), 23.0, 14.0.

HRMS (ESI-TOF) calculated 150.1005 for $C_9{}^{13}CH_{13}O^-$ [M-H]$^-$, found 150.1005. Error 0.3 ppm.

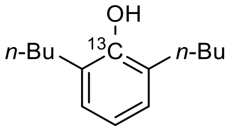

2,6-dibutylphenol-*1*-^{13}C (**4d-*1*-^{13}C**)

Synthesized twice according to general procedure A from **3d** (138 mg, 0.425 mmol) with the following yields: (1) from 55 mg **5-*carbonyl*-^{13}C** (0.226 mmol) in 30% yield (14 mg, 0.068 mmol); (2) from 52 mg **5-*carbonyl*-^{13}C** (0.214 mmol) in 41% yield (18 mg, 0.087 mmol). Title compound was obtained after purification by silica flash column chromatography with an average yield of 35%. $\boldsymbol{R_f}$ = 0.30 (20% CH_2Cl_2 in hexanes). *1*-^{13}C enrichment measured at 97.7% by quantitative ^{13}C NMR.

^{1}H NMR (400 MHz, $CDCl_3$) δ (ppm): 6.97 (dd, *J* = 8.9, 7.5 Hz, 2H), 6.80 (td, *J* = 6.5, 1.3 Hz, 1H), 4.62 (d, *J* = 3.1 Hz, 1H), 2.59 (td, *J* = 7.9, 4.1 Hz, 4H), 1.67 – 1.55 (m, 4H), 1.40 (h, *J* = 7.3 Hz, 4H), 0.95 (t, *J* = 7.3 Hz, 6H).

^{13}C{^{1}H} NMR (101 MHz, $CDCl_3$) δ (ppm): 151.5, 128.0 (d, *J* = 66.4 Hz), 127.8 (d, *J* = 1.9 Hz), 120.4 (d, *J* = 8.5 Hz), 32.1, 30.0, 22.8, 14.1.

HRMS (ESI-TOF) calculated 206.1631 for $C_{13}{}^{13}CH_{21}O^-$ [M-H]$^-$, found 206.1623. Error 4.1 ppm.

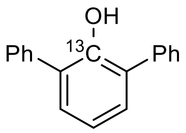

2,6-diphenylphenol-*1*-^{13}C (**4e-*1*-^{13}C**)

Synthesized according to general procedure A from **3e** (161 mg, 0.425 mmol) and **5-*carbonyl*-^{13}C** (54 mg, 0.222 mmol). Title compound was obtained in 70% yield (37 mg, 0.150 mmol) after purification by silica flash column chromatography. ***R_f*** = 0.20 (25% CH_2Cl_2 in hexanes). *1*-^{13}C enrichment measured at 98.0% by quantitative ^{13}C NMR.

^{1}H NMR (400 MHz, $CDCl_3$) δ (ppm): 7.58 (d, J = 7.6 Hz, 4H), 7.49 (t, J = 7.6 Hz, 4H), 7.40 (t, J = 7.4 Hz, 2H), 7.30 (t, J = 8.1 Hz, 2H), 7.08 (t, J = 7.6 Hz, 1H) 5.42 (d, *J* = 3.3 Hz, 1H).

^{13}C{^{1}H} NMR (101 MHz, $CDCl_3$) δ (ppm): 149.5, 137.7, 130.1, 129.5, 129.0, 128.9 (d, *J* = 67.5 Hz), 127.8, 120.8 (d, *J* = 8.1 Hz).

HRMS (ESI-TOF) calculated 246.1005 for $C_{17}{}^{13}CH_{13}O^-$ [M-H]$^-$, found 246.1006. Error 0.4 ppm.

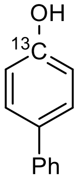

4-phenylphenol-*1*-^{13}C (**4f-*1*-^{13}C**)

Synthesized according to general procedure A from **3f** (110 mg, 0.364 mmol) and **5-*carbonyl*-^{13}C** (52 mg, 0.213 mmol). Title compound was obtained in 74% yield (23 mg, 0.134 mmol) after purification by silica flash column chromatography. ***R_f*** = 0.47 (20% EtOAc in hexanes). *1*-^{13}C enrichment measured at 99.2% by quantitative ^{13}C NMR.

^{1}H NMR (400 MHz, $CDCl_3$) δ (ppm): 7.54 (d, *J* = 7.0 Hz, 2H), 7.51 – 7.45 (m, 2H), 7.41 (t, *J* = 7.6 Hz, 2H), 7.30 (tt, *J* = 7.4, 1.3 Hz, 1H), 6.91 (dd, *J* = 8.8, 2.8 Hz, 2H), 4.70 (d, *J* = 3.6 Hz, 1H).

^{13}C{^{1}H} NMR (101 MHz, $CDCl_3$) δ (ppm): 155.2, 140.9, 134.2 (d, *J* = 8.9 Hz), 128.9, 128.6, 126.9, 126.9 115.8 (d, *J* = 65.9 Hz).

HRMS (ESI-TOF) calculated 170.0692 for $C_{11}{}^{13}CH_9O^-$ [M-H]$^-$, found 170.0682. Error 6.1 ppm.

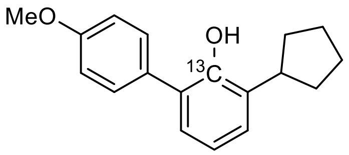

2-cyclopentyl-6-(4-methoxyphenyl)phenol-*1*-^{13}C (**4g-*1*-^{13}C**)

Synthesized according to general procedure A from **3g** (170 mg, 0.425 mmol) and **5-*carbonyl*-^{13}C** (52 mg, 0.213 mmol). Title compound was obtained in 43% yield (25 mg, 0.0928 mmol) after purification by silica flash column chromatography. $\boldsymbol{R_f}$ = 0.35 (50% CH_2Cl_2 in hexanes). *1*-^{13}C enrichment measured at 99.0% by quantitative ^{13}C NMR.

^{1}H NMR (400 MHz, $CDCl_3$) δ (ppm): 7.39 (d, *J* = 8.8 Hz, 2H), 7.21 (ddd, *J* = 9.4, 7.6, 1.9 Hz, 1H), 7.10 – 6.98 (m, 3H), 6.92 (td, *J* = 7.5, 1.6 Hz, 1H), 5.25 (d, *J* = 3.4 Hz, 1H), 3.86 (s, 3H), 3.41 – 3.29 (m, 1H), 2.18 – 1.58 (m, 8H).

^{13}C{^{1}H} NMR (101 MHz, $CDCl_3$) δ (ppm): 159.5, 150.5, 132.6 (d, *J* = 68.2 Hz), 130.6 (d, *J* = 1.9 Hz), 129.6, 127.7, 127.6 (d, *J* = 65.2 Hz), 126.4, 120.3 (d, *J* = 8.5 Hz), 114.9, 55.5, 39.6, 33.1, 25.6.

HRMS (ESI-TOF) calculated 268.1424 for $C_{17}{}^{13}CH_{19}O_2^-$ [M-H]$^-$, found 268.1416. Error 3.0 ppm.

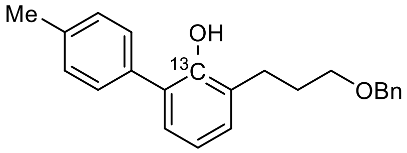

2-(3-(benzyloxy)propyl)-6-(*p*-tolyl)phenol-*1*-^{13}C (**4h-*1*-^{13}C**)

Synthesized twice according to general procedure A from **3h** (197 mg, 0.425 mmol) with the following yields: (1) from 52 mg **5-*carbonyl*-^{13}C** (0.214 mmol) in 34% yield (24 mg, 0.072 mmol); (2) from 51 mg **5-*carbonyl*-^{13}C** (0.210 mmol) in 43% yield (30 mg, 0.090 mmol). Title compound was obtained after purification by silica flash column chromatography with an average yield of 39%. $\boldsymbol{R_f}$ = 0.05 (25% CH_2Cl_2 in hexanes). *1*-^{13}C enrichment measured at 99.0% by quantitative ^{13}C NMR.

^{1}H NMR (400 MHz, $CDCl_3$) δ (ppm): 7.44 – 7.25 (m, 9H), 7.14 – 7.07 (m, 2H), 6.90 (td, *J* = 7.5, 1.7 Hz, 1H), 6.00 (d, *J* = 3.2 Hz, 1H), 4.55 (s, 2H), 3.53 (t, *J* = 6.2 Hz, 2H), 2.80 (td, *J* = 7.3, 4.4 Hz, 2H), 2.41 (s, 3H), 1.98 (p, *J* = 6.3 Hz, 2H).

^{13}C{^{1}H} NMR (101 MHz, $CDCl_3$) δ (ppm): 151.2, 138.3, 137.4, 135.0, 129.8, 129.3 (d, *J* = 1.9 Hz), 128.5 (d, J = 66.2 Hz), 128.5, 128.4 (d, J = 1.5 Hz), 128.2 (d, J = 66.6 Hz), 128.0, 127.8, 120.4, 120.3, 73.1, 69.2, 29.9, 26.7, 21.4.

HRMS (ESI-TOF) calculated 332.1737 for $C_{22}{}^{13}CH_{23}O_2^-$ [M-H]$^-$, found 332.1731. Error 1.8 ppm.

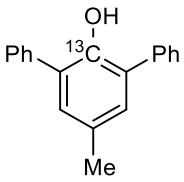

4-methyl-2,6-diphenylphenol-*1*-^{13}C (**4i-*1*-^{13}C**)

Synthesized according to general procedure A from **3i** (167 mg, 0.425 mmol) and **5-*carbonyl*-^{13}C** (54 mg, 0.222 mmol). Title compound was obtained in 82% yield (47 mg) after purification by silica flash column chromatography. R_f = 0.33 (25% CH_2Cl_2 in hexanes). *1*-^{13}C enrichment measured at 96.3% by quantitative ^{13}C NMR.

^{1}H NMR (400 MHz, $CDCl_3$) δ (ppm): 7.56 (d, *J* = 7.0 Hz, 4H), 7.47 (t, *J* = 7.7 Hz, 4H), 7.38 (t, *J* = 7.4 Hz, 2H), 7.10 (d, *J* = 8.6 Hz, 2H), 5.24 (d, *J* = 3.3 Hz, 1H), 2.37 (s, 3H).

^{13}C{^{1}H} NMR (101 MHz, $CDCl_3$) δ (ppm): 147.2, 137.9, 130.6, 129.9 (d, *J* = 7.7 Hz), 129.5, 128.9, 128.5 (d, *J* = 68.0 Hz), 127.7, 20.5.

HRMS (ESI-TOF) calculated 260.1162 for $C_{18}{}^{13}CH_{15}O^-$ [M-H]$^-$, found 260.1153. Error 3.4 ppm.

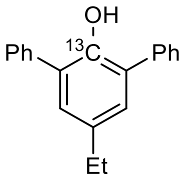

4-ethyl-2,6-diphenylphenol-*1*-^{13}C (**4j-*1*-^{13}C**)

Synthesized according to general procedure A from **3j** (173 mg, 0.425 mmol) and **5-*carbonyl*-^{13}C** (54 mg, 0.222 mmol). Title compound was obtained in 98% yield (60 mg) after purification by silica flash column chromatography. R_f = 0.30 (25% CH_2Cl_2 in hexanes). *1*-^{13}C enrichment measured at 97.8% by quantitative ^{13}C NMR.

^{1}H NMR (400 MHz, $CDCl_3$) δ (ppm): 7.58 (d, *J* = 6.7 Hz, 4H), 7.49 (t, *J* = 7.6 Hz, 4H), 7.40 (t, *J* = 7.4 Hz, 2H), 7.14 (d, *J* = 8.6 Hz, 2H), 5.28 (d, *J* = 3.3 Hz, 1H), 2.69 (q, *J* = 7.6 Hz, 2H), 1.30 (t, *J* = 7.6 Hz, 3H).

^{13}C{^{1}H} NMR (101 MHz, $CDCl_3$) δ (ppm): 147.4, 138.0, 136.4 (d, *J* = 7.3 Hz), 129.5, 129.5, 128.9, 128.7 (d, *J* = 67.5 Hz), 127.7, 28.2, 16.0.

HRMS (ESI-TOF) calculated 274.1318 for $C_{19}{}^{13}CH_{17}O^-$ [M-H]$^-$, found 274.1344. Error 9.3 ppm.

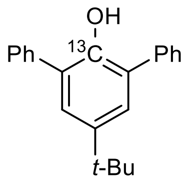

4-(*tert*-butyl)-2,6-diphenylphenol-*1*-^{13}C (**4k-*1*-^{13}C**)

Synthesized according to general procedure A from **3k** (185 mg, 0.425 mmol) and **5-*carbonyl*-^{13}C** (51 mg, 0.210 mmol). Title compound was obtained in 64% yield (41 mg) after purification by silica flash column chromatography. $\boldsymbol{R_f}$ = 0.30 (25% CH_2Cl_2 in hexanes). *1*-^{13}C enrichment measured at 98.2% by quantitative ^{13}C NMR.

^{1}H NMR (400 MHz, $CDCl_3$) δ (ppm): 7.61 – 7.54 (m, 4H), 7.54 – 7.43 (m, 4H), 7.43 – 7.36 (m, 2H), 7.30 (d, *J* = 8.6 Hz, 2H), 5.27 (d, *J* = 3.2 Hz, 1H), 1.37 (s, 9H).

^{13}C{^{1}H} NMR (101 MHz, $CDCl_3$) δ (ppm): 147.2, 143.5 (d, *J* = 6.6 Hz), 138.3, 129.6 (d, *J* = 1.9 Hz), 129.0, 128.2 (d, *J* = 67.4 Hz), 127.7, 127.2 (d, *J* = 1.5 Hz), 34.4, 31.7.

HRMS (ESI-TOF) calculated 302.1631 for $C_{21}{}^{13}CH_{21}O^-$ [M-H]$^-$, found 302.1649. Error 5.8 ppm.

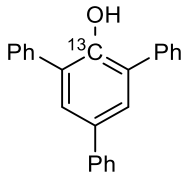

2,4,6-triphenylphenol-*1*-^{13}C (**4l-*1*-^{13}C**)

Synthesized according to general procedure A from **3l** (193 mg, 0.425 mmol) and **5-*carbonyl*-^{13}C** (53 mg, 0.218 mmol). Title compound was obtained as a yellow solid in 65% yield (46 mg) after purification by silica flash column chromatography. $\boldsymbol{R_f}$ = 0.35 (25% CH_2Cl_2 in hexanes). *1*-^{13}C enrichment measured at 98.3% by quantitative ^{13}C NMR.

^{1}H NMR (400 MHz, $CDCl_3$) δ (ppm): 7.62 (d, *J* = 8.3 Hz, 6H), 7.55 – 7.47 (m, 6H), 7.45 – 7.39 (m, 4H), 7.32 (t, *J* = 7.3 Hz, 1H), 5.44 (d, *J* = 3.4 Hz, 1H).

^{13}C{^{1}H} NMR (101 MHz, $CDCl_3$) δ (ppm): 149.1, 140.7, 137.7, 134.0 (d, *J* = 7.3 Hz), 129.5 (d, *J* = 1.5 Hz), 129.2 (d, *J* = 68.0 Hz), 129.1, 128.9, 128.8, 128.0, 127.0, 126.9.

HRMS (ESI-TOF) calculated 322.1318 for $C_{23}{}^{13}CH_{17}O^-$ [M-H]$^-$, found 322.1321. Error 0.7 ppm.

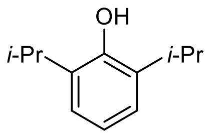

2,6-diisopropylphenol (**4a**)

Synthesized according to general procedure B from **3a** (132 mg, 0.425 mmol) and 1% CO_2 in N_2. Title compound was obtained in 24% yield (18 mg, 0.102 mmol) after purification by silica flash column chromatography. $\boldsymbol{R_f}$ = 0.43 (10% EtOAc in hexanes).

^{1}H NMR (400 MHz, $CDCl_3$) δ (ppm): 7.07 (d, *J* = 7.6 Hz, 2H), 6.91 (t, *J* = 7.6 Hz, 1H), 4.77 (s, 1H), 3.16 (hept, *J* = 6.9 Hz, 2H), 1.28 (d, *J* = 6.9 Hz, 12H).

Spectroscopic data agree with those in the literature.[1]

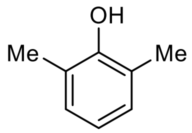

2,6-dimethylphenol (**4b**)

Synthesized according to general procedure B from **3b** (108 mg, 0.425 mmol) and 1% CO_2 in N_2. Title compound was obtained in 25% yield (13 mg, 0.106 mmol) after purification by silica flash column chromatography. $\boldsymbol{R_f}$ = 0.35 (25% CH_2Cl_2 in hexanes).

^{1}H NMR (400 MHz, $CDCl_3$) δ (ppm): 6.98 (d, *J* = 7.4 Hz, 2H), 6.76 (t, *J* = 7.5 Hz, 1H), 4.58 (s, 1H), 2.25 (s, 6H).

Spectroscopic data agree with those in the literature.[1]

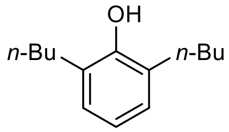

2,6-dibutylphenol (**4d**)

Synthesized according to general procedure B from **3d** (144 mg, 0.425 mmol) and 1% CO_2 in N_2. Title compound was obtained in 11% yield (10 mg, 0.0485 mmol) after purification by silica flash column chromatography. $\boldsymbol{R_f}$ = 0.30 (20% CH_2Cl_2 in hexanes).

^{1}H NMR (400 MHz, $CDCl_3$) δ (ppm): 6.97 (d, *J* = 7.5 Hz, 2H), 6.80 (t, *J* = 7.5 Hz, 1H), 4.62 (d, *J* = 1.2 Hz, 1H), 2.64 – 2.55 (m, 4H), 1.60 (tt, *J* = 7.9, 6.4 Hz, 4H), 1.40 (h, *J* = 7.4 Hz, 4H), 0.95 (t, *J* = 7.3 Hz, 6H).

Spectroscopic data agree with those in the literature.[2]

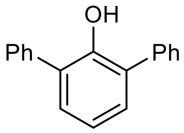

2,6-diphenylphenol (**4e**)

Synthesized according to general procedure B from **3e** (161 mg, 0.425 mmol) and 1% CO_2 in N_2. Title compound was obtained in 57% yield (60 mg, 0.242 mmol) after purification by silica flash column chromatography. $\boldsymbol{R_f}$ = 0.20 (25% CH_2Cl_2 in hexanes)

^{1}H NMR (400 MHz, $CDCl_3$) δ (ppm): 7.60 – 7.55 (m, 4H), 7.49 (t, *J* = 7.5 Hz, 4H), 7.40 (t, *J* = 7.3 Hz, 2H), 7.30 (d, *J* = 7.6 Hz, 2H), 7.08 (dd, *J* = 7.9, 7.2 Hz, 1H), 5.41 (s, 1H).

Spectroscopic data agree with those in the literature.[3]

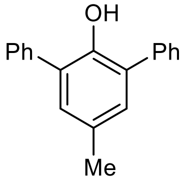

4-methyl-2,6-diphenylphenol (**4i**)

Synthesized according to general procedure B from **3i** (167 mg, 0.425 mmol) and 1% CO_2 in N_2. Title compound was obtained in 40% yield (44 mg, 0.170 mmol) after purification by silica flash column chromatography. $\boldsymbol{R_f}$ = 0.33 (25% CH_2Cl_2 in hexanes).

^{1}H NMR (400 MHz, $CDCl_3$) δ (ppm): 7.56 (d, *J* = 7.0 Hz, 4H), 7.47 (t, *J* = 7.7 Hz, 4H), 7.38 (t, *J* = 7.4 Hz, 2H), 7.10 (s, 2H), 5.24 (s, 1H), 2.36 (s, 3H).

Spectroscopic data agree with those in the literature.[4]

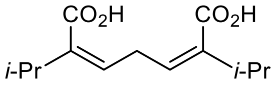

(2*Z*,5*Z*)-2,6-diisopropylhepta-2,5-dienedioic acid (**6**)

Synthesized analogously to general procedure B from **3a** (264 mg, 0.85 mmol) and 100% CO_2. Title compound was obtained in 52% yield (105 mg, 0.44 mmol) after purification by silica flash column chromatography. $\boldsymbol{R_f}$ = 0.20 (80% EtOAc in hexanes).

^{1}H NMR (500 MHz, $CDCl_3$) δ (ppm): 12.65 (br s, 2H), 5.86 (t, *J* = 7.8 Hz, 2H), 3.59 (t, *J* = 8.0 Hz, 2H), 2.75 (h, *J* = 6.8 Hz, 2H), 1.09 (d, *J* = 7.0 Hz, 12H).

^{13}C NMR (126 MHz, $CDCl_3$) δ (ppm): 175.3, 139.0, 134.0, 31.4, 30.7, 21.9

HRMS (ESI-TOF) calculated 275.1056 for $C_{13}H_{20}O_4^-$ $[M+Cl]^-$, found 275.1064. Error 3.1 ppm.

Optimization of Reaction Conditions

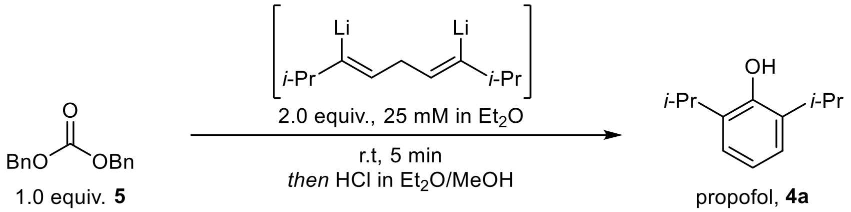

Standard conditions for optimization of phenol cyclization from dibenzyl carbonate

In a glovebox under a nitrogen atmosphere, dibromide precursor **3a** (26.4 mg, 0.085 mmol, 2.0 equiv.) was added to a two-dram vial and dissolved in 3.2 mL Et_2O. The reaction was cooled to –78 °C using a liquid-nitrogen jacketed cold well, and *tert*-butyllithium (1.7 M in pentane, 0.2 mL, 0.34 mmol, 8.0 equiv.) was added dropwise. After 30 minutes, the vial was removed from the cold well and the solution was stirred at room temperature for 2 hours. A solution of dibenzyl carbonate (**5**, 10.3 mg,0.0425 mmol) in 0.1 mL Et_2O was then added dropwise, and the mixture was stirred for 5 minutes. The vial was removed from the glovebox, and a solution of HCl (2M in Et_2O, 0.2 mL) and MeOH (0.2 mL) was added while stirring. Solvents were removed by vacuum, and the crude residue was dissolved in 0.50 mL CD_3OD and 0.20 mL $CDCl_3$. A standard of 3.3 mg mesitylene was added for quantitation by ^1H NMR.

Representative ^1H qNMR (400 MHz, CD_3OD) spectrum for crude reaction under standard conditions:

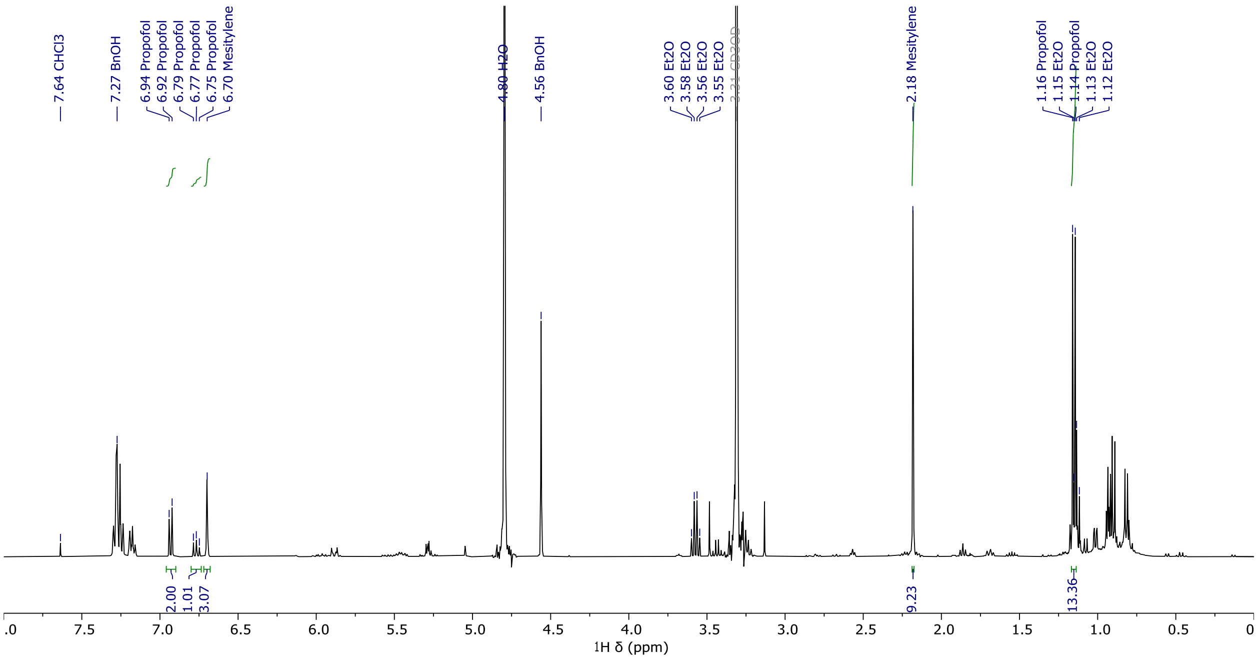

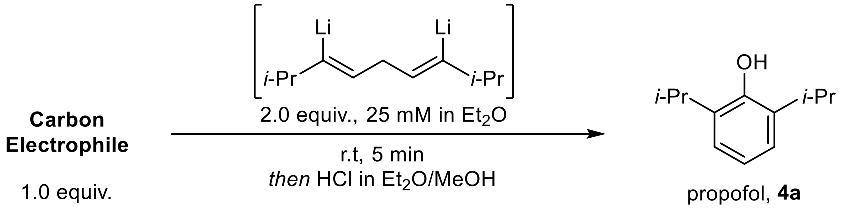

Entry	Carbon Electrophile	NMR Yield 4a (%)
1 (std)	Dibenzyl carbonate (**5**)	56.5 ± 7.0
2	Diethyl carbonate	53.9
3	Diphenyl carbonate	33.5
4	Methyl chloroformate	52.6
5	Carbonyl diimidazole	0

Table S1. Optimization of carbon electrophile.

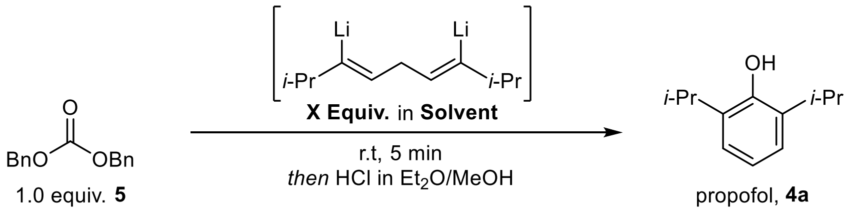

Entry	Equiv. Lithiate	Concentration	Solvent	NMR Yield 4a (%)
1	1.0	10 mM	Et_2O	37.0
2	1.5	15 mM	Et_2O	47.3
3	2.0	20 mM	Et_2O	52.9
4 (std)	2.0	25 mM	Et_2O	56.5 ± 7.0
5	2.5	25 mM	Et_2O	59.7
6	2.5	30 mM	Et_2O	45.0
7	2.0	25 mM	Pentane	0
8	2.0	25 mM	THF	22.6
9	2.0	25 mM	Toluene	0
10	2.0	25 mM	1/1 Et_2O/Pentane	30.4
11	2.0	25 mM	1/1 Et_2O/THF	16.8

Table S2. Optimization of reaction equivalents, concentration, and solvent.

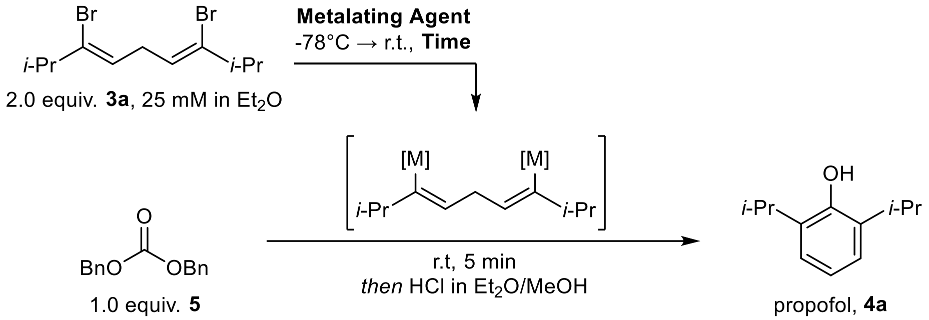

Entry	Metalating Agent	Room Temp Time	NMR Yield 4a (%)
1 (std)	8.0 equiv. *t*-BuLi	2 hr	56.5 ± 7.0
2	8.0 equiv. *t*-BuLi	1 hr	50.1
3	8.0 equiv. *t*-BuLi	10 min	3.9
4	4.0 equiv. *n*-BuLi	2 hr	0
5	4.0 equiv. *s*-BuLi	2 hr	10.1
6	2.0 equiv. *i*-Pr(*n*-Bu)$_2$MgLi·LiCl in THF	2 hr	0
7	2.0 equiv. *i*-Pr(*n*-Bu)$_2$MgLi·LiCl in Et_2O	2 hr	0

Table S3. Optimization of halogen-metal exchange conditions of dibromide precursor.

Carbon-11 Radiochemistry

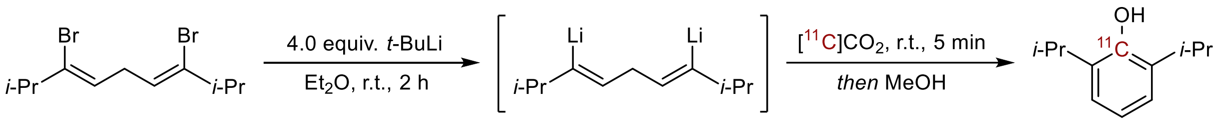

3a **[11C]4a**

Optimized radiosynthesis of [*1*-^{11}C]4a ([*1*-^{11}C]propofol) from cyclotron produced [^{11}C]CO_2

The corresponding dilithiate precursor was prepared analogously to general procedure A from dibromide **3a**. An 800 µL (20 µmol) aliquot of the reaction mixture was transferred to an oven-dried 2 mL v-vial, equipped with magnetic stir bar, and sealed with a screw-cap PTFE septum and electrical tape. The vial was kept under a static atmosphere of dry nitrogen gas.

[^{11}C]CO_2 (100–300 mCi) was produced via the ^{14}N(p,α)^{11}C nuclear reaction from a 2–5 min × 52 µA irradiation with 16.5 MeV protons. The cyclotron-produced [^{11}C]CO_2 was concentrated on molecular sieves using a GE Healthcare TRACERlab FX MeI module. The [^{11}C]CO_2 was then released at 350 °C in a stream of helium and bubbled directly into the reaction vial for 1 minute. The vial was vented through Ascarite® (sodium hydroxide coated silica) to trap unreacted [^{11}C]CO_2. The reaction was allowed to stir for 4 more minutes then quenched with an equal volume of methanol. A 10 µL aliquot of the crude reaction mixture was injected directly into the analytical HPLC to measure radiochemical conversion (RCC). For isolation, the crude reaction mixture was injected directly into the semi-prep HPLC. The isolated [*1*-^{11}C]**4a** was collected in >99% radiochemical purity (RCP) and chemical identity was confirmed with co-injection of commercial reference compound.

Entry		Precursor solution	TE (%)	HPLC Purity (%)	RCC (%)
1	*n* = 2	400 uL, 2.5 µmol	31.2	0	0
2	*n* = 2	400 µL, 10 µmol	59.4	25.2	14.8
3	*n* = 1	800 µL, 20 µmol	68.8	30.8	21.3

Table S4. Optimization of precursor loading on radiochemical conversion (RCC) of [*1*-^{11}C]**4a** ([^{11}C]propofol).

Entry	Total activity at EOB (GBq)	TE (%)	Activity in isolated product (MBq)	Synthesis time (min)	d.c. RCY (%)	minimum A_m (GBq/µmol)
1	9.30	76.9	59.2	26	1.5	> 34.6
2	10.2	49.0	154	28	3.9	> 37.8
Avg		62.9		27	2.7	

Table S5. Isolation of [*1*-^{11}C]**4a** ([^{11}C]propofol). Radiochemical yields (RCY) decay-corrected to end of bombardment (EOB).

HPLC information

Radioactivity was measured via flow radiation detector (Carroll & Ramsey Model 105S). The UV detection wavelength used for HPLC measurements was 210 nm.

The analytical HPLC used was Agilent 1200 series with an Agilent Eclipse XDB-C18 5 µm, 4.6 x 150 mm, 80Å column with the following solvent system: 2 mL/min MeCN [50% (0–10 min); 50-95% (10–11 min); 95% (11–13 min); 95-5% (13–14 min) and 50% (14–15 min)] in H_2O (+ 0.1% TFA).

Elution time of **4a** (propofol) under this system is 7.3 min (UV), 7.5 min (Radioactivity).

The semi-prep HPLC used was Agilent 1200 series with a Phenomenex Luna C18(2) 5 µm, 10 x 250 mm, 100Å column with the following solvent system: 5 mL/min 70% MeCN in H_2O (+ 0.1% TFA).

General radiochemistry calculations

The radioactivity of carbon-11 was measured using a dose calibrator (Capintec CRC-25R), and radioactivity measurements were corrected for physical decay of carbon-11 ($t_{1/2}$ = 20.4 min).

Trapping efficiency (TE) was calculated by dividing the radioactivity trapped in the reaction vial by the total amount of radioactivity:

$$\mathrm{TE} = \frac{\text{Radioactivity in reaction vial}}{\text{Total } ^{11}\text{C radioactivity}}$$

Radiochemical conversion (RCC) was calculated by multiplying the HPLC radiochemical purity by the trapping efficiency:

$$\mathrm{RCC} = \text{HPLC Purity} \times \mathrm{TE}$$

Isolated radiochemical yield (RCY) was calculated by dividing the radioactivity collected from the HPLC by the total amount of radioactivity in the reaction vial and ascarite and decay-corrected to end of bombardment.

Molar activity measurement

The minimum molar activity (A_m) of [*1*-^{11}C]**4a** was estimated by injecting an aliquot of the isolated product into the analytical HPLC. The radioactivity in the aliquot was measured via dose-calibrator before injection and decay-corrected to end of synthesis time (EOS) to determine the activity concentration of the isolated product. Due the low absorptivity of **4a**, no significant UV signal was observed for aliquots of the isolated product. A calibration curve was made by injecting a known amount of reference compound to determine the limit of detection under our HPLC conditions of 0.561 nmol/mL.

$$A_m\ (\text{GBq/µmol}) > \frac{\text{Activity Concentration (GBq/mL)}}{5.61 \times 10^{-4}\ \text{µmol/mL}}$$

Representative crude analytical HPLC trace

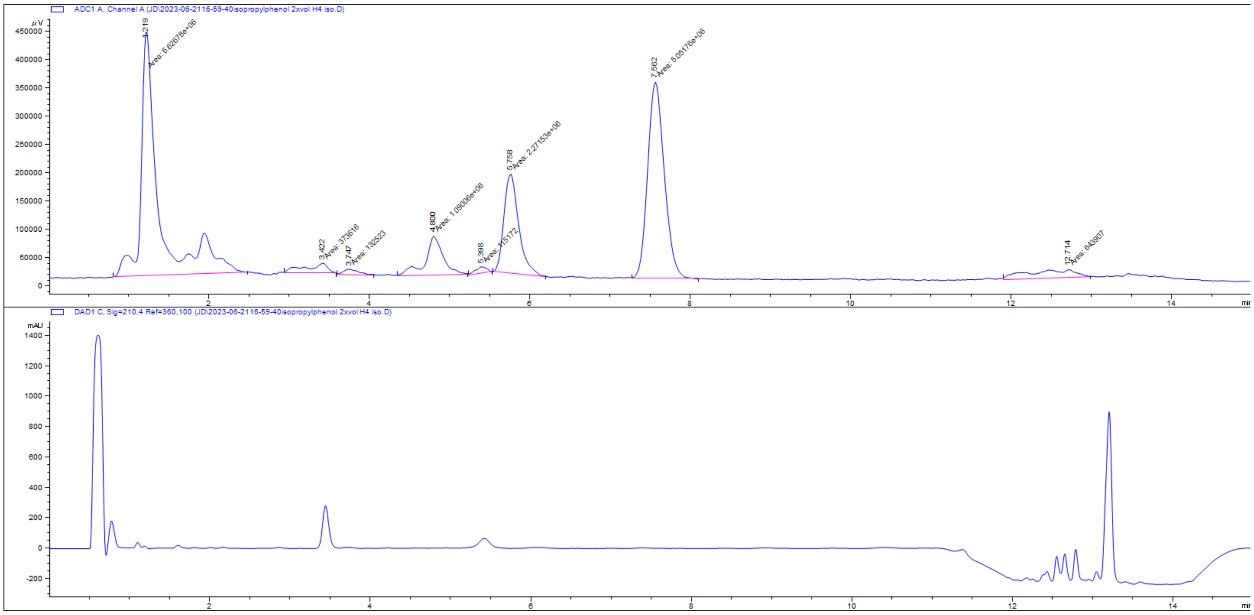

Top: Radioactivity; Bottom: 210 nm UV

Radioactivity integration table:

#	Time	Type	Area	Height	Width	Area%	Symmetry
1	0.979	MF	414844.7	38291.5	0.1806	2.514	1.012
2	1.219	MF	4802661.5	436389.3	0.1834	29.099	0.515
3	1.738	MF	385200.8	37027	0.1734	2.334	1.003
4	1.938	MF	722838.7	72988.7	0.1651	4.380	0.72
5	2.146	FM	312111.7	26950	0.193	1.891	0.472
6	4.526	MF	181080.6	16508.9	0.1828	1.097	0.884
7	4.8	FM	917859.3	69813.8	0.2191	5.561	0.586
8	5.398	MM	95059.3	10435	0.1518	0.576	1.165
9	5.758	MM	2250163.8	176000.7	0.2131	13.634	0.799
10	7.562	MM	5086976.5	349218.7	0.2428	30.822	0.818
11	12.714	MM	1335727.1	18079.6	1.2313	8.093	0.83

Isolated [*1*-^{11}C]4a analytical HPLC trace

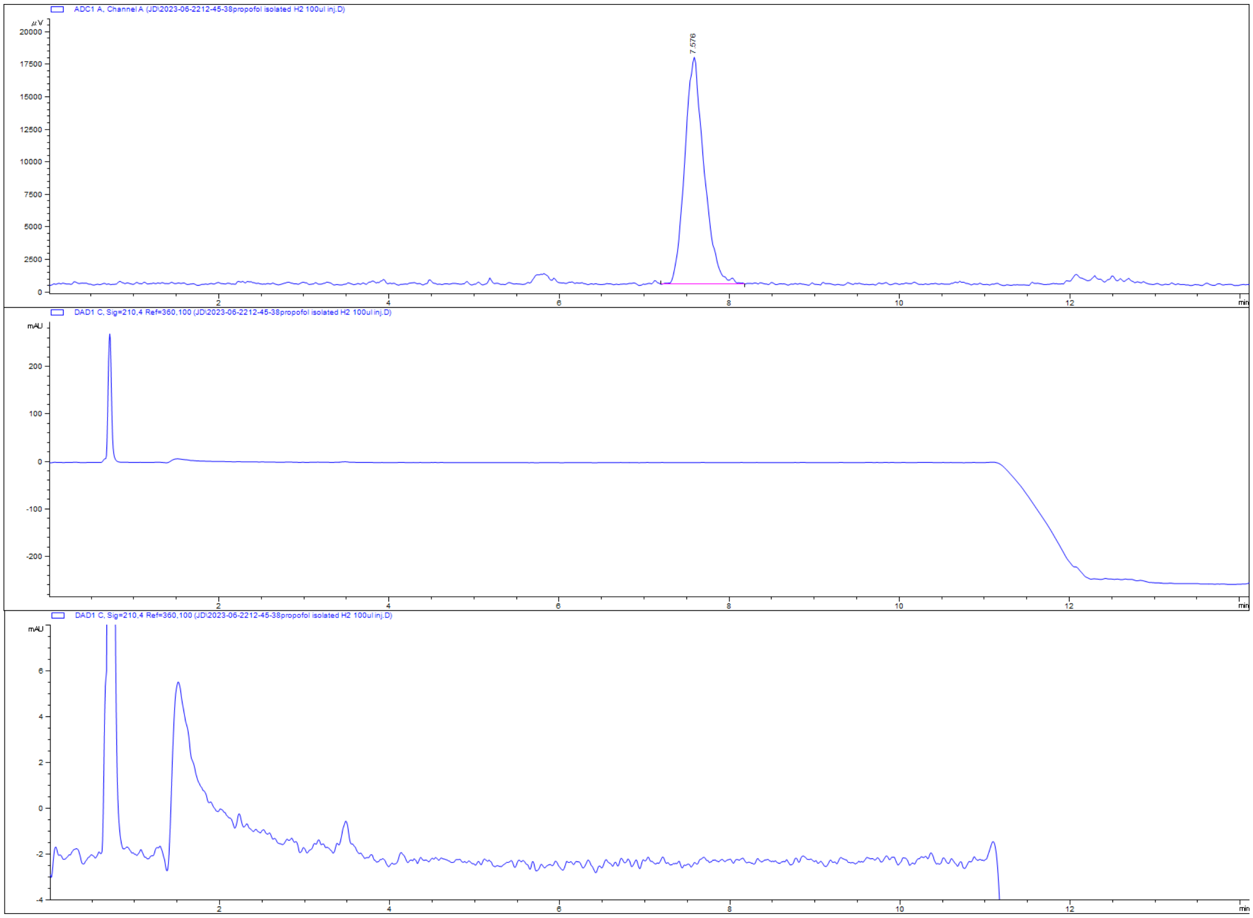

Top: Radioactivity; Middle: 210 nm UV scaled; Bottom: 210 nm UV zoomed

Co-injection of isolated [*1*-^{11}C]4a with reference compound

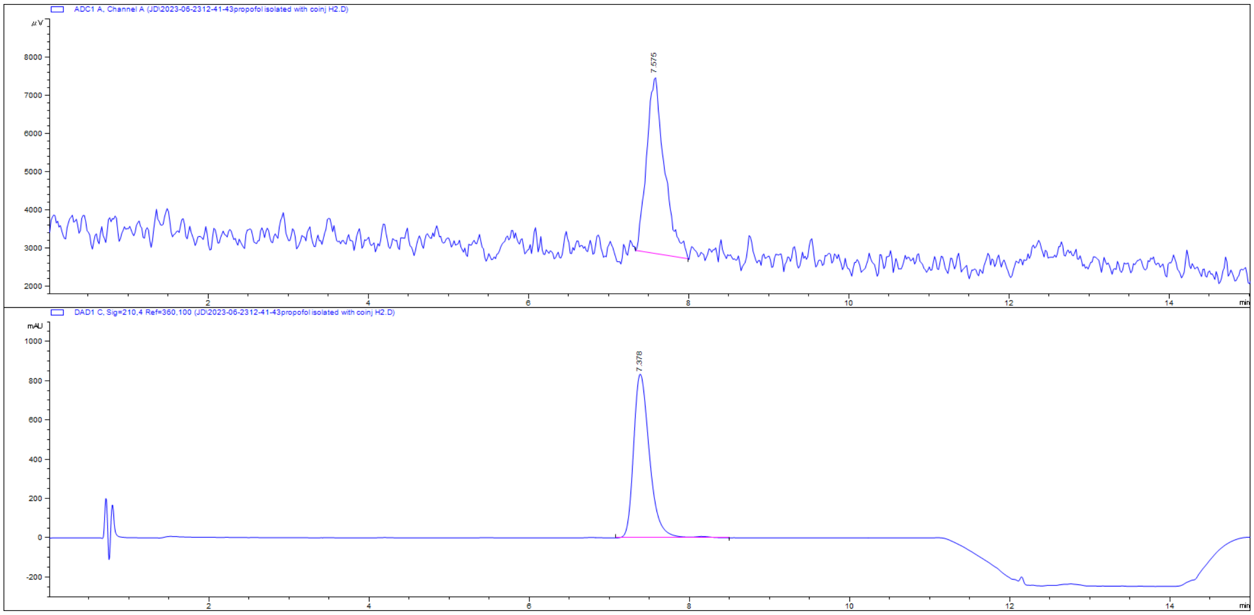

Top: Radioactivity; Bottom: 210 nm UV

Synthesis of Dibenzyl Carbonate from Carbonate Salts

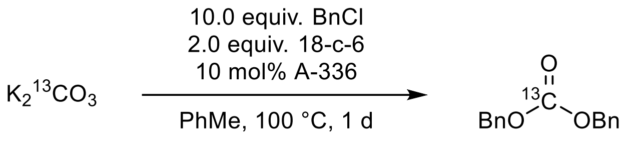

5-*carbonyl*-^{13}C

Synthesis of dibenzyl carbonate-*carbonyl*-^{13}C (5-*carbonyl*-^{13}C) from potassium carbonate-^{13}C

To an oven-dried 100 mL Kontes®-topped Schlenk flask equipped with a magnetic stir bar were added potassium carbonate-^{13}C (696 mg, 5.0 mmol), 18-crown-6 (2.64 g, 10.0 mmol), Aliquat-336® (202 mg, 0.50 mmol), benzyl chloride (6.33 g, 50.0 mmol), and toluene (6.0 mL) under nitrogen atmosphere and sealed. The reaction mixture was allowed to stir in a 100 °C oil bath for 24 hours. Afterwards, the reaction mixture was filtered over a pad of silica and the crude filtrate was concentrated under high vacuum. The title compound was isolated in 72% yield as a low-melting point white solid (860 mg, 3.54 mmol) by silica flash column chromatography. $\boldsymbol{R_f}$ = 0.65 (20% EtOAc in hexanes). *1*-^{13}C enrichment measured at 98.3% by quantitative ^{13}C NMR.

^{1}H NMR (400 MHz, $CDCl_3$) δ (ppm): 7.40 – 7.30 (m, 10H), 5.18 (d, *J* = 3.4 Hz, 4H).

^{13}C{^{1}H} NMR (101 MHz, $CDCl_3$) δ (ppm): 155.2, 135.3 (d, *J* = 2.3 Hz), 128.7, 128.7, 128.5, 69.9 (d, *J* = 1.5 Hz).

HRMS (ESI-TOF) calculated 266.0869 for $C_{14}{}^{13}CH_{14}NaO_3^+$ [M+Na]$^+$, found 266.0860. Error 3.3 ppm.

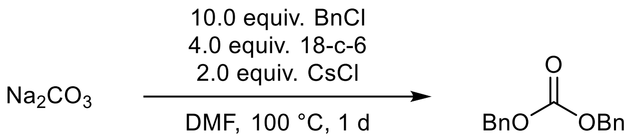

5

Synthesis of dibenzyl carbonate (5) from sodium carbonate.

To an oven-dried 100 mL Kontes®-topped Schlenk flask equipped with magnetic stir bar were added sodium carbonate (106 mg, 1.0 mmol), 18-crown-6 (1.06 g, 4.0 mmol), cesium chloride (337 mg, 2.0 mmol), benzyl chloride (1.27 g, 10.0 mmol), and *N*,*N*-dimethylformamide (1.0 mL) under nitrogen atmosphere and sealed. The reaction mixture was allowed to stir in a 100 °C oil bath for 24 hours. Afterwards, the reaction mixture was added to 50 mL brine, and the crude product was extracted with EtOAc (3 x 25 mL). The combined organic fractions were dried over anhydrous Na_2SO_4, filtered, and concentrated under reduced pressure. The title compound was isolated as a white solid in 30% yield (73 mg, 0.30 mmol) by silica flash column chromatography. $\boldsymbol{R_f}$ = 0.65 (20% EtOAc in hexanes).

^{1}H NMR (400 MHz, $CDCl_3$) δ (ppm): 7.44 – 7.34 (m, 10H), 5.18 (s, 4H).

Spectroscopic data agree with those in the literature.[5]

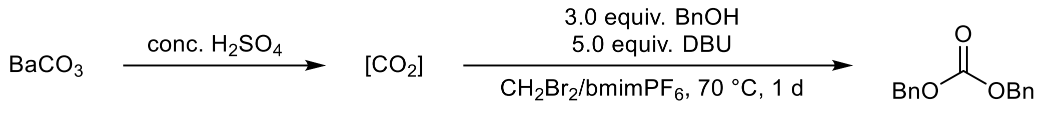

5

Synthesis of dibenzyl carbonate (5) from barium carbonate.

An oven-dried 20 mL COware two-chamber flask was evacuated and filled with dry N_2 gas. On one side of the flask, benzyl alcohol (162 mg, 1.5 mmol), 1,8-diazabicyclo[5.4.0]undec-7-ene (DBU, 381 mg, 2.5 mmol), and 0.1 mL 1-butyl-3-methylimidazolium hexafluorophosphate (bmimPF_6) were dissolved in 1.0 mL dibromomethane and equipped with a stir bar. On the other side of the flask was barium carbonate (99 mg, 0.5 mmol), and the entire flask was placed under 1 atm of static N_2 gas. Carefully, 300 µL concentrated sulfuric acid was added dropwise to the barium carbonate. Then the flask was placed in a 70 °C oil bath, and the reaction mixture was allowed to stir for 24 hours. Afterwards, the reaction mixture was filtered over a pad of silica and the crude filtrate was concentrated under high vacuum. The title compound was isolated as a white solid in 18% yield (22 mg, 0.09 mmol) by silica flash column chromatography. $\boldsymbol{R_f}$ = 0.65 (20% EtOAc in hexanes). Spectroscopic data as above.

Synthesis of Dibromide Precursors

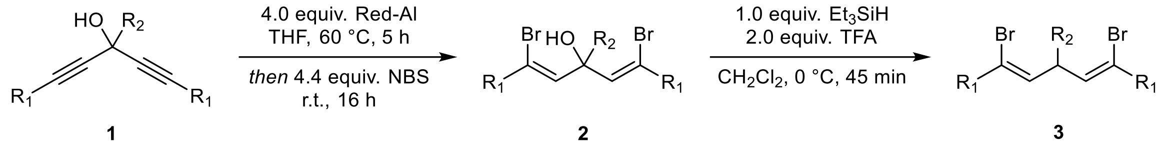

General procedure C for synthesis of dibromo precursors from dialkyne alcohols

In a round-bottom flask, a 0.2 M solution of dialkyne alcohol **1** in THF was cooled using an ice bath. Slowly, 4.0 equiv. of sodium bis(2-methoxyethoxy)aluminum hydride (Red-Al® 70 % wt. in toluene) was added to the reaction mixture. After one hour, the flask was removed from the ice bath and heated to 60 °C for 5 hours. The reaction was then cooled to –78 °C using a dry ice/acetone bath, and 4.4 equiv. *N*-bromosuccinimide was added in small portions. The reaction was allowed to warm up to room temperature while stirring overnight. Upon workup, an equal volume of aqueous $Na_2S_2O_3$ (1 M) and aqueous Rochelle salt (1 M) were added and the mixture was allowed to stir for 1 hour. The organic layer was separated, and the aqueous layer was extracted with diethyl ether. The combined organic fractions were washed with aqueous $Na_2S_2O_3$ (1 M), saturated aqueous $NaHCO_3$, and brine. Removal of the solvent *in vacuo* provided the dark brown oil, which was filtered through a plug of neutral alumina in 1/1 hexanes/ethyl acetate to provide the crude dibromo alcohol **2**.

A 0.5 M solution of **2** in CH_2Cl_2 was cooled to 0 °C using an ice bath. Subsequently, 1.0 equiv. triethylsilane and 2.0 equiv. trifluoroacetic acid were added and the reaction mixture was stirred for 45 minutes. An equal volume of saturated aqueous $NaHCO_3$ was added, and the mixture was stirred vigorously for 15 minutes to quench the remaining acid. The organic layer was separated, and the aqueous layer was extracted twice more with CH_2Cl_2. The combined organic fractions were washed with brine, dried over anhydrous Na_2SO_4, and the solvents were removed *in vacuo*. The crude residue was purified by flash column chromatography as noted. Yields for the dibromide precursor **3** are reported over two steps. These compounds were stored in a –40 °C freezer inside a glovebox until use.

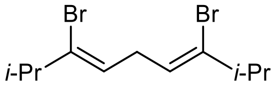

(3*Z*,6*Z*)-3,7-dibromo-2,8-dimethylnona-3,6-diene (**3a**)

Synthesized according to general procedure C from **1a** (2.46 g, 15.0 mmol). Title compound was obtained as a pale-yellow oil in 28% yield (1.30 g, 4.19 mmol) over two-steps after purification by silica flash column chromatography. $\boldsymbol{R_f}$ = 0.90 (100% hexanes).

^{1}H NMR (400 MHz, $CDCl_3$) δ (ppm): 5.67 (t, *J* = 6.8 Hz, 2H), 3.03 (t, *J* = 6.8 Hz, 2H), 2.55 (h, *J* = 6.7 Hz, 2H), 1.11 (d, *J* = 6.4 Hz, 12H)

^{13}C{^{1}H} NMR (101 MHz, $CDCl_3$) δ (ppm): 137.8, 122.9, 39.1, 33.6, 22.1

HRMS (EI-TOF) calculated 307.9775, 309.9755, 311.9734 for $C_{11}H_{18}Br_2$ [M], found 307.9770, 309.9749, 311.9726. Average error 2.1 ppm.

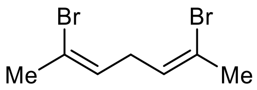

(2*Z*,5*Z*)-2,6-dibromohepta-2,5-diene (**3b**)

Synthesized according to general procedure C from **1b** (1.21 g, 11.2 mmol). Title compound was obtained as a pale-yellow oil in 30% yield (859 mg, 3.38 mmol) over two-steps after purification by silica flash column chromatography. R_f = 0.63 (100% hexanes).

^{1}H NMR (400 MHz, $CDCl_3$) δ (ppm): 5.62 (td, *J* = 6.8, 1.2 Hz, 2H), 2.98 (tt, *J* = 7.0, 1.2 Hz, 2H), 2.28 (d, *J* = 1.20 Hz, 6H)

^{13}C{^{1}H} NMR (101 MHz, $CDCl_3$) δ (ppm): 125.6, 123.7, 34.3, 28.8

HRMS (EI-TOF) calculated 251.9149, 253.9129, 255.9108 for $C_7H_{10}Br_2$ [M], found 251.9145, 253.9131, 255.9104. Average error 1.4 ppm.

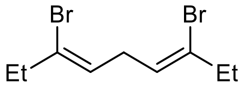

(3*Z*,6*Z*)-3,7-dibromonona-3,6-diene (**3c**)

Synthesized according to general procedure C from **1c** (1.56 g, 11.5 mmol). Title compound was obtained as a pale-yellow oil in 27% yield (888 mg, 3.14 mmol) over two-steps after purification by silica flash column chromatography. R_f = 0.80 (100% hexanes).

^{1}H NMR (400 MHz, $CDCl_3$) δ (ppm): 5.64 (t, *J* = 6.8 Hz, 2H), 3.02 (t, *J* = 6.8 Hz, 2H), 2.46 (q, *J* = 7.3 Hz, 4H), 1.12 (t, *J* = 7.3 Hz, 6H)

^{13}C{^{1}H} NMR (101 MHz, $CDCl_3$) δ (ppm): 131.4, 124.1, 35.1, 33.9, 13.6

HRMS (EI-TOF) calculated 279.9462, 281.9442, 283.9421 for $C_9H_{14}Br_2$ [M], found 279.9468, 281.9454, 283.9429. Average error 3.0 ppm.

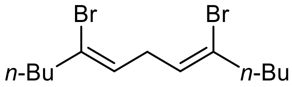

(5*Z*,8*Z*)-5,9-dibromotrideca-5,8-diene (**3d**)

Synthesized according to general procedure C from **1d** (2.38 g, 12.4 mmol). Title compound was obtained as a pale-yellow oil in 48% yield (2.00 g, 5.94 mmol) over two-steps after purification by silica flash column chromatography. R_f = 0.90 (100% hexanes).

^{1}H NMR (400 MHz, $CDCl_3$) δ (ppm): 5.63 (t, *J* = 6.9 Hz, 2H), 3.01 (t, *J* = 6.9 Hz, 2H), 2.42 (t, *J* = 7.4 Hz, 4H), 1.57 – 1.49 (m, 4H), 1.36 – 1.25 (m, 4H), 0.91 (t, *J* = 7.3 Hz, 6H).

^{13}C{^{1}H} NMR (101 MHz, $CDCl_3$) δ (ppm): 129.9, 125.0, 41.3, 34.0, 30.4, 21.7, 14.0.

HRMS (EI-TOF) calculated 336.0088, 338.0068, 340.0047 for $C_{13}H_{22}Br_2$ [M], found 336.0109, 338.0095, 340.0068. Average error 6.8 ppm.

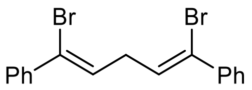

(1*Z*,4*Z*)-1,5-dibromo-1,5-diphenylpenta-1,4-diene (**3e**)

Synthesized according to general procedure C from **1e** (2.83 g, 12.2 mmol). Title compound was obtained as a pale-yellow oil in 17% yield (784 mg, 2.07 mmol) over two-steps after purification by silica flash column chromatography. R_f = 0.25 (100% hexanes).

^{1}H NMR (400 MHz, $CDCl_3$) δ (ppm): 7.58 – 7.54 (m, 4H), 7.39 – 7.29 (m, 6H), 6.32 (t, *J* = 6.9 Hz, 2H), 3.46 (t, *J* = 7.0 Hz, 2H)

^{13}C{^{1}H} NMR (101 MHz, $CDCl_3$) δ (ppm): 139.7, 128.8, 128.4, 127.7, 127.5, 127.3, 36.1.

HRMS (EI-TOF) calculated 375.9462, 377.9442, 379.9421 for $C_{17}H_{14}Br_2$ [M], found 375.9494, 377.9488, 379.9454. Average error 9.7 ppm.

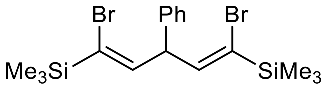

((1*Z*,4*Z*)-1,5-dibromo-3-phenylpenta-1,4-diene-1,5-diyl)bis(trimethylsilane) (**3f-TMS**)

Synthesized according to general procedure C from **1f-TMS** (3.60 g, 12.0 mmol). Title compound was obtained as a pale-yellow oil in 28% yield (1.48 g, 3.31 mmol) over two-steps after purification by silica flash column chromatography. R_f = 0.47 (100% hexanes).

^{1}H NMR (400 MHz, $CDCl_3$) δ (ppm): 7.35 – 7.22 (m, 5H), 6.38 (d, *J* = 8.6 Hz, 2H), 5.39 (t, *J* = 8.6 Hz, 1H), 0.22 (s, 18H).

^{13}C{^{1}H} NMR (101 MHz, $CDCl_3$) δ (ppm): 140.6, 133.1, 128.9, 127.6, 126.8, 126.4, 51.4, -1.7.

HRMS (EI-TOF) calculated 443.9940, 445.9919, 447.9899 for $C_{17}H_{26}Br_2Si_2$ [M], found 443.9905, 445.9912, 447.9830. Average error 8.3 ppm.

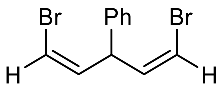

((1*Z*,4*Z*)-1,5-dibromopenta-1,4-dien-3-yl)benzene (**3f**)

In a 100 mL round-bottom flask at 0 °C, **3f-TMS** (1.48 g, 3.31 mmol) was dissolved in 20 mL diethyl ether, and tetrabutyl ammonium fluoride (1 M in THF, 8.3 mL, 8.3 mmol) was added dropwise. After stirring for one hour, 20 mL brine was added, and the mixture was allowed to warm to room temperature. The layers were separated, and the aqueous layer was extracted diethyl ether (3 x 20 mL). The combined organic layers were washed with brine, dried over anhydrous Na_2SO_4, filtered, and concentrated. Title compound was obtained as a pale-yellow oil in 20% yield (200 mg, 0.66 mmol) after purification by silica flash column chromatography. $\boldsymbol{R_f}$ = 0.43 (100% hexanes).

^{1}H NMR (400 MHz, $CDCl_3$) δ (ppm): 7.42 – 7.21 (m, 5H), 6.39 (d, *J* = 7.0 Hz, 2H), 6.30 (dd, *J* = 9.1, 7.0 Hz, 2H), 5.17 (t, *J* = 9.1 Hz, 1H).

^{13}C{^{1}H} NMR (101 MHz, $CDCl_3$) δ (ppm): 140.1, 134.3, 128.9, 127.4, 127.1, 109.6, 46.4.

HRMS (EI-TOF) calculated 299.9149, 301.9129, 303.9108 for $C_{11}H_{10}Br_2$ [M], found 299.9148, 301.9117, 303.9100. Average error 2.4 ppm.

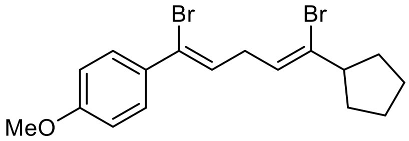

1-((1*Z*,4*Z*)-1,5-dibromo-5-cyclopentylpenta-1,4-dien-1-yl)-4-methoxybenzene (**3g**)

Synthesized according to general procedure C from **1g** (1.31 g, 5.14 mmol). Title compound was obtained as a pale-yellow oil in 14% yield (285 mg, 0.712 mmol) over two-steps after purification by silica flash column chromatography. $\boldsymbol{R_f}$ = 0.43 (20% CH_2Cl_2 in hexanes).

^{1}H NMR (400 MHz, $CDCl_3$) δ (ppm): 7.47 (d, *J* = 8.9 Hz, 2H), 6.85 (d, *J* = 8.9 Hz, 2H), 6.09 (t, *J* = 6.9 Hz, 1H), 5.79 (t, *J* = 6.8 Hz, 1H), 3.82 (s, 3H), 3.22 (t, *J* = 6.9 Hz, 2H), 2.75 (t, *J* = 8.3 Hz, 1H), 1.96 – 1.46 (m, 8H).

^{13}C{^{1}H} NMR (101 MHz, $CDCl_3$) δ (ppm): 159.9, 139.2, 135.5, 132.6, 129.0, 126.7, 123.0, 113.7, 55.5, 50.3, 35.0, 32.3, 25.4.

HRMS (ESI-TOF) calculated 398.9954, 400.9933, 402.9913 for $C_{17}H_{21}Br_2O^+$ [M+H]$^+$, found 398.9945, 400.9919, 402.9888. Average error 4.0 ppm.

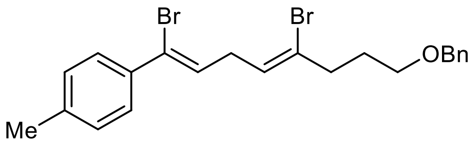

1-((1*Z*,4*Z*)-8-(benzyloxy)-1,5-dibromoocta-1,4-dien-1-yl)-4-methylbenzene (**3h**)

Synthesized according to general procedure C from **1h** (2.34 g, 7.34 mmol). Title compound was obtained as a pale-yellow oil in 35% yield (1.21 g, 2.60 mmol) over two-steps after purification by silica flash column chromatography. R_f = 0.35 (5% Et_2O in hexanes).

^{1}H NMR (400 MHz, $CDCl_3$) δ (ppm): 7.41 (d, *J* = 8.2 Hz, 2H), 7.37 – 7.31 (m, 5H), 7.14 (d, *J* = 8.3 Hz, 2H), 6.13 (t, *J* = 6.9 Hz, 1H), 5.75 (t, *J* = 6.9 Hz, 1H), 4.49 (s, 2H), 3.48 (t, *J* = 6.1 Hz, 2H), 3.20 (t, *J* = 6.9 Hz, 2H), 2.58 (t, *J* = 7.3 Hz, 2H), 2.36 (s, 3H), 1.94 – 1.84 (m, 2H).

^{13}C{^{1}H} NMR (101 MHz, $CDCl_3$) δ (ppm): 138.7, 138.6, 137.1, 129.5, 129.1, 128.5, 127.8, 127.7, 127.6, 127.2, 126.9, 125.4, 73.1, 68.7, 38.3, 35.0, 28.4, 21.3.

HRMS (ESI-TOF) calculated 463.0267, 465.0246, 467.0226 for $C_{22}H_{25}Br_2O^+$ $[M+H]^+$, found 463.0150, 465.0190, 467.0192. Average error 14.8 ppm.

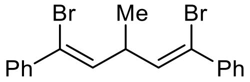

((1*Z*,4*Z*)-1,5-dibromo-3-methylpenta-1,4-diene-1,5-diyl)dibenzene (**3i**)

Synthesized according to general procedure C from **1i** (2.86 g, 11.6 mmol). Title compound was obtained as a pale-yellow oil in 22% yield (1.00 g, 2.55 mmol) over two-steps after purification by silica flash column chromatography. R_f = 0.30 (100% hexanes).

^{1}H NMR (400 MHz, $CDCl_3$) δ (ppm): 7.59 – 7.49 (m, 4H), 7.40 – 7.25 (m, 6H), 6.18 (d, *J* = 8.5 Hz, 2H), 4.06 (tq, *J* = 8.6, 6.9 Hz, 1H), 1.31 (d, *J* = 6.9 Hz, 3H).

^{13}C{^{1}H} NMR (101 MHz, $CDCl_3$) δ (ppm): 139.9, 133.6, 128.7, 128.4, 127.8, 125.5, 41.4, 19.6

HRMS (EI-TOF) calculated 389.9619, 391.9598, 393.9578 for $C_{18}H_{16}Br_2$ [M], found 389.9629, 391.9617, 393.9593. Average error 3.8 ppm.

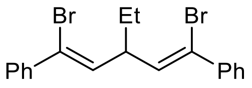

((1*Z*,4*Z*)-1,5-dibromo-3-ethylpenta-1,4-diene-1,5-diyl)dibenzene (**3j**)

Synthesized according to general procedure C from **1j** (3.30 g, 12.7 mmol). After silica flash column chromatography (***R**$_f$* = 0.33 (100% hexanes)), the title compound was obtained in an inseparable mixture with impurities as a pale-yellow oil. Isolation of this pure compound was not successful; however, the desired compound can be seen via ^{1}H NMR and HRMS. The product as obtained was carried forward without further purification. It is used in excess in the subsequent phenol synthesis.

HRMS (EI-TOF) calculated 403.9775, 405.9755, 407.9734 for $C_{19}H_{18}Br_2$ [M], found 403.9791, 405.9781, 407.9756. Average error 5.2 ppm.

^{1}H NMR of mixture containing ((1*Z*,4*Z*)-1,5-dibromo-3-ethylpenta-1,4-diene-1,5-diyl)dibenzene (**3j**) in $CDCl_3$, 400 MHz

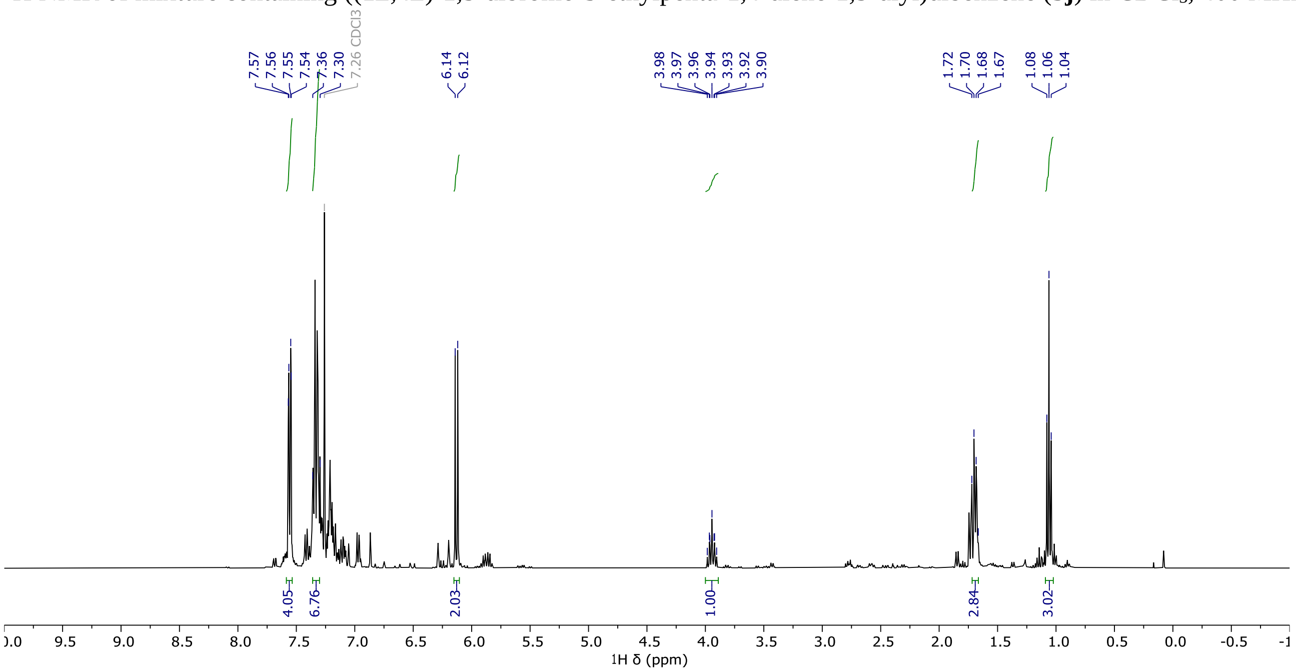

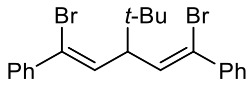

((1*Z*,4*Z*)-1,5-dibromo-3-(*tert*-butyl)penta-1,4-diene-1,5-diyl)dibenzene (**3k**)

Synthesized according to general procedure C from **1k** (1.36 g, 4.7 mmol). Title compound was obtained as a pale-yellow oil in 35% yield (717 mg, 1.65 mmol) over two-steps after purification by silica flash column chromatography. $\boldsymbol{R_f}$ = 0.33 (100% hexanes).

^{1}H NMR (400 MHz, $CDCl_3$) δ (ppm): 7.58 – 7.51 (m, 4H), 7.39 – 7.28 (m, 6H), 6.16 (d, *J* = 9.8 Hz, 2H), 3.97 (t, *J* = 9.8 Hz, 1H), 1.08 (s, 9H).

^{13}C{^{1}H} NMR (101 MHz, $CDCl_3$) δ (ppm): 140.4, 129.8, 128.5, 128.2, 127.8, 126.9, 56.1, 36.3, 27.7.

HRMS (EI-TOF) calculated 353.0905, 355.0884 for $C_{21}H_{22}Br\cdot$ [M-Br]·, found 353.0927, 355.0908. Average error 6.5 ppm.

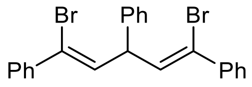

((1*Z*,4*Z*)-1,5-dibromopenta-1,4-diene-1,3,5-triyl)tribenzene (**3l**)

Synthesized according to general procedure C from **1l** (1.39 g, 4.49 mmol). Title compound was obtained as an off-white solid in 52% yield (1.06 g, 2.34 mmol) over two-steps after purification by silica flash column chromatography. $\boldsymbol{R_f}$ = 0.50 (10% EtOAc in hexanes).

^{1}H NMR (400 MHz, $CDCl_3$) δ (ppm): 7.62 – 7.57 (m, 4H), 7.43 – 7.25 (m, 11H), 6.46 (d, *J* = 9.0 Hz, 2H), 5.35 (t, *J* = 8.9 Hz, 1H).

^{13}C{^{1}H} NMR (101 MHz, $CDCl_3$) δ (ppm): 141.0, 139.7, 131.1, 129.0, 128.9, 128.5, 127.9, 127.6, 127.2, 127.1, 51.6.

HRMS (EI-TOF) calculated 373.0592, 375.0571 for $C_{23}H_{18}Br\cdot$ [M-Br]·, found 373.0600, 375.0582. Average error 2.5 ppm.

Synthesis of Dialkyne Alcohols

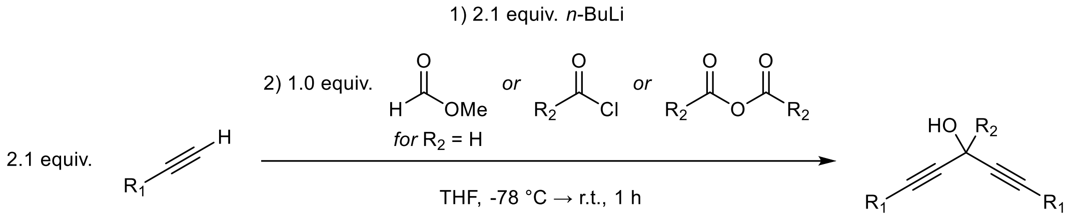

1

General procedure D for the synthesis of symmetric dialkyne alcohols

Symmetric dialkyne alcohols were prepared according to modified literature procedures.[6] A stirred solution of alkyne (25 mmol) in 50 mL THF at –78 °C was treated dropwise with *n*-butyllithium (2.5 M in hexanes, 10 mL, 25 mmol). Afterwards, 11.9 mmol of methyl formate, acyl chloride, or acid anhydride was added dropwise. The solution was allowed to warm to room temperature and stirred for 1 hour, followed by the addition of 50 mL aqueous sat. ammonium chloride. The layers were separated, and the aqueous layer was extracted diethyl ether (3 x 50 mL). The combined organic layers were washed with brine, dried over anhydrous Na_2SO_4, filtered, and concentrated. In most cases, the crude organic extract was brought forward without further purification.

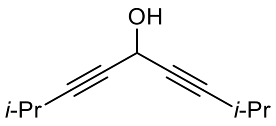

2,8-dimethylnona-3,6-diyn-5-ol (**1a**)

Synthesized according to general procedure D from 3-methylbutyne (2.55 g, 37.5 mmol) and methyl formate (1.05 g, 17.4 mmol). The title compound was isolated as an off-white solid in 98% yield (2.80 g, 17.1 mmol) after extraction and was brought forward without further purification.

^{1}H NMR (400 MHz, $CDCl_3$) δ (ppm): 4.34 (dt, *J* = 6.5, 3.3 Hz, 1H), 2.59 (hd, *J* = 6.8, 1.9 Hz, 2H), 2.06 (s, 1H), 1.18 (d, *J* = 6.7 Hz, 12H).

Spectroscopic data agree with those in the literature.[6]

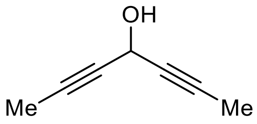

hepta-2,5-diyn-4-ol (**1b**)

Synthesized according to a modified general procedure D. For 10 minutes, propyne was sparged into a solution of *n*-butyllithium (2.5 M in hexanes, 10 mL, 25 mmol) in 50 mL THF at –78 °C. Afterwards, methyl formate (742 mg, 12.3 mmol) was added dropwise. The solution was allowed to warm to room temperature and stirred for 1 hour, followed by the addition of 50 mL aqueous sat. ammonium chloride. The layers were separated, and the aqueous layer was extracted diethyl ether (3 x 50 mL). The combined organic layers were washed with brine, dried over anhydrous Na_2SO_4, filtered, and concentrated. The title compound was isolated as an off-white solid in 91% yield (1.21 g, 11.2 mmol) after extraction and was brought forward without further purification.

^{1}H NMR (400 MHz, $CDCl_3$) δ (ppm): 5.06 (dp, *J* = 6.8, 2.2 Hz, 1H), 2.03 (d, *J* = 7.0 Hz, 1H), 1.88 (d, *J* = 2.2 Hz, 6H).

Spectroscopic data agree with those in the literature.[7]

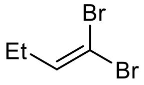

1,1-dibromobut-1-ene (**S-1**) was synthesized from propionaldehyde according to literature procedures.[8]

^{1}H NMR (400 MHz, $CDCl_3$) δ (ppm): 6.39 (t, *J* = 7.2 Hz, 1H), 2.11 (p, *J* = 7.5 Hz, 2H), 1.03 (t, *J* = 7.6 Hz, 3H).

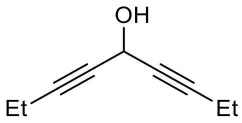

nona-3,6-diyn-5-ol (**1c**)

To a solution of dibromoalkene **S-1** (4.54 g, 21.2 mmol) in 40 mL THF, *n*-butyllithium (2.5 M in hexanes, 17.4 mL, 43.4 mmol) was added dropwise at –78 °C. The solution was allowed to warm to 0 °C in an ice bath and stirred for 1 hour. Then, methyl formate (585 mg, 9.63 mmol) was added dropwise at 0 °C. The solution was allowed to warm to room temperature and stirred for 1 hour, followed by the addition of 50 mL aqueous sat. ammonium chloride. The layers were separated, and the aqueous layer was extracted diethyl ether (3 x 50 mL). The combined organic layers were washed with brine, dried over anhydrous Na_2SO_4, filtered, and concentrated. The title compound was isolated as a yellow oil in 54% yield (1.56 g, 11.45 mmol) after extraction and was brought forward without further purification.

^{1}H NMR (400 MHz, $CDCl_3$) δ (ppm): δ 5.09 (dt, *J* = 4.2, 2.0 Hz, 1H), 2.24 (qd, *J* = 7.5, 2.0 Hz, 4H), 2.09 (br s, 1H), 1.15 (t, *J* = 7.5 Hz, 6H).

^{13}C{^{1}H} NMR (101 MHz, $CDCl_3$) δ (ppm): 86.5, 77.5, 52.7, 13.6, 12.6.

HRMS (ESI-TOF) calculated 137.0961 for $C_9H_{13}O^+$ $[M+H]^+$, found 137.0959. Error 1.4 ppm.

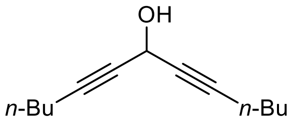

trideca-5,8-diyn-7-ol (**1d**)

Synthesized according to general procedure D from 1-hexyne (2.05 g, 25.0 mmol) and methyl formate (744 mg, 12.4 mmol). The title compound was isolated as a yellow oil in quantitative yield (2.38 g, 12.4 mmol) after extraction and was brought forward without further purification.

^{1}H NMR (400 MHz, $CDCl_3$) δ (ppm): 5.09 (dt, *J* = 6.8, 2.1 Hz, 1H), 2.23 (td, *J* = 7.0, 2.0 Hz, 4H), 2.05 (d, *J* = 6.7 Hz, 1H), 1.54 – 1.35 (m, 8H), 0.91 (t, *J* = 7.2 Hz, 6H).

Spectroscopic data agree with those in the literature.[9]

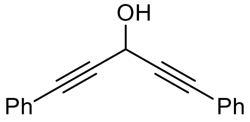

1,5-diphenylpenta-1,4-diyn-3-ol (**1e**)

Synthesized according to general procedure D from phenylacetylene (2.55 g, 25.0 mmol) and methyl formate (744 mg, 12.4 mmol). The title compound was isolated as an off-white solid in 98% yield (2.83 g, 12.2 mmol) after extraction and was brought forward without further purification.

^{1}H NMR (400 MHz, $CDCl_3$) δ (ppm): 7.53 – 7.47 (m, 4H), 7.37 – 7.29 (m, 6H), 5.58 (d, *J* = 7.5 Hz, 1H), 2.36 (d, *J* = 7.7 Hz, 1H).

Spectroscopic data agree with those in the literature.[9]

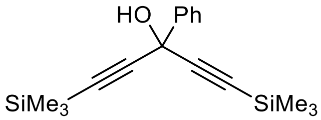

3-phenyl-1,5-bis(trimethylsilyl)penta-1,4-diyn-3-ol (**1f-TMS**)

Synthesized according to general procedure D from trimethylsilylacetylene (2.46 g, 25.0 mmol) and benzoyl chloride (1.67 g, 11.9 mmol). The title compound was isolated as an off-white solid in 96% yield (3.42 g, 11.4 mmol) after extraction and was brought forward without further purification.

^{1}H NMR (400 MHz, $CDCl_3$) δ (ppm): 7.83 – 7.76 (m, 2H), 7.44 – 7.29 (m, 3H), 2.80 (s, 1H), 0.21 (s, 18H).

^{13}C{^{1}H} NMR (101 MHz, $CDCl_3$) δ (ppm): 141.6, 128.8, 128.5, 126.2, 104.6, 90.3, 65.7, -0.2.

HRMS (ESI-TOF) calculated 301.1438 for $C_{17}H_{25}OSi_2^+$ $[M+H]^+$, found 301.1442. Error 1.2 ppm.

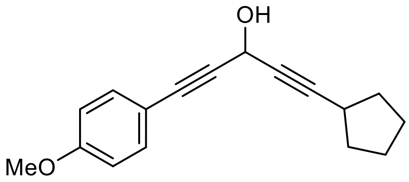

1-cyclopentyl-5-(4-methoxyphenyl)penta-1,4-diyn-3-ol (**1g**)

In an oven-dried 50 mL pear-shaped flask, a solution of ((4-methoxyphenyl)ethynyl)lithium was prepared by dissolving 1-ethynyl-4-methoxybenzene (1.06 g, 8.0 mmol) in 15 mL THF and adding *n*-butyllithium (2.5 M in hexanes, 3.2 mL, 8.0 mmol) dropwise at –78 °C. In a separate oven-dried 50 mL pear-shaped flask, a solution of (cyclopentylethynyl)lithium was similarly prepared by dissolving ethynylcyclopentane (829 mg, 8.8 mmol) in 15 mL THF and adding *n*-butyllithium (2.5 M in hexanes, 3.6 mL, 8.8 mmol) dropwise at –78 °C.

To an oven-dried 250 mL Schlenk flask equipped with a magnetic stir bar, methyl formate (480 mg, 8.0 mmol) was dissolved in 15 mL THF and cooled to –78 °C. Each of the prepared lithiate solutions was then added via cannula transfer over the course of 10 minutes: first ((4-methoxyphenyl)ethynyl)lithium, and then (cyclopentylethynyl)lithium. Afterwards, the reaction mixture was allowed to warm to room temperature and stirred for 1 hour, followed by the addition of 50 mL aqueous sat. ammonium chloride. The layers were separated, and the aqueous layer was extracted diethyl ether (3 x 50 mL). The combined organic layers were washed with brine, dried over anhydrous Na_2SO_4, filtered, and concentrated. Title compound was obtained as a yellow oil in 64% yield (1.31 g, 5.14 mmol) after purification by silica flash column chromatography. $\boldsymbol{R_f}$ = 0.13 (10% EtOAc in hexanes).

^{1}H NMR (400 MHz, $CDCl_3$) δ (ppm): 7.40 (d, *J* = 9.0 Hz, 2H), 6.84 (d, *J* = 9.0 Hz, 2H), 5.33 (d, *J* = 4.9 Hz, 1H), 3.81 (s, 3H), 2.67 (pd, *J* = 7.6, 2.0 Hz, 1H), 2.28 – 2.16 (m, 1H), 1.99 – 1.88 (m, 2H), 1.82 – 1.49 (m, 6H).

^{13}C{^{1}H} NMR (101 MHz, $CDCl_3$) δ (ppm): 160.1, 135.6, 133.5, 114.4, 114.1, 89.9, 85.6, 84.0, 55.4, 53.2, 33.7, 30.2, 25.2.

HRMS (ESI-TOF) calculated 255.1380 for $C_{17}H_{19}O_2^+$ $[M+H]^+$, found 255.1354. Error 10.0 ppm.

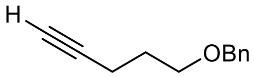

((pent-4-yn-1-yloxy)methyl)benzene (**S-2**) was synthesized according to literature procedures.[10]

^{1}H NMR (400 MHz, $CDCl_3$) δ (ppm): 7.37 – 7.27 (m, 5H), 4.52 (s, 2H), 3.58 (t, *J* = 6.2 Hz, 2H), 2.33 (td, *J* = 7.1, 2.7 Hz, 2H), 1.94 (t, *J* = 2.6 Hz, 1H), 1.88 – 1.80 (m, 2H).

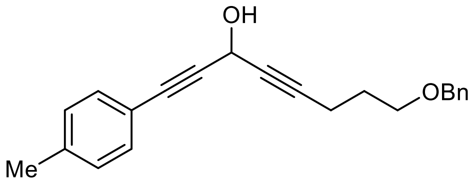

8-(benzyloxy)-1-(p-tolyl)octa-1,4-diyn-3-ol (**1h**)

Synthesized analogously to **1g** from 1-ethynyl-4-methylbenzene (1.21 g, 10.4 mmol), **S-2** (1.97 g, 11.3 mmol), and methyl formate (625 mg, 10.4 mmol). Title compound was obtained as a yellow oil in 70% yield (2.33 g, 7.30 mmol) after purification by silica flash column chromatography. R_f = 0.10 (10% EtOAc in hexanes).

^{1}H NMR (400 MHz, $CDCl_3$) δ (ppm): 7.39 – 7.27 (m, 7H), 7.12 (d, *J* = 7.8 Hz, 2H), 5.30 (dt, *J* = 7.5, 2.1 Hz, 1H), 4.52 (s, 2H), 3.58 (t, *J* = 6.2 Hz, 2H), 2.40 (td, *J* = 7.1, 2.0 Hz, 2H), 2.35 (s, 3H), 2.20 – 2.14 (m, 1H), 1.85 (p, *J* = 6.7 Hz, 2H).

^{13}C{^{1}H} NMR (101 MHz, $CDCl_3$) δ (ppm): 139.1, 138.6, 131.9, 129.2, 128.5, 127.8, 127.7, 119.1, 86.1, 85.2, 84.3, 78.0, 73.1, 68.8, 53.1, 28.6, 21.7, 15.8.

HRMS (ESI-TOF) calculated 319.1693 for $C_{22}H_{23}O_2^+$ $[M+H]^+$, found 319.1680. Error 3.9 ppm.

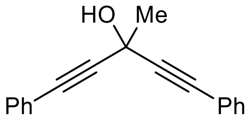

3-methyl-1,5-diphenylpenta-1,4-diyn-3-ol (**1i**)

Synthesized according to general procedure D from phenylacetylene (2.55 g, 25.0 mmol) and acetic anhydride (1.20 g, 11.75 mmol). The title compound was isolated as an off-white solid in 99% yield (2.86 g, 11.6 mmol) after extraction and was brought forward without further purification.

^{1}H NMR (400 MHz, $CDCl_3$) δ (ppm): 7.51 – 7.46 (m, 4H), 7.36 – 7.29 (m, 6H), 2.65 (s, 1H), 1.96 (s, 3H).

Spectroscopic data agree with those in the literature.[11]

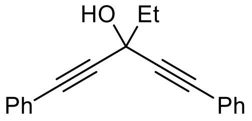

3-ethyl-1,5-diphenylpenta-1,4-diyn-3-ol (**1j**)

Synthesized according to general procedure D from phenylacetylene (2.55 g, 25.0 mmol) and propionic anhydride (1.53 g, 11.75 mmol). The title compound was isolated as an off-white solid in 99% yield (3.03 g, 11.6 mmol) after extraction and was brought forward without further purification.

^{1}H NMR (400 MHz, $CDCl_3$) δ (ppm): 7.54 – 7.43 (m, 4H), 7.39 – 7.26 (m, 6H), 2.63 (s, 1H), 2.13 (q, *J* = 7.4 Hz, 2H), 1.25 (t, *J* = 7.4 Hz, 3H).

Spectroscopic data agree with those in the literature.[11]

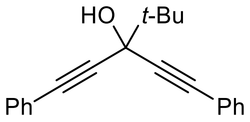

3-(tert-butyl)-1,5-diphenylpenta-1,4-diyn-3-ol (**1k**)

Synthesized according to general procedure D from phenylacetylene (1.02 g, 10.0 mmol) and pivaloyl chloride (568 mg, 4.71 mmol). The title compound was isolated as an off-white solid in quantitative yield (1.36 g, 4.70 mmol) after extraction and was brought forward without further purification.

^{1}H NMR (400 MHz, $CDCl_3$) δ (ppm): 7.51 – 7.47 (m, 4H), 7.36 – 7.29 (m, 6H), 2.51 (s, 1H), 1.27 (s, 9H).

Spectroscopic data agree with those in the literature.[11]

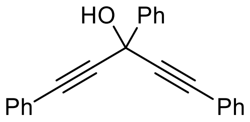

1,3,5-triphenylpenta-1,4-diyn-3-ol (**1l**)

Synthesized according to general procedure D from phenylacetylene (1.02 g, 10.0 mmol) and benzyl chloride (683 mg, 4.86 mmol). The title compound was isolated as an off-white solid in 92% yield (1.39 g, 4.49 mmol) after extraction and was brought forward without further purification.

^{1}H NMR (400 MHz, $CDCl_3$) δ (ppm): 7.98 – 7.92 (m, 2H), 7.55 – 7.50 (m, 4H), 7.47 – 7.42 (m, 2H), 7.42 – 7.30 (m, 7H), 3.03 (s, 1H).

^{13}C{^{1}H} NMR (101 MHz, $CDCl_3$) δ (ppm): 142.0, 131.9, 128.9, 128.8, 128.5, 128.3, 126.0, 122.0, 89.0, 85.2, 66.0.

HRMS (ESI-TOF) calculated 309.1274 for $C_{23}H_{17}O^+$ $[M+H]^+$, found 309.1271. Error 0.9 ppm.

Carbon-13 T_1 Measurement

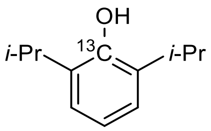

4a-*1*-^{13}C

A sample of 10 mg **4a-*1*-^{13}C** in 0.50 mL $CDCl_3$ was prepared. Using a Bruker spectrometer, a ^{13}C T_1 inversion-recovery experiment was programmed in TOPSPIN using the following parameters:

Pulse program:	t1irig
Number of scans:	4
D1 delay:	180 sec
Variable delays:	0.001, 0.250, 1, 2, 4, 8, 16, 32, 48, 64, 96, 128 sec
Field strength:	126 MHz

Scans were processed in MestReNova with uniform processing parameters. The integral of the labeled carbon was plotted against the variable time delay and the exponential curve was fit to the following equation, where I is the integral value, τ is the variable time delay, and T_1 is the calculated longitudinal relaxation time.

$$I(\tau) = I_\infty\left[1 - 2\exp\left(\frac{-\tau}{T_1}\right)\right]$$

The calculated value of T_1 was found to be 29.4 s for the labeled carbon of **4a-*1*-^{13}C**.

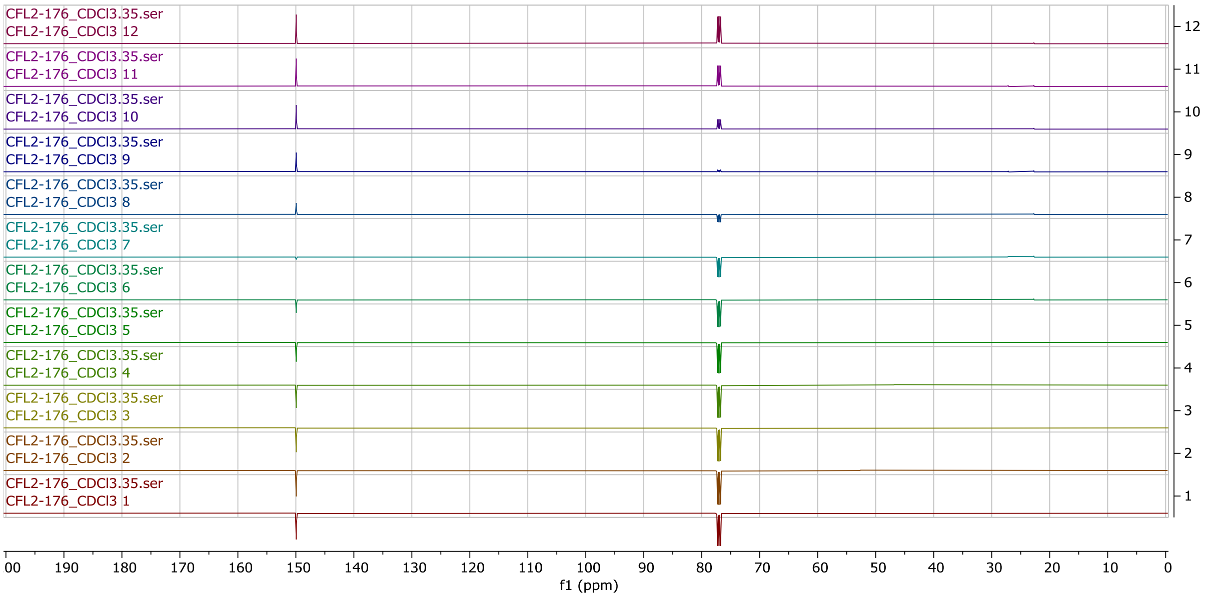

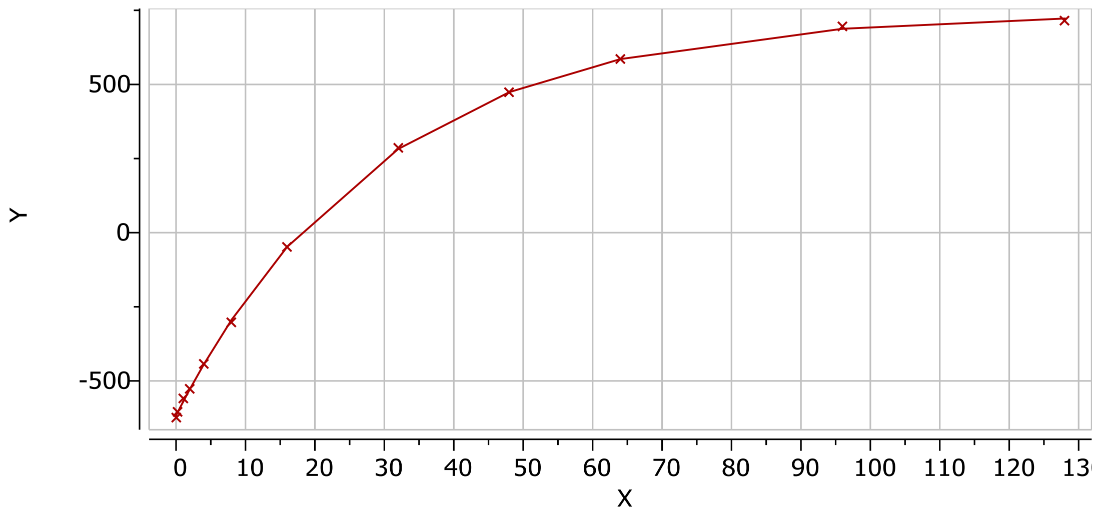

#	X(I)	Y(X)	Y'(X)
Model	ARR_DATA(I)	Integral(150.051,149.822)	B+F*exp(-x*G)<br>B= 739.852<br>F= -1356.2<br>G= 0.0339584
1	0.001	-624.075	-616.302
2	0.250	-604.448	-604.884
3	1.000	-559.380	-571.067
4	2.000	-526.151	-527.298
5	4.000	-443.355	-444.095
6	8.000	-302.298	-293.719
7	16.000	-48.070	-47.841
8	32.000	285.347	282.353
9	48.000	473.085	474.133
10	64.000	585.465	585.520
11	96.000	695.214	687.790
12	128.000	715.542	722.289

$$29.4\ \mathrm{s} = \frac{1}{0.0339584\ \mathrm{s}^{-1}}$$

References

(1) Pan, W.; Li, C.; Zhu, H.; Li, F.; Li, T.; Zhao, W. A Mild and Practical Method for Deprotection of Aryl Methyl/Benzyl/Allyl Ethers with HPPh2 and TBuOK. *Org. Biomol. Chem.* **2021**, *19* (35), 7633–7640. https://doi.org/10.1039/D1OB01286J.

(2) Wu, Z.; Jiang, H.; Zhang, Y. Pd-Catalyzed Cross-Electrophile Coupling/C–H Alkylation Reaction Enabled by a Mediator Generated via C(Sp3)–H Activation. *Chem. Sci.* **2021**, *12* (24), 8531–8536. https://doi.org/10.1039/D1SC01731D.

(3) Wu, L.-J.; Song, R.-J.; Luo, S.; Li, J.-H. Palladium-Catalyzed Reductive [5+1] Cycloaddition of 3-Acetoxy-1,4-Enynes with CO: Access to Phenols Enabled by Hydrosilanes. *Angew. Chem. Int. Ed.* **2018**, *57* (40), 13308–13312. https://doi.org/10.1002/anie.201808388.

(4) Hartshorn, M.; Martyn, R.; Robinson, W.; Sutton, K.; Vaughan, J.; White, J. Reactions of 4-Methyl-2,6-Diphenylphenol and 4-Nitro-2,6-Diphenylphenol with Nitrogen Dioxide. *Aust. J. Chem.* **1985**, *38* (11), 1613–1630.

(5) Lim, Y. N.; Lee, C.; Jang, H.-Y. Metal-Free Synthesis of Cyclic and Acyclic Carbonates from CO2 and Alcohols. *Eur. J. Org. Chem.* **2014**, *2014* (9), 1823–1826. https://doi.org/10.1002/ejoc.201400031.

(6) Parker, K. A.; Katsoulis, I. A. A Strategy for Exploiting the Pseudosymmetry of the C1−C13 Stretch of Discodermolide. *Org. Lett.* **2004**, *6* (9), 1413–1416. https://doi.org/10.1021/ol049735p.

(7) Carmichael, R. A.; Chalifoux, W. A. Multicomponent Double Diels–Alder/Nazarov Tandem Cyclization of Symmetric Cross-Conjugated Diynones to Generate [6-5-6] Tricyclic Products. *Chem. – Eur. J.* **2016**, *22* (26), 8781–8785. https://doi.org/10.1002/chem.201601850.

(8) Traboulsi, I.; Dange, N. S.; Pirenne, V.; Robert, F.; Landais, Y. Enantioselective Total Synthesis of (+)-Eucophylline. *Chem. – Eur. J.* **2022**, *28* (16), e202200088. https://doi.org/10.1002/chem.202200088.

(9) Wang, T.; Shi, S.; Hansmann, M. M.; Rettenmeier, E.; Rudolph, M.; Hashmi, A. S. K. Synthesis of Highly Substituted 3-Formylfurans by a Gold(I)-Catalyzed Oxidation/1,2-Alkynyl Migration/Cyclization Cascade. *Angew. Chem. Int. Ed.* **2014**, *53* (14), 3715–3719. https://doi.org/10.1002/anie.201310146.

(10) Blackwell, D. T. ; G., Warren R. J. D. ;. Spring, David R. Stereoselective Synthesis of Disubstituted Butadienes via Copper-Mediated Coupling of Alkenyl Silanes. *Synlett* **2011**, *2011* (15), 2140–2144. https://doi.org/10.1055/s-0030-1261150.

(11) Yoshida, S.; Fukui, K.; Kikuchi, S.; Yamada, T. Silver-Catalyzed Enantioselective Carbon Dioxide Incorporation into Bispropargylic Alcohols. *J. Am. Chem. Soc.* **2010**, *132* (12), 4072–4073. https://doi.org/10.1021/ja1007118.

NMR Spectra of New Compounds

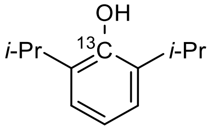

^{1}H NMR of 2,6-diisopropylphenol-*1*-^{13}C (**4a-*1*-^{13}C**) in $CDCl_3$, 400 MHz

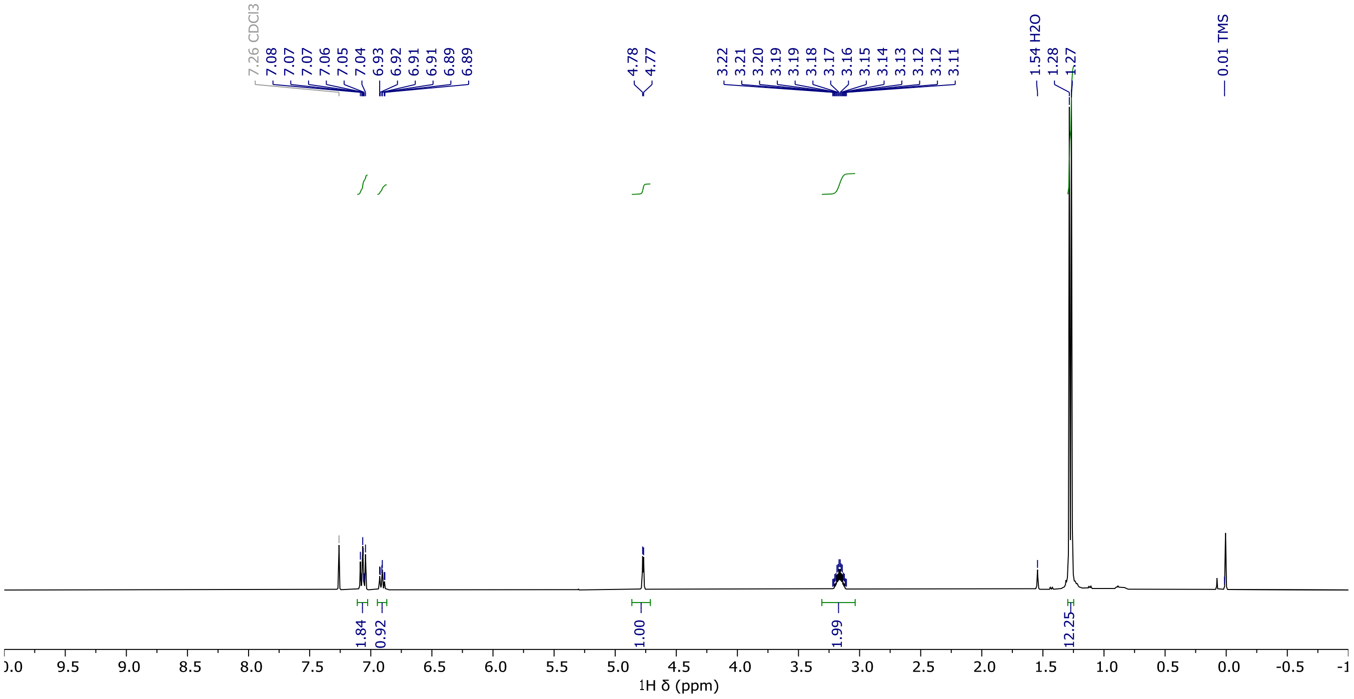

^{13}C{^{1}H} NMR of 2,6-diisopropylphenol-*1*-^{13}C (**4a-*1*-^{13}C**) in $CDCl_3$, 101 MHz

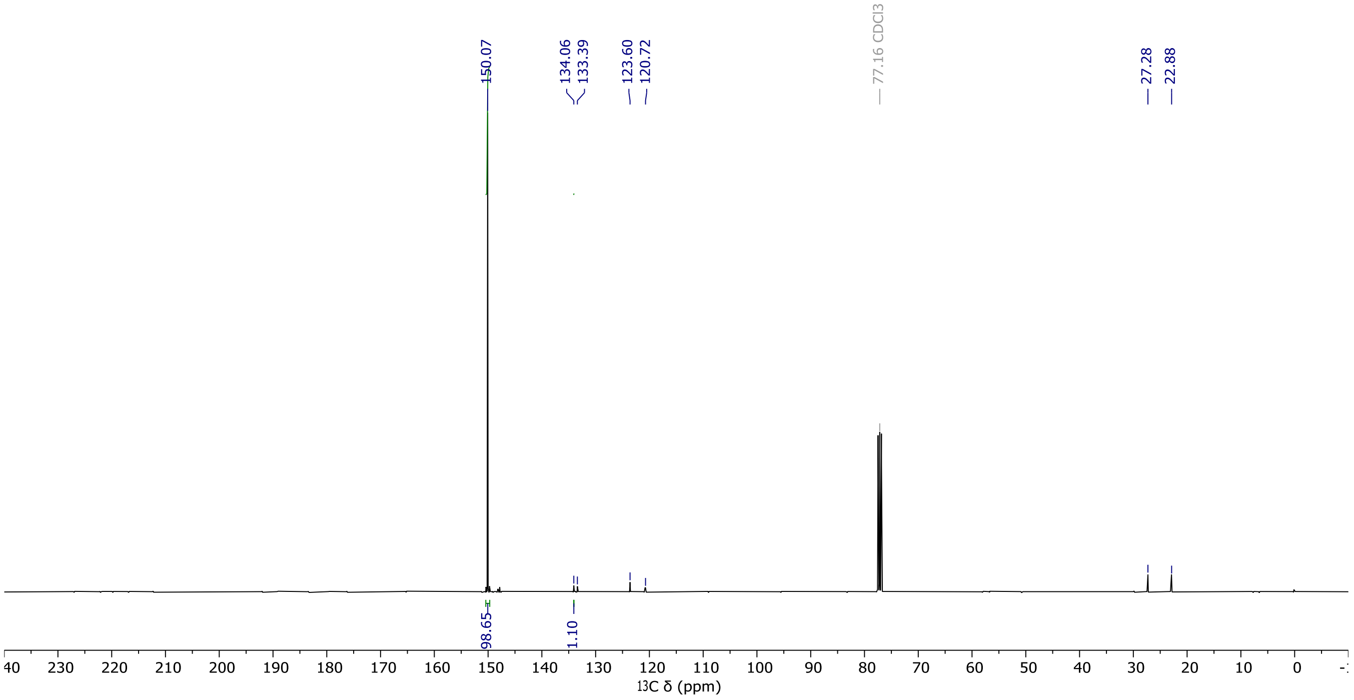

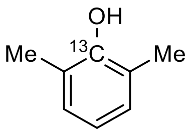

^{1}H NMR of 2,6-dimethylphenol-*1*-^{13}C (**4b-*1*-^{13}C**) in $CDCl_3$, 400 MHz

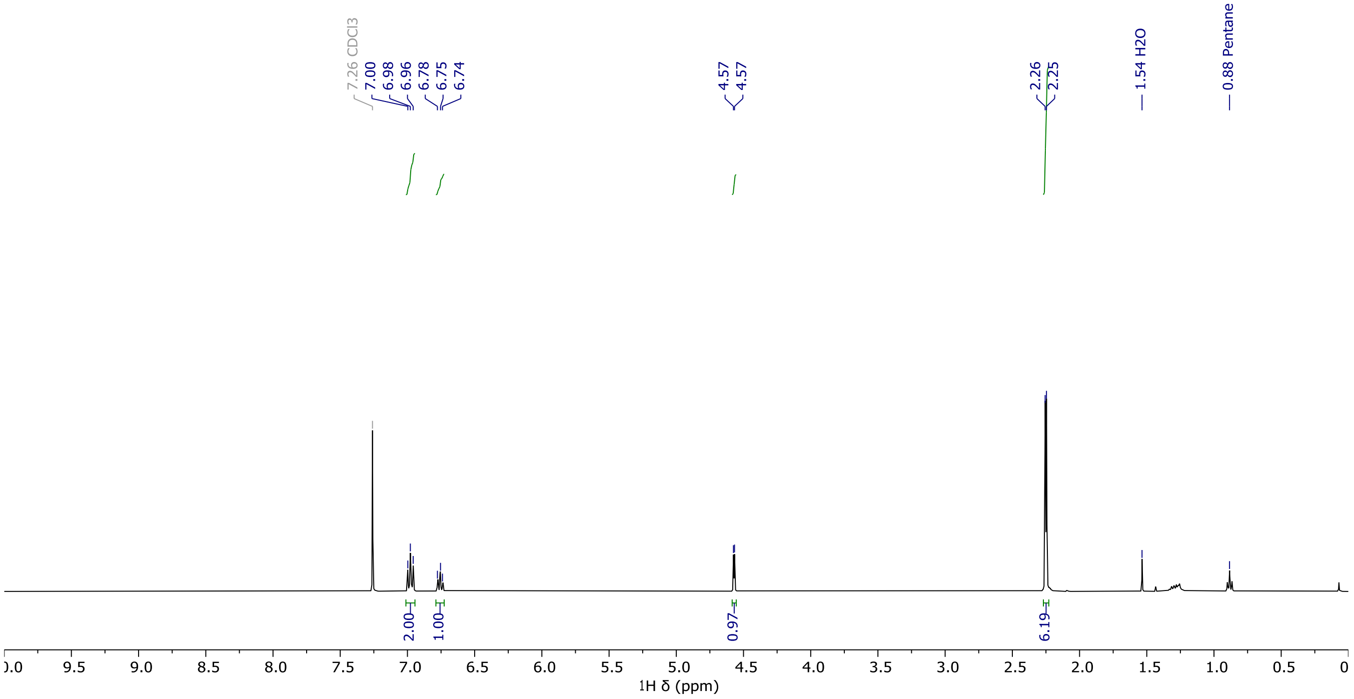

$^{13}C\{^{1}H\}$ NMR of 2,6-dimethylphenol-*1*-^{13}C (**4b-*1*-^{13}C**) in $CDCl_3$, 101 MHz

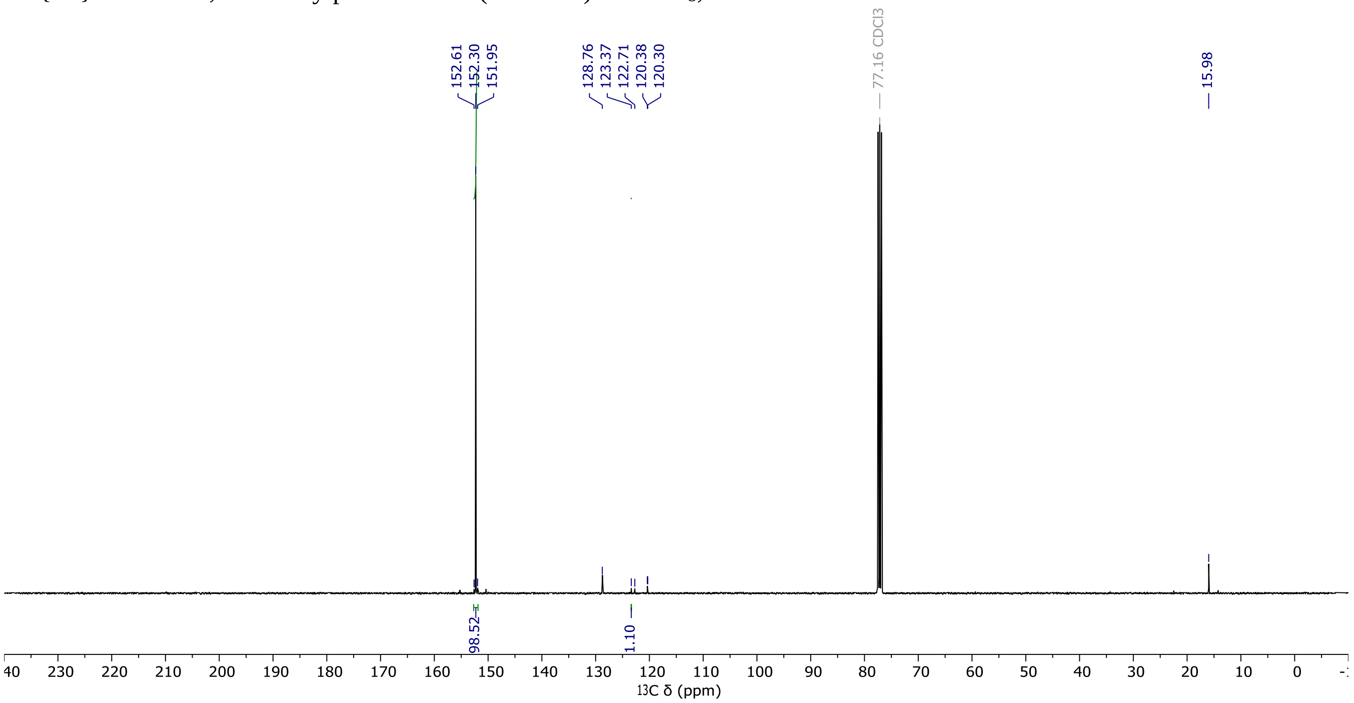

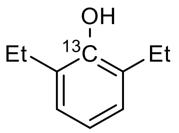

^{1}H NMR of 2,6-diethylphenol-*1*-^{13}C (**4c-*1*-^{13}C**) in $CDCl_3$, 400 MHz

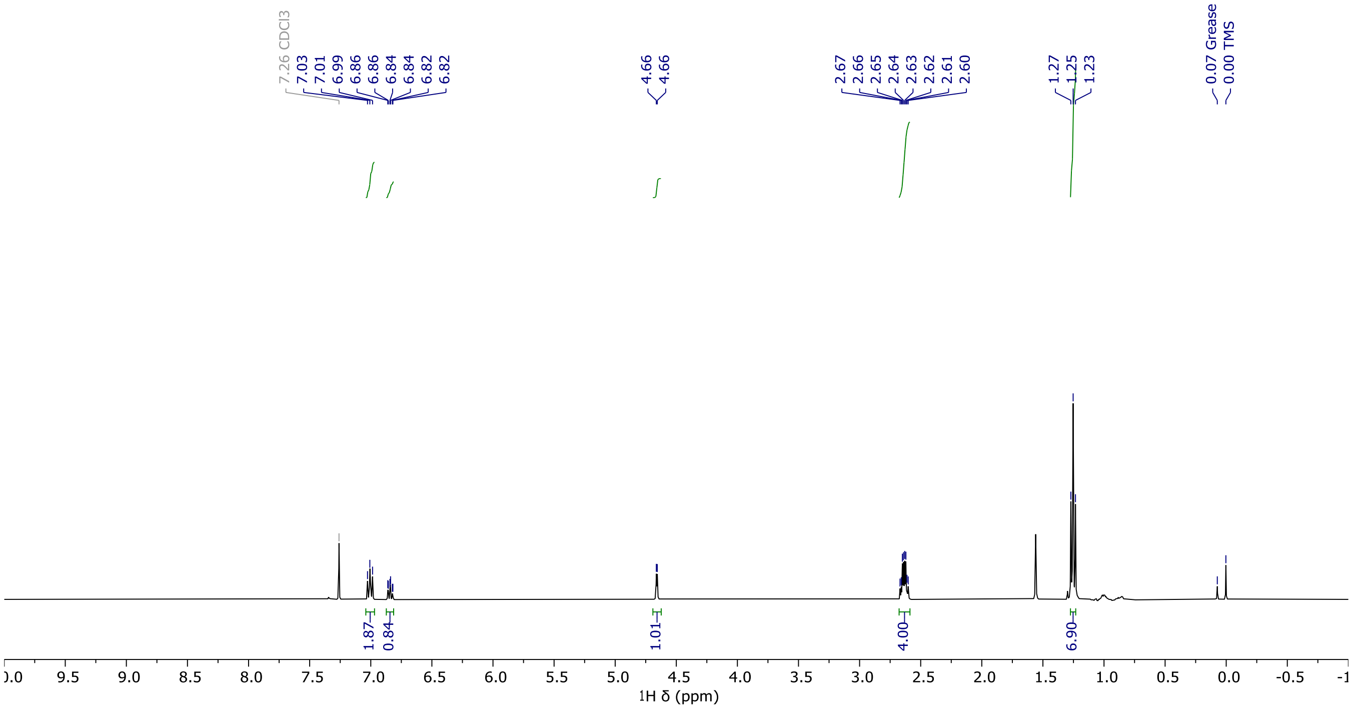

$^{13}C\{^{1}H\}$ NMR of 2,6-diethylphenol-*1*-^{13}C (**4c-*1*-^{13}C**) in $CDCl_3$, 101 MHz

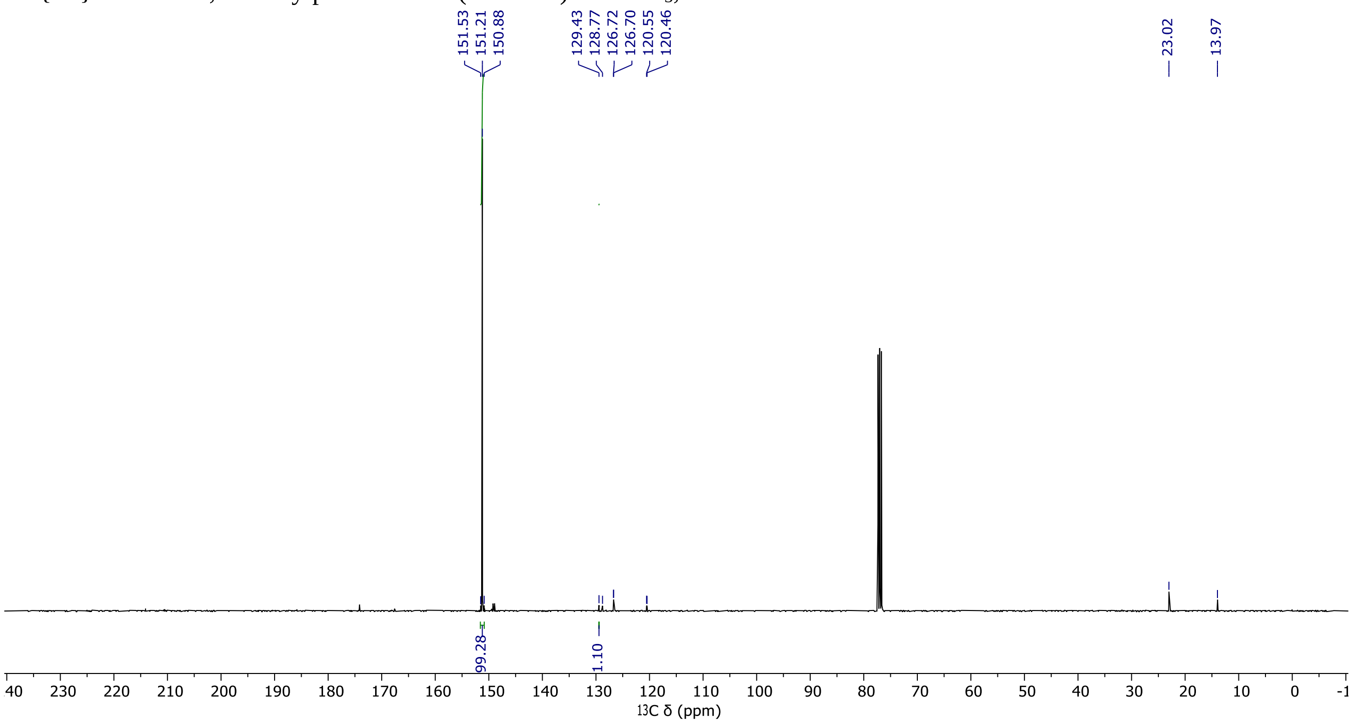

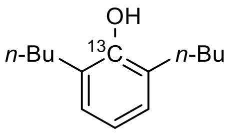

^{1}H NMR of 2,6-dibutylphenol-*1*-^{13}C (**4d-*1*-^{13}C**) in $CDCl_3$, 400 MHz

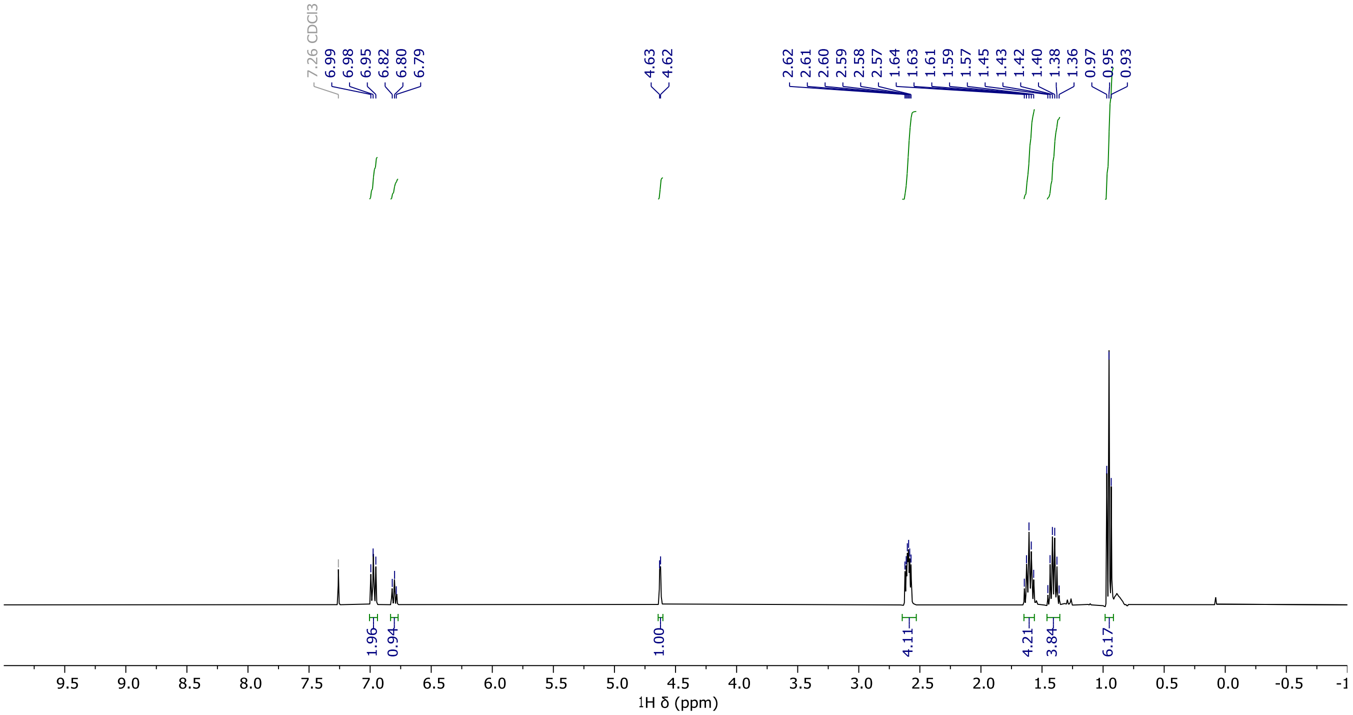

^{13}C{^{1}H} NMR of 2,6-dibutylphenol-*1*-^{13}C (**4d-*1*-^{13}C**) in $CDCl_3$, 101 MHz

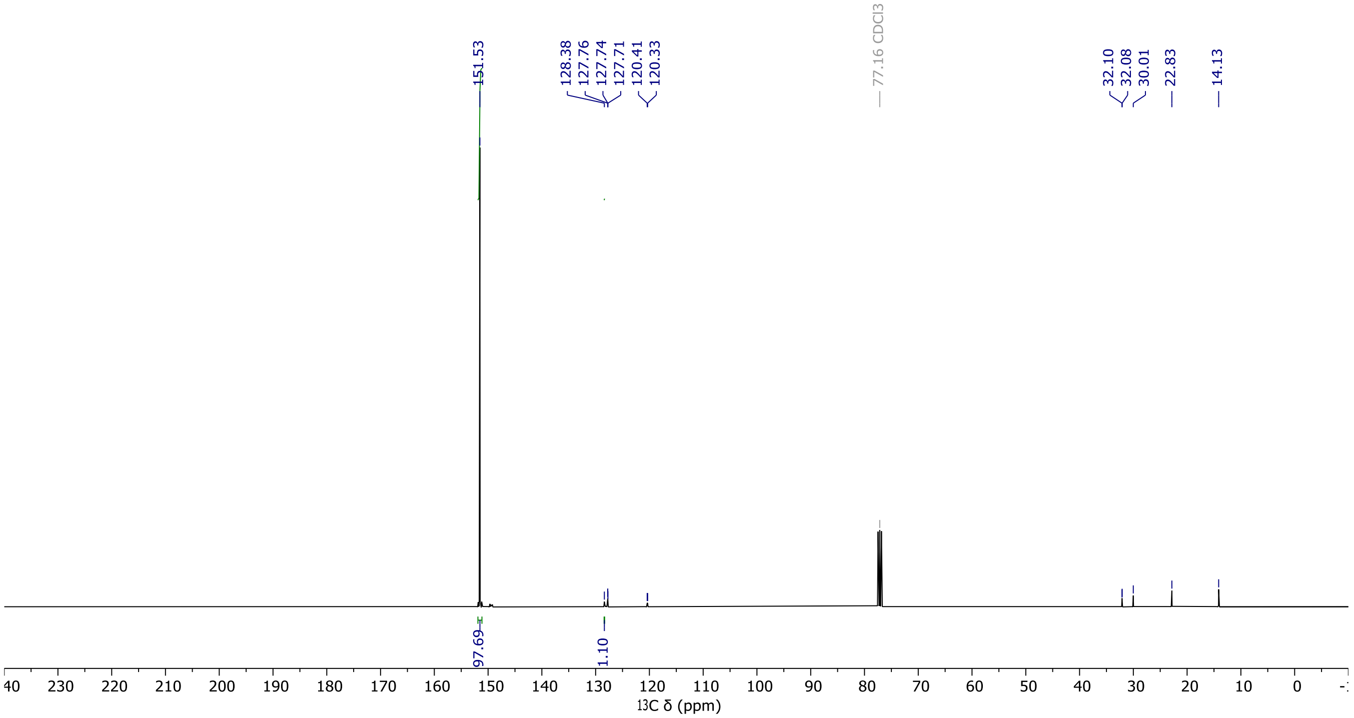

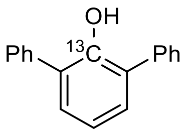

^{1}H NMR of 2,6-diphenylphenol-*1*-^{13}C (**4e-*1*-^{13}C**) in $CDCl_3$, 400 MHz

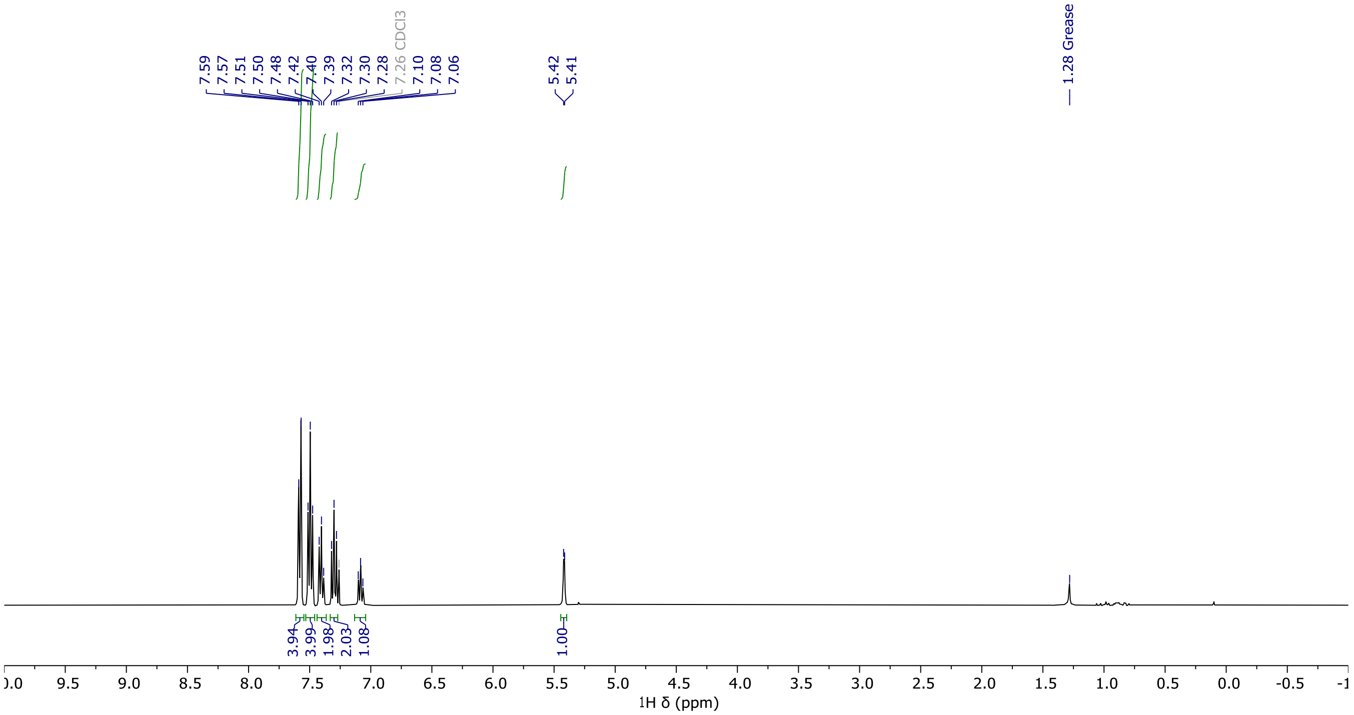

^{13}C{^{1}H} NMR of 2,6-diphenylphenol-*1*-^{13}C (**4e-*1*-^{13}C**) in $CDCl_3$, 101 MHz

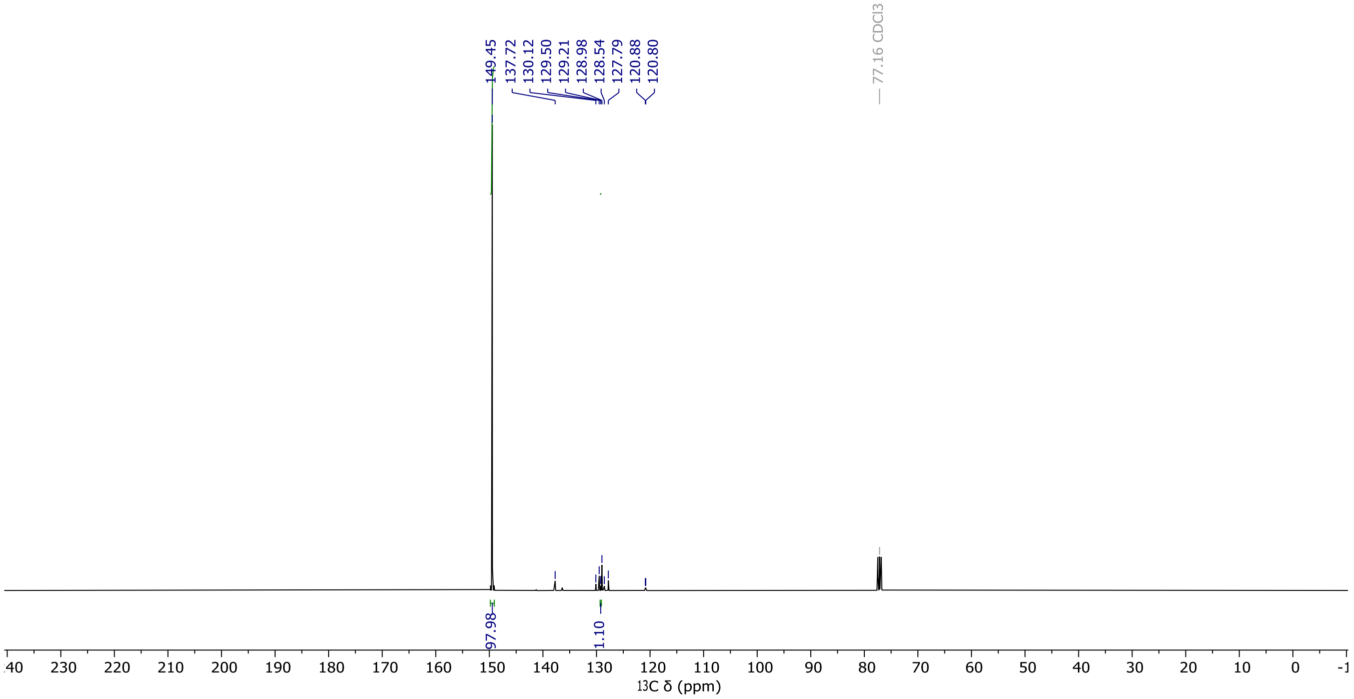

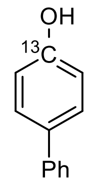

^{1}H NMR of 4-phenylphenol-*1*-^{13}C (**4f-*1*-^{13}C**) in $CDCl_3$, 400 MHz

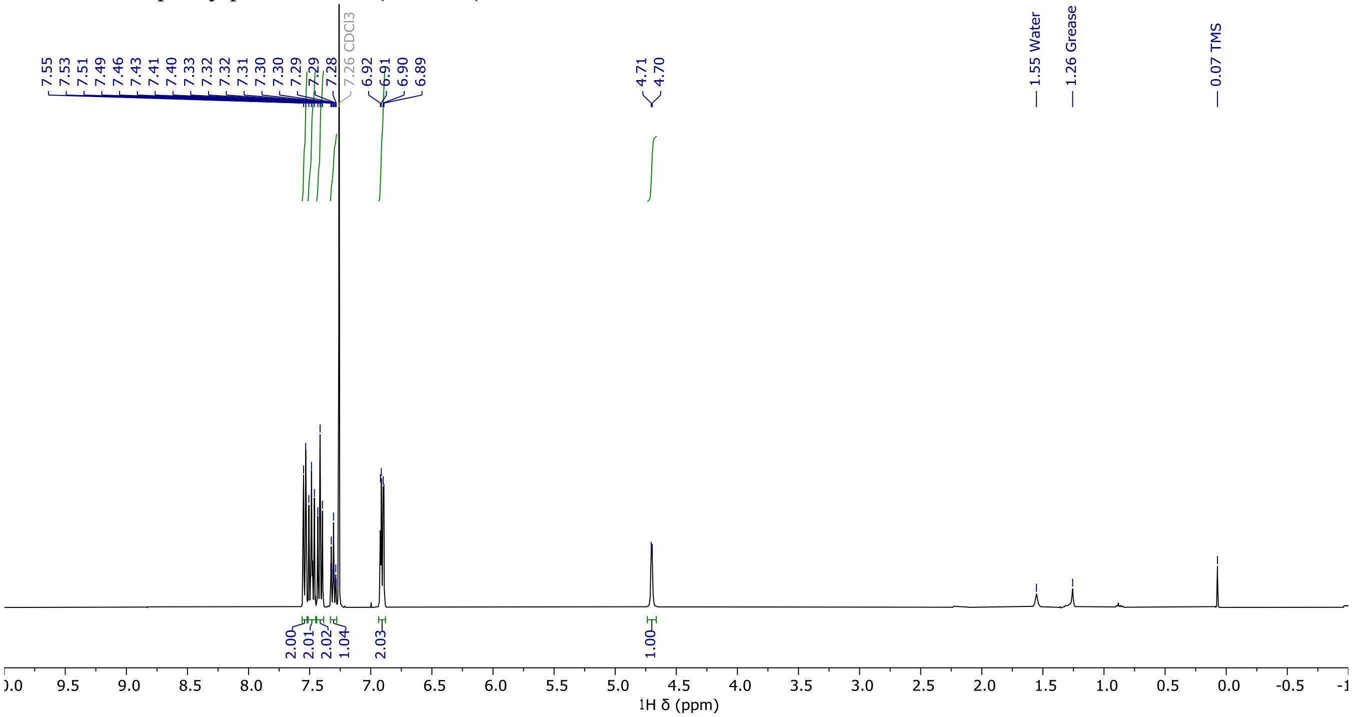

$^{13}C\{^{1}H\}$ NMR of 4-phenylphenol-*1*-^{13}C (**4f-*1*-^{13}C**) in $CDCl_3$, 101 MHz

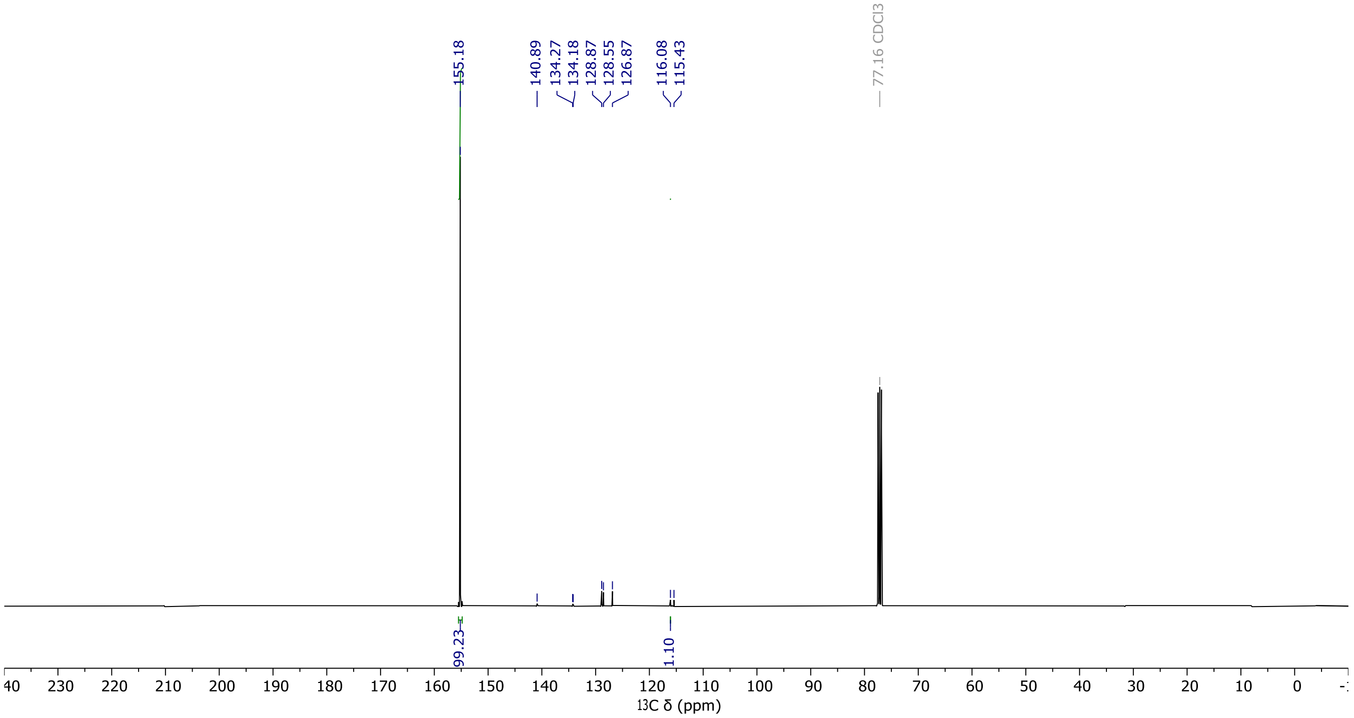

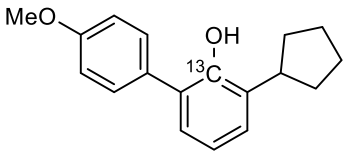

^{1}H NMR of 2-cyclopentyl-6-(4-methoxyphenyl)phenol-*1*-^{13}C (**4g-*1*-^{13}C**) in $CDCl_3$, 400 MHz

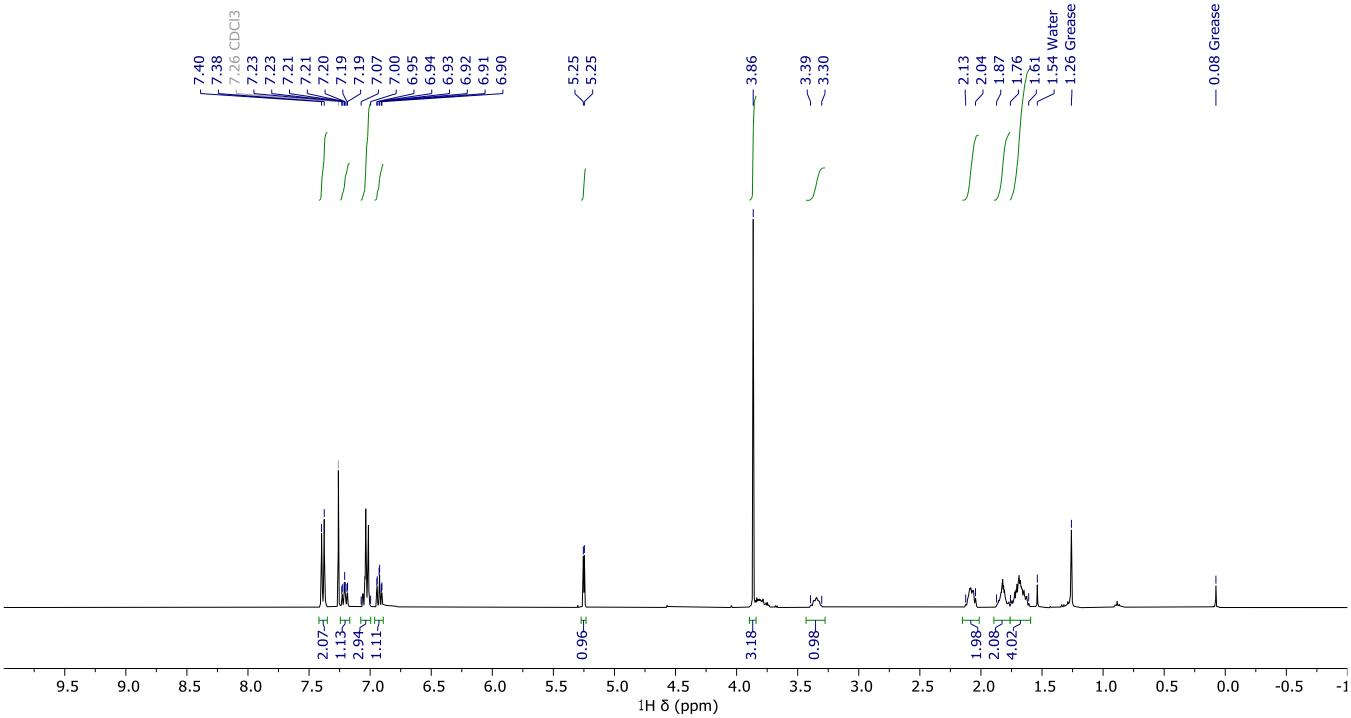

^{13}C{^{1}H} NMR of 2-cyclopentyl-6-(4-methoxyphenyl)phenol-*1*-^{13}C (**4g-*1*-^{13}C**) in $CDCl_3$, 101 MHz

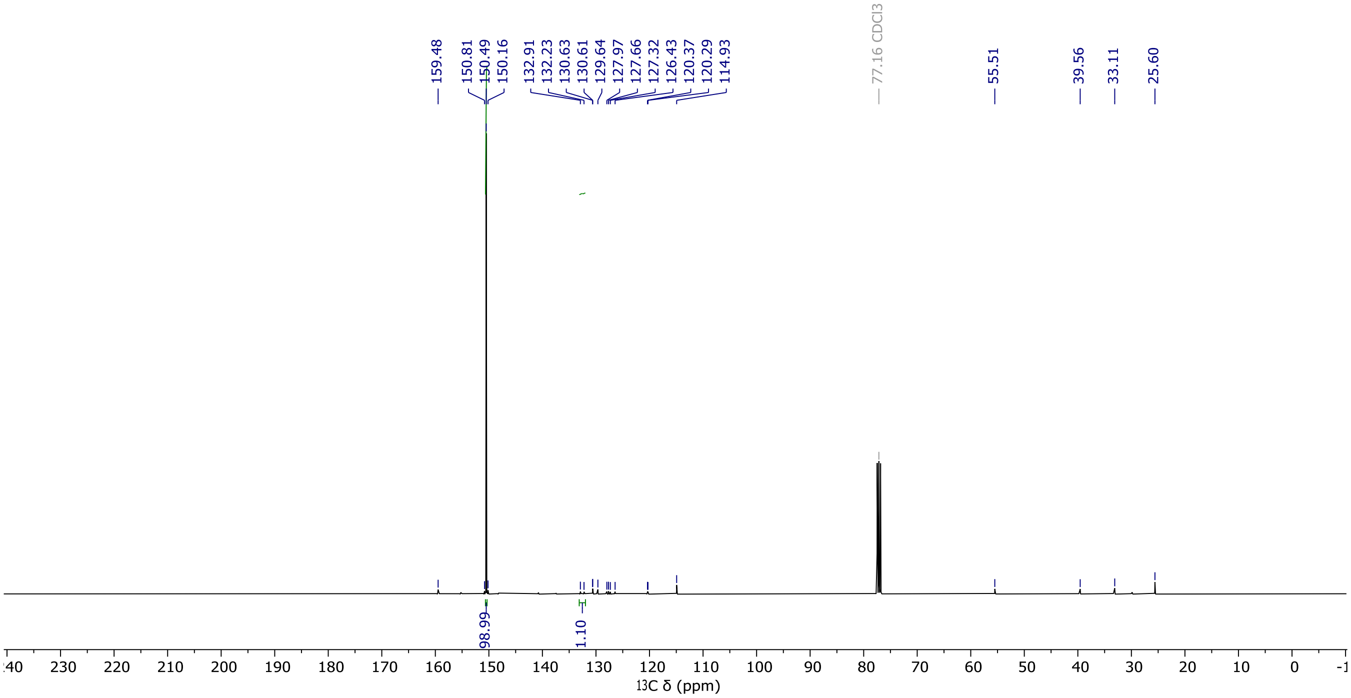

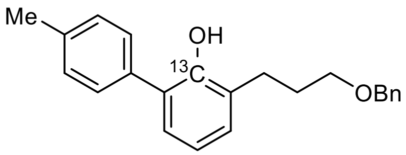

^{1}H NMR of 2-(3-(benzyloxy)propyl)-6-(*p*-tolyl)phenol-*1*-^{13}C (**4h-*1*-^{13}C**) in $CDCl_3$, 400 MHz

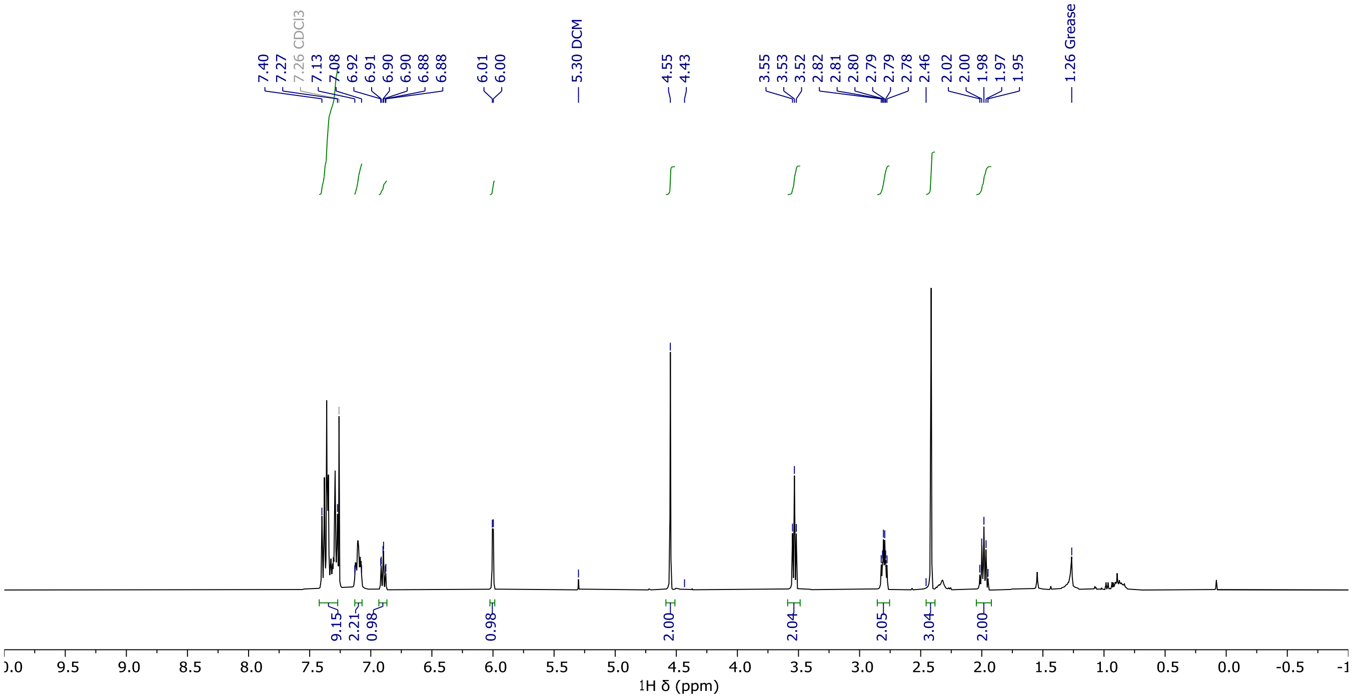

^{13}C{^{1}H} NMR of 2-(3-(benzyloxy)propyl)-6-(*p*-tolyl)phenol-*1*-^{13}C (**4h-*1*-^{13}C**) in $CDCl_3$, 101 MHz

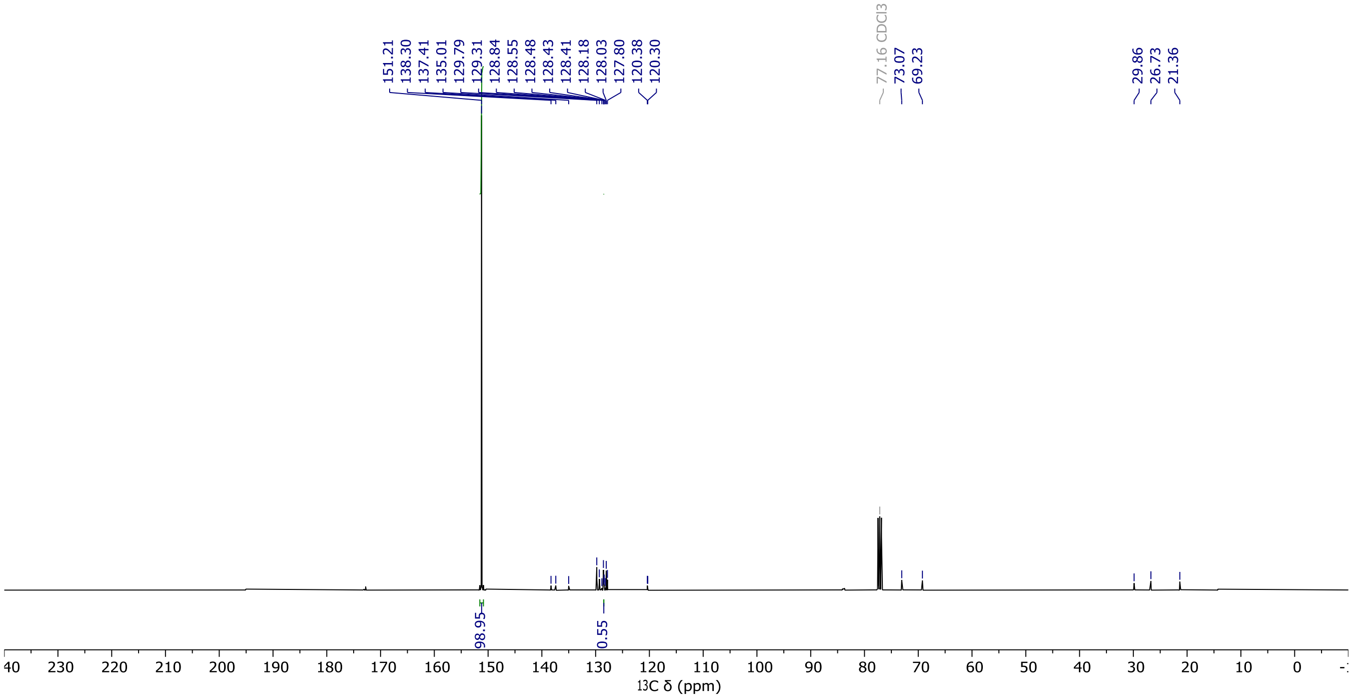

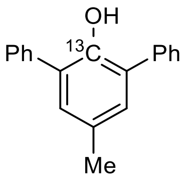

^{1}H NMR of 4-methyl-2,6-diphenylphenol-*1*-^{13}C (**4i-*1*-^{13}C**) in $CDCl_3$, 400 MHz

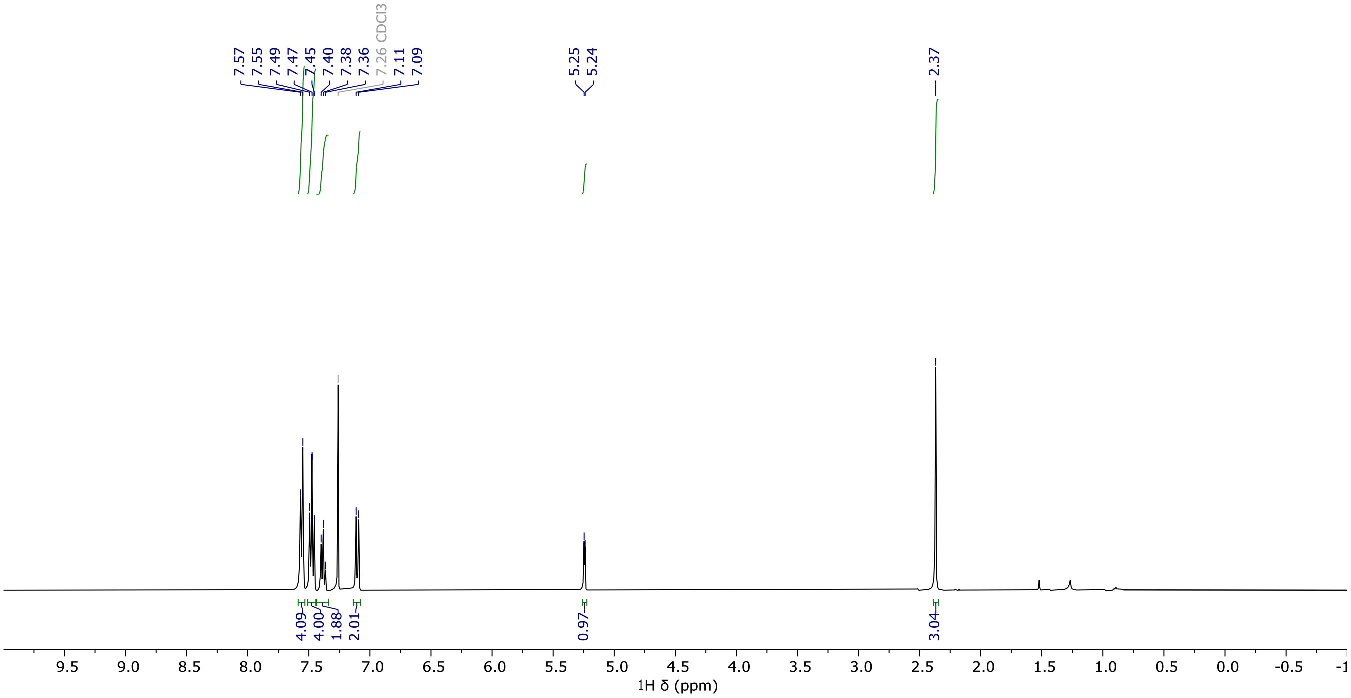

^{13}C{^{1}H} NMR of 4-methyl-2,6-diphenylphenol-*1*-^{13}C (**4i-*1*-^{13}C**) in $CDCl_3$, 101 MHz

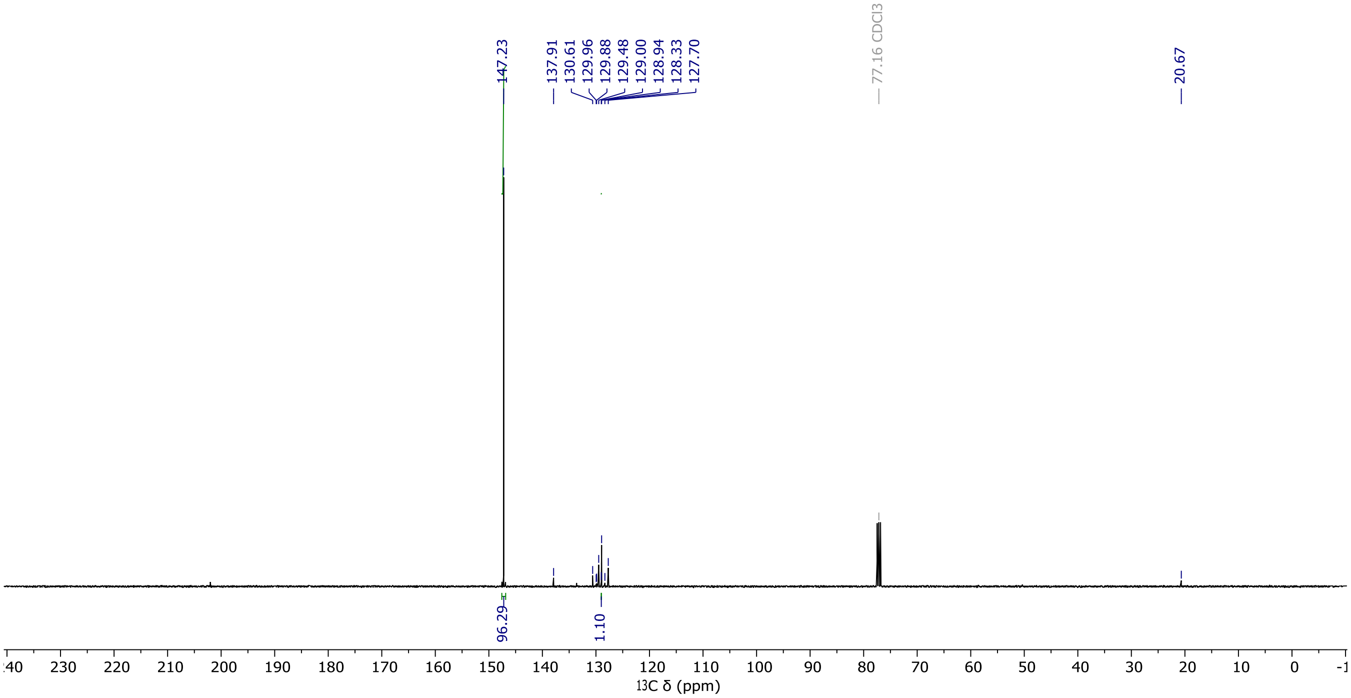

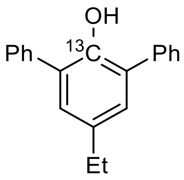

^{1}H NMR of 4-ethyl-2,6-diphenylphenol-*1*-^{13}C (**4j-*1*-^{13}C**) in $CDCl_3$, 400 MHz

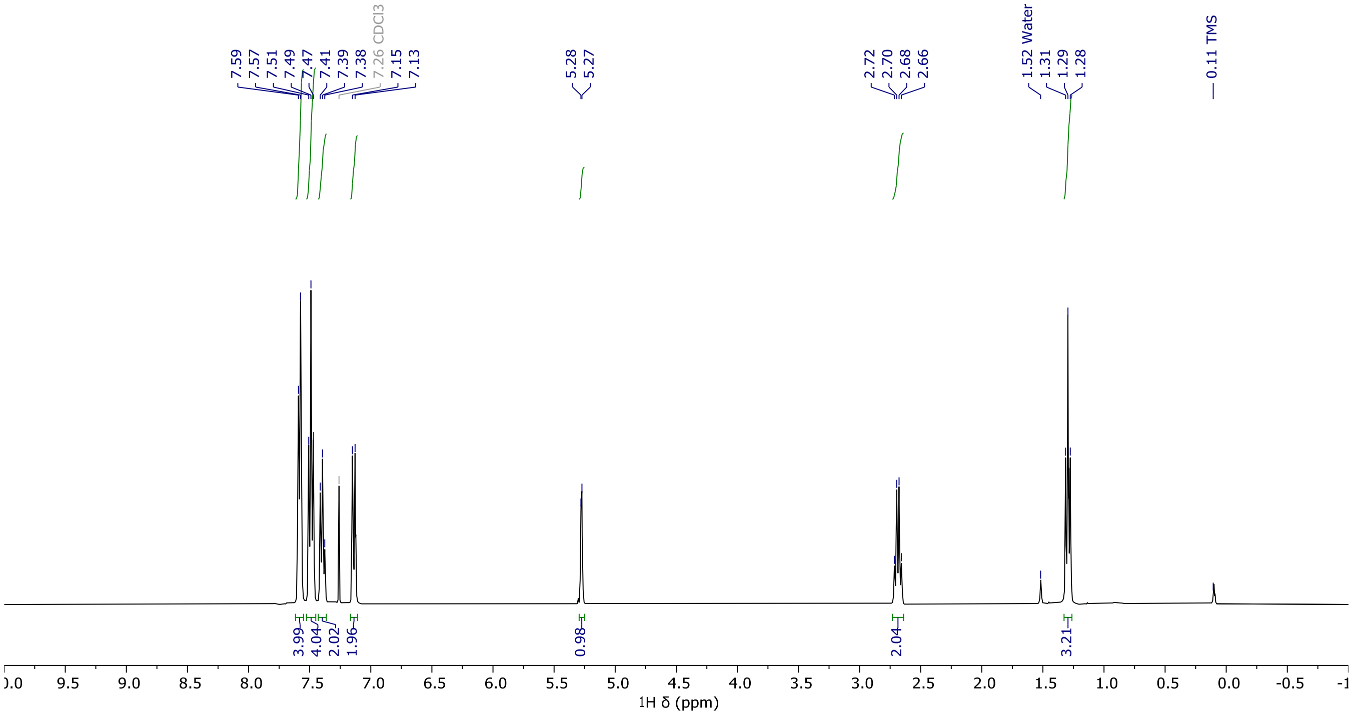

^{13}C{^{1}H} NMR of 4-ethyl-2,6-diphenylphenol-*1*-^{13}C (**4j-*1*-^{13}C**) in $CDCl_3$, 101 MHz

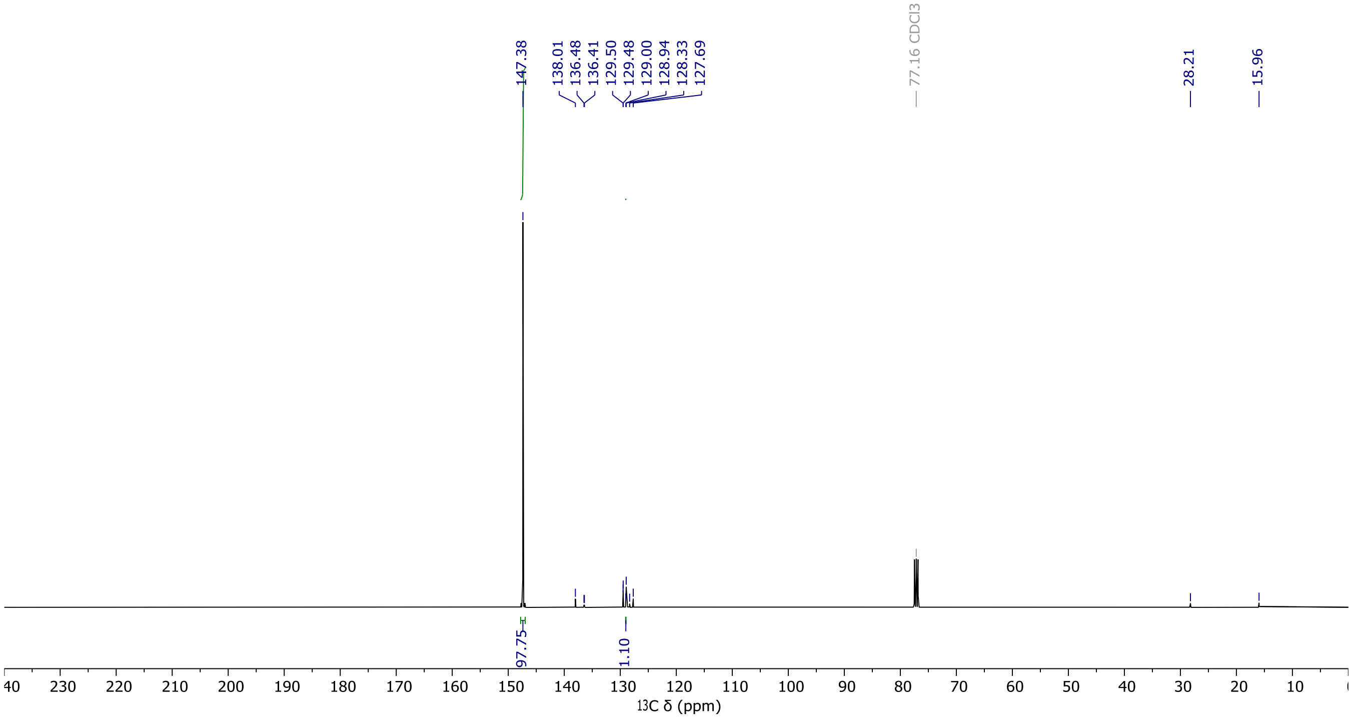

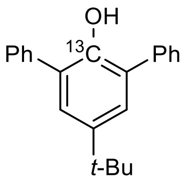

^{1}H NMR of 4-(*tert*-butyl)-2,6-diphenylphenol-*1*-^{13}C (**4k-*1*-^{13}C**) in $CDCl_3$, 400 MHz

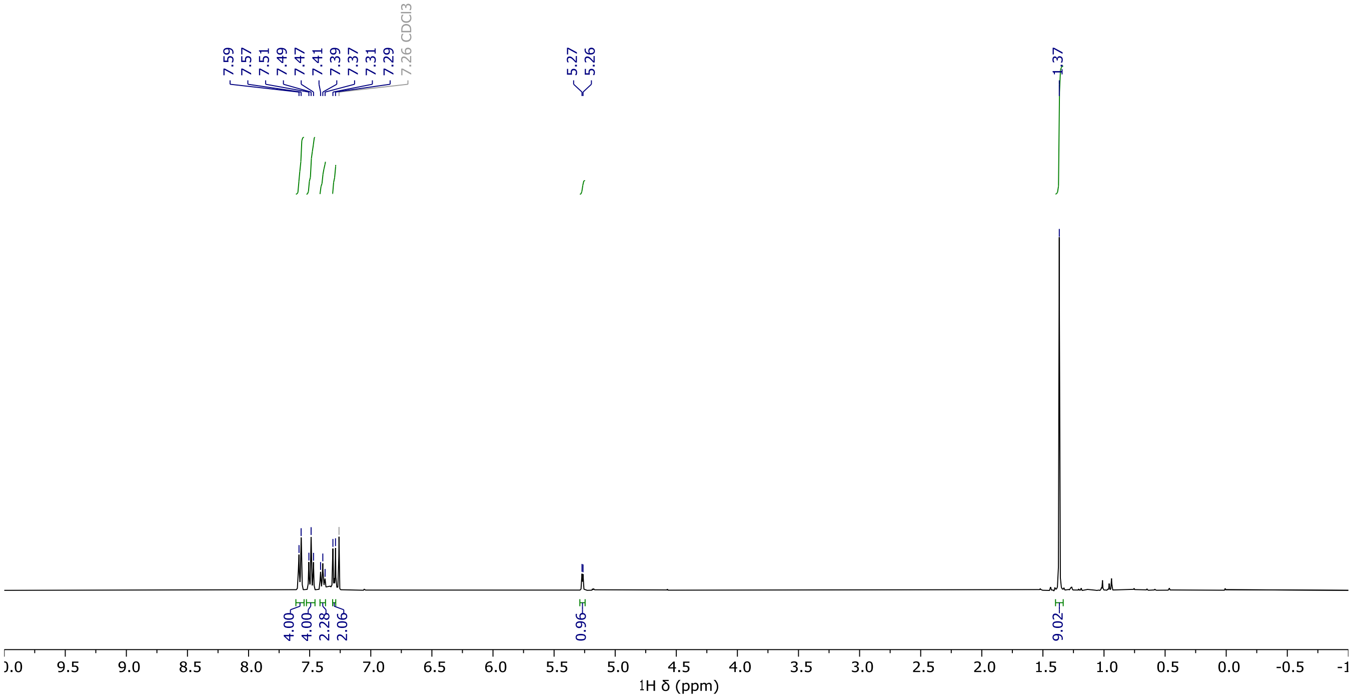

^{13}C{^{1}H} NMR of 4-(*tert*-butyl)-2,6-diphenylphenol-*1*-^{13}C (**4k-*1*-^{13}C**) in $CDCl_3$, 101 MHz

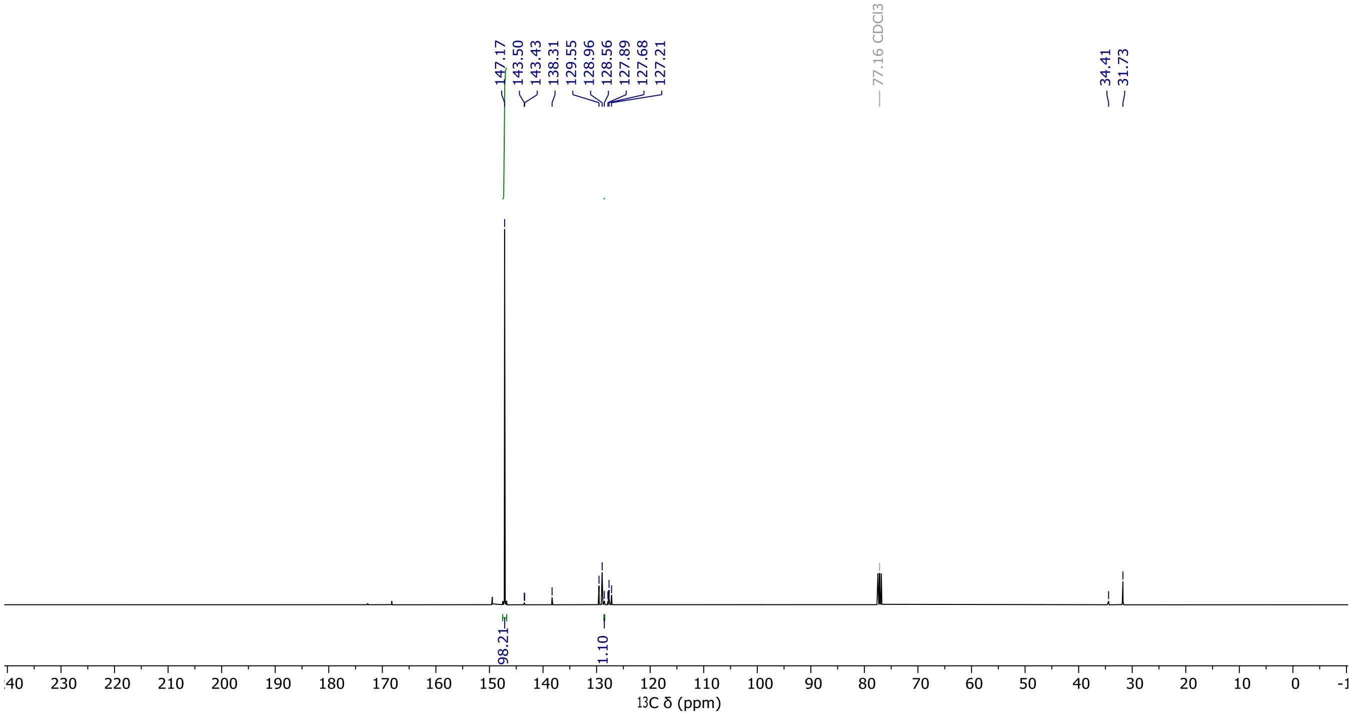

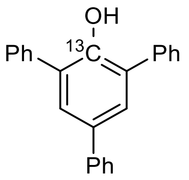

^{1}H NMR of 2,4,6-triphenylphenol-*1*-^{13}C (**4l-*1*-^{13}C**) in $CDCl_3$, 400 MHz

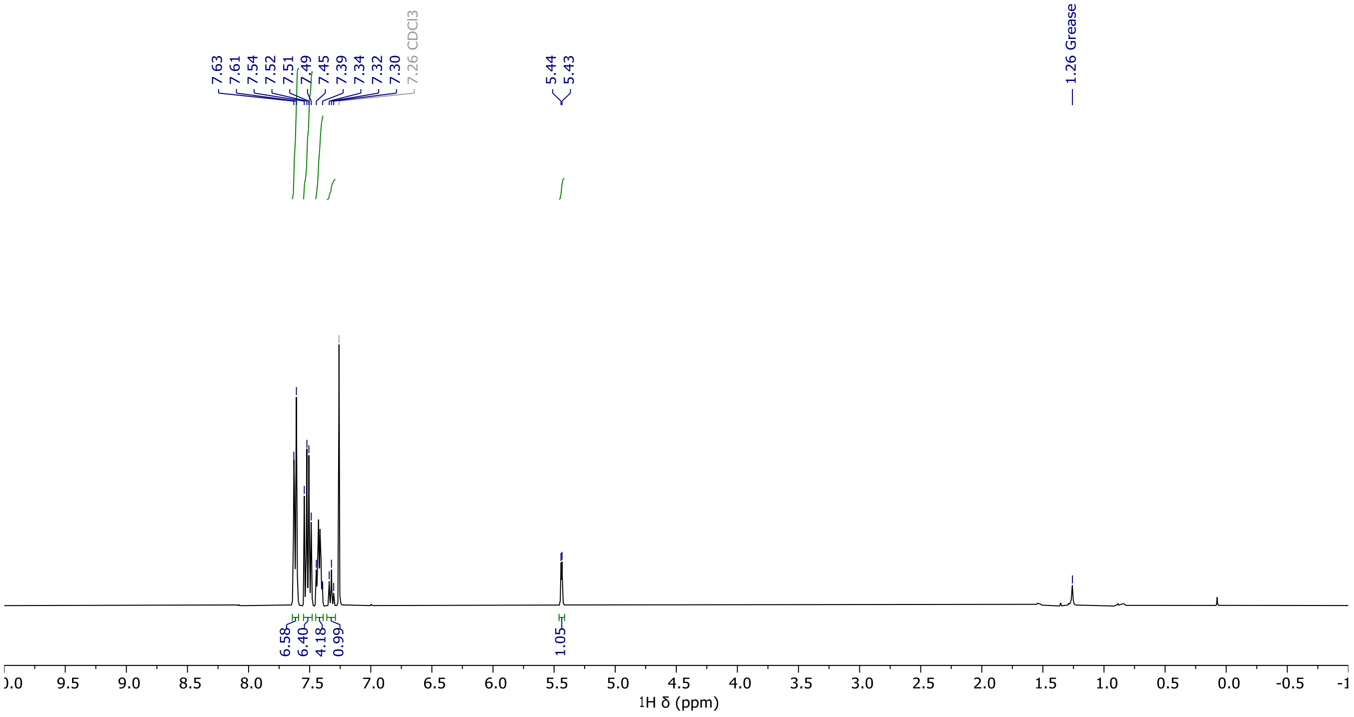

^{13}C{^{1}H} NMR of 2,4,6-triphenylphenol-*1*-^{13}C (**4l-*1*-^{13}C**) in $CDCl_3$, 101 MHz

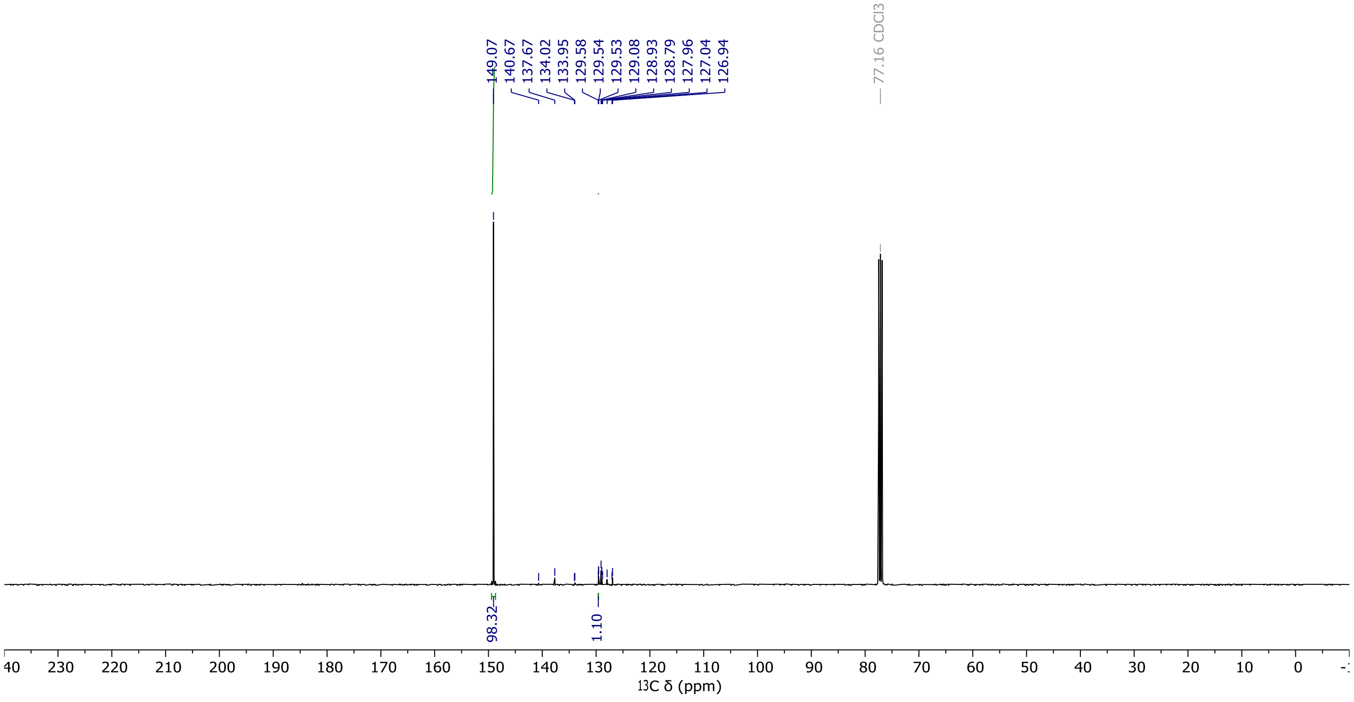

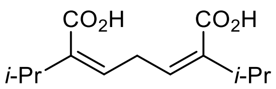

^{1}H NMR of (2*Z*,5*Z*)-2,6-diisopropylhepta-2,5-dienedioic acid (**6**) in $CDCl_3$, 500 MHz

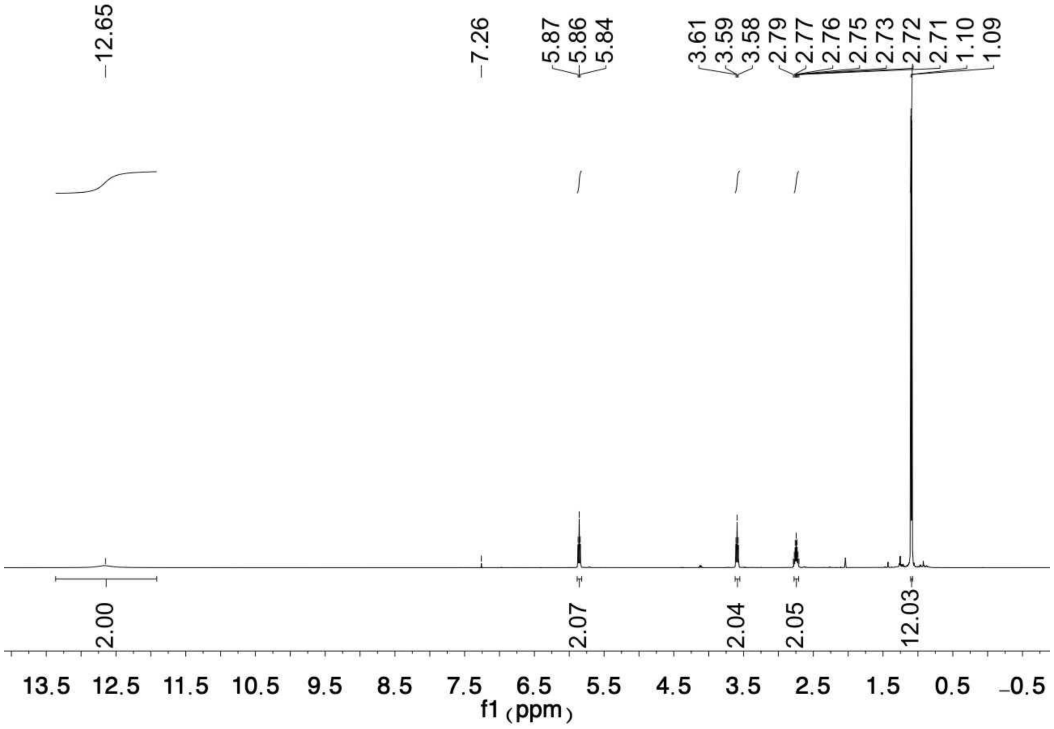

^{13}C NMR of (2*Z*,5*Z*)-2,6-diisopropylhepta-2,5-dienedioic acid (**6**) in $CDCl_3$, 126 MHz

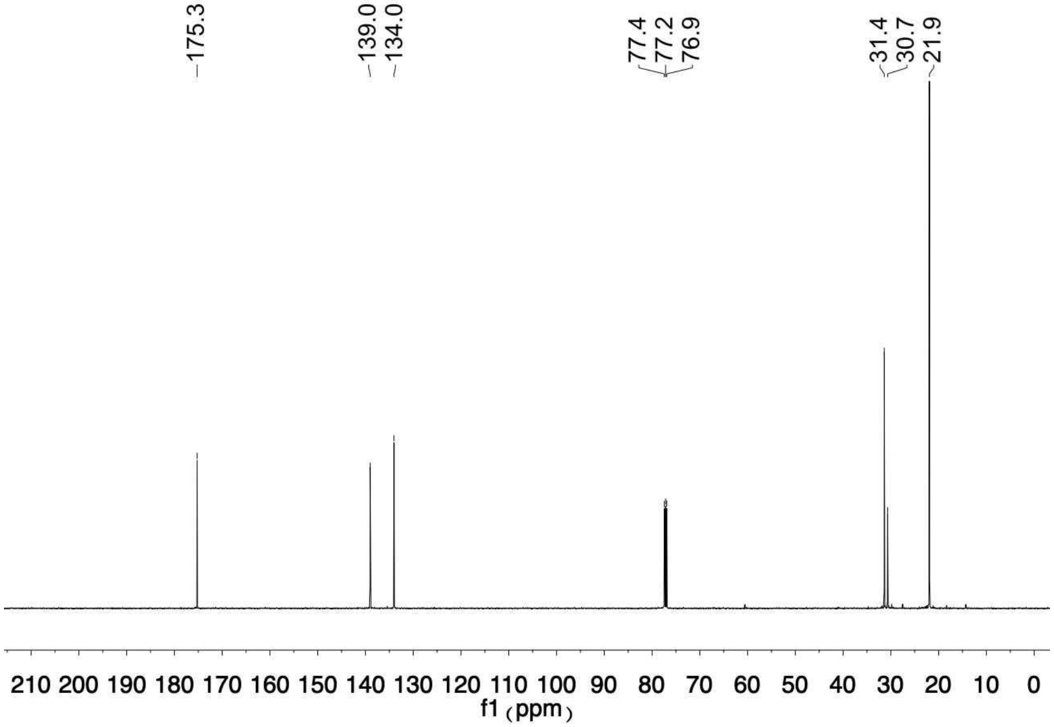

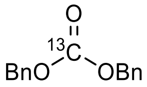

^{1}H NMR of dibenzyl carbonate-*carbonyl*-^{13}C (**5-*carbonyl*-^{13}C**) in $CDCl_3$, 400 MHz

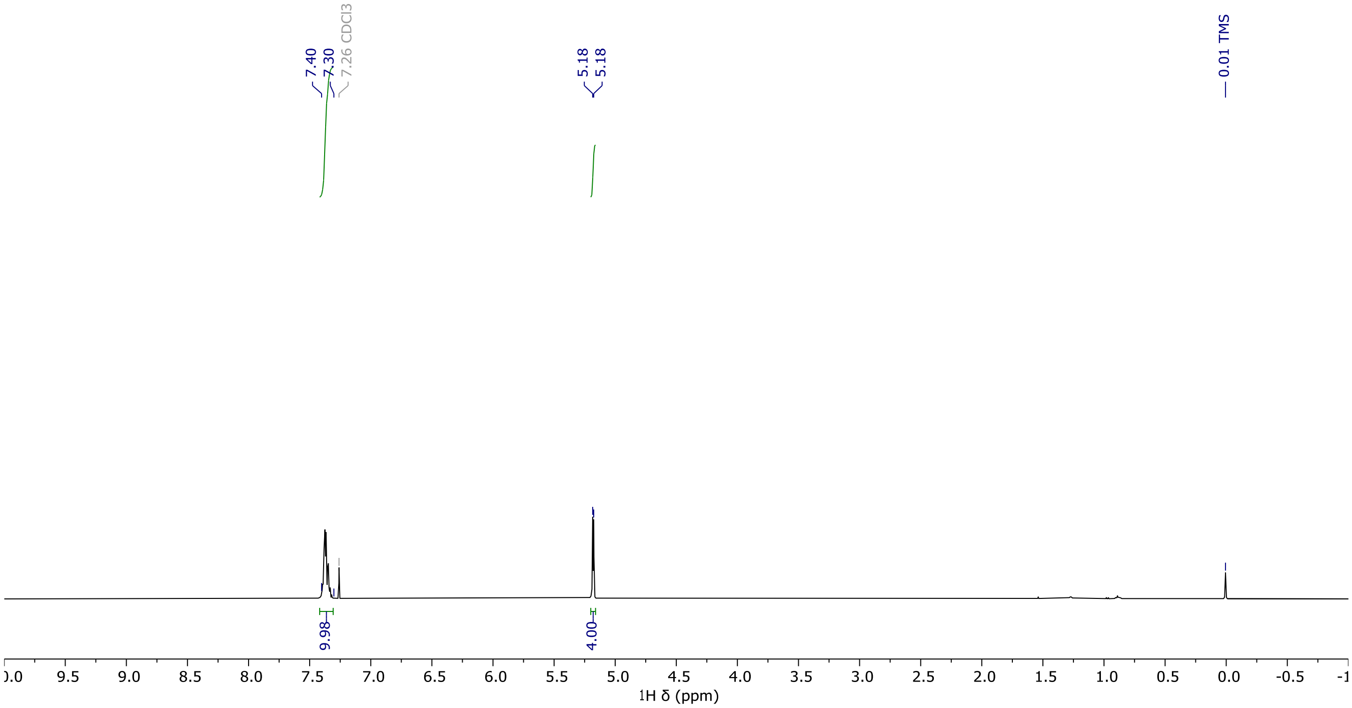

^{13}C{^{1}H} NMR of dibenzyl carbonate-*carbonyl*-^{13}C (**5-*carbonyl*-^{13}C**) in $CDCl_3$, 101 MHz

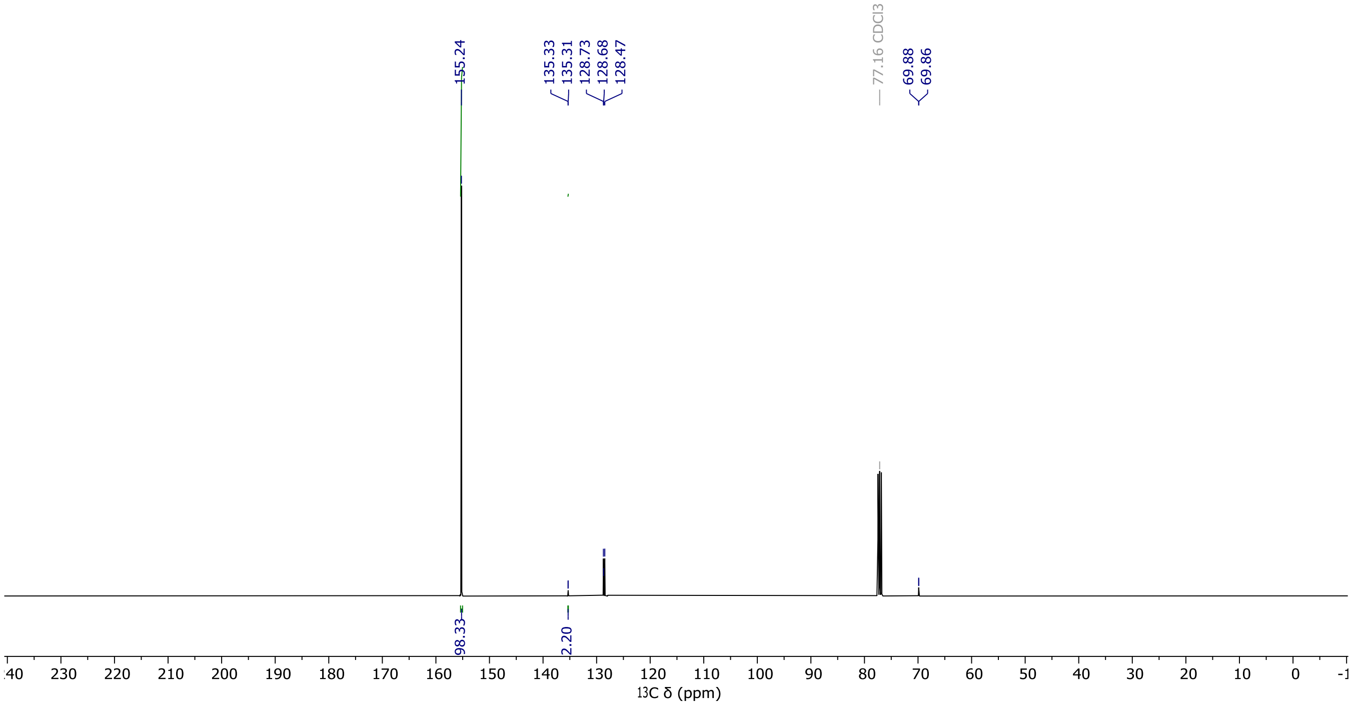

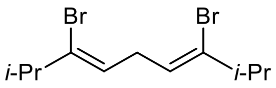

^{1}H NMR of (3*Z*,6*Z*)-3,7-dibromo-2,8-dimethylnona-3,6-diene (**3a**) in $CDCl_3$, 400 MHz

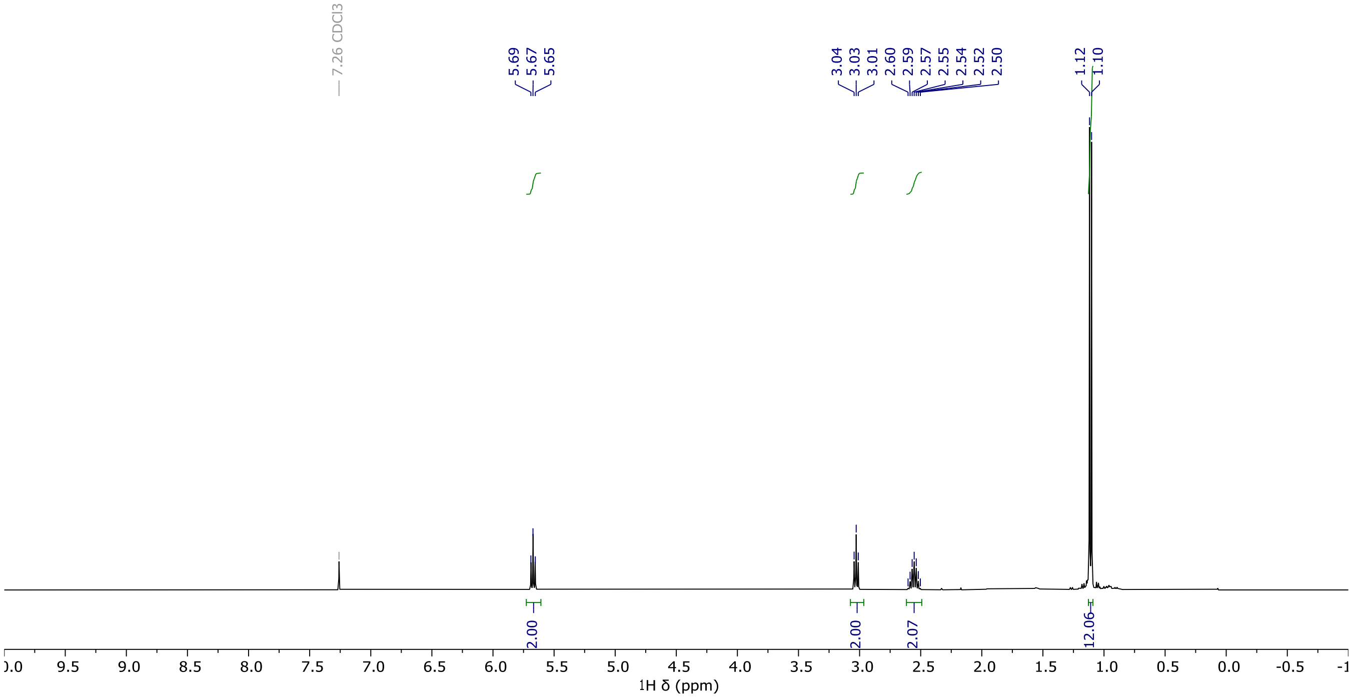

^{13}C{^{1}H} NMR of (3*Z*,6*Z*)-3,7-dibromo-2,8-dimethylnona-3,6-diene (**3a**) in $CDCl_3$, 101 MHz

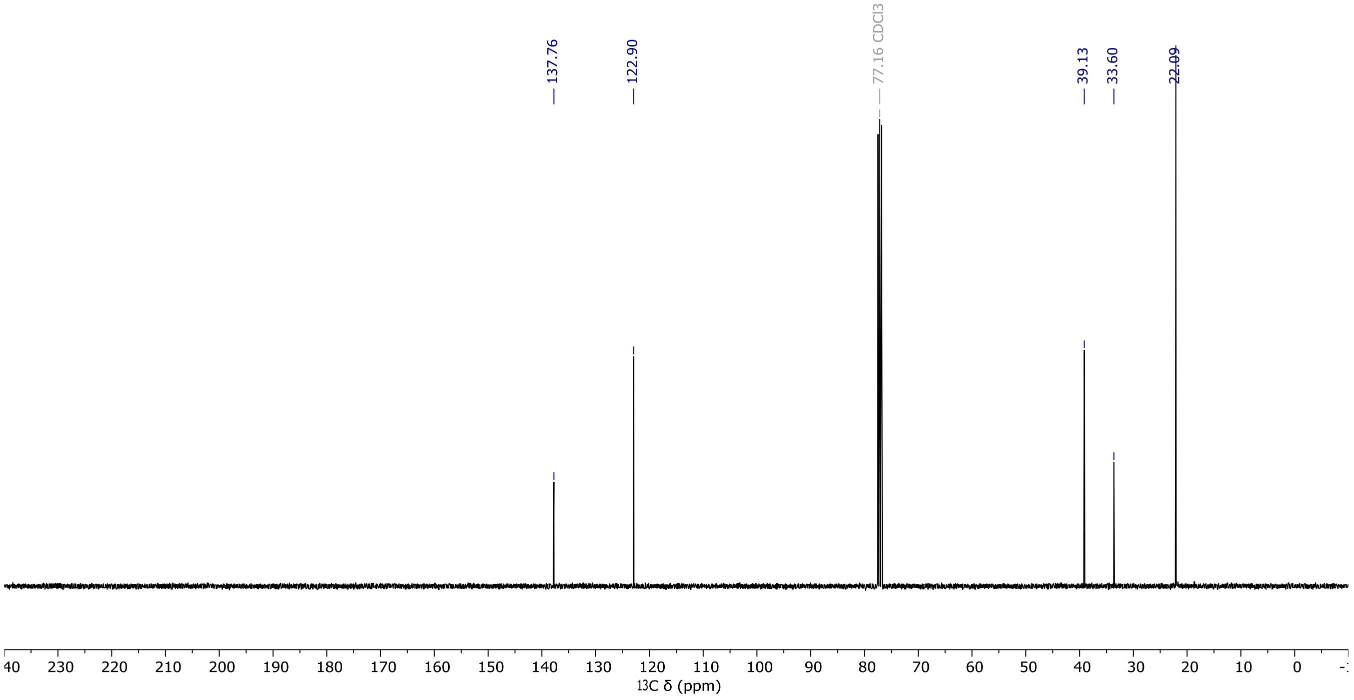

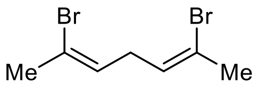

^{1}H NMR of (2*Z*,5*Z*)-2,6-dibromohepta-2,5-diene (**3b**) in $CDCl_3$, 400 MHz

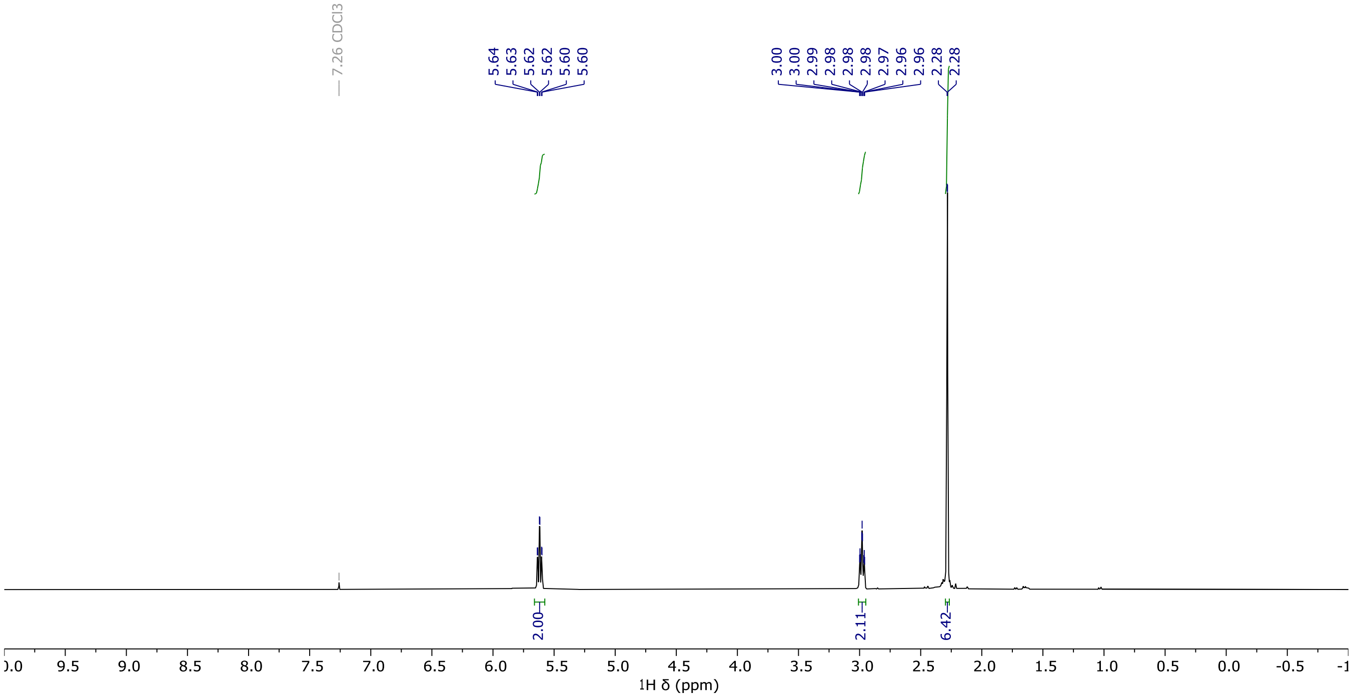

^{13}C{^{1}H} NMR of (2*Z*,5*Z*)-2,6-dibromohepta-2,5-diene (**3b**) in $CDCl_3$, 101 MHz

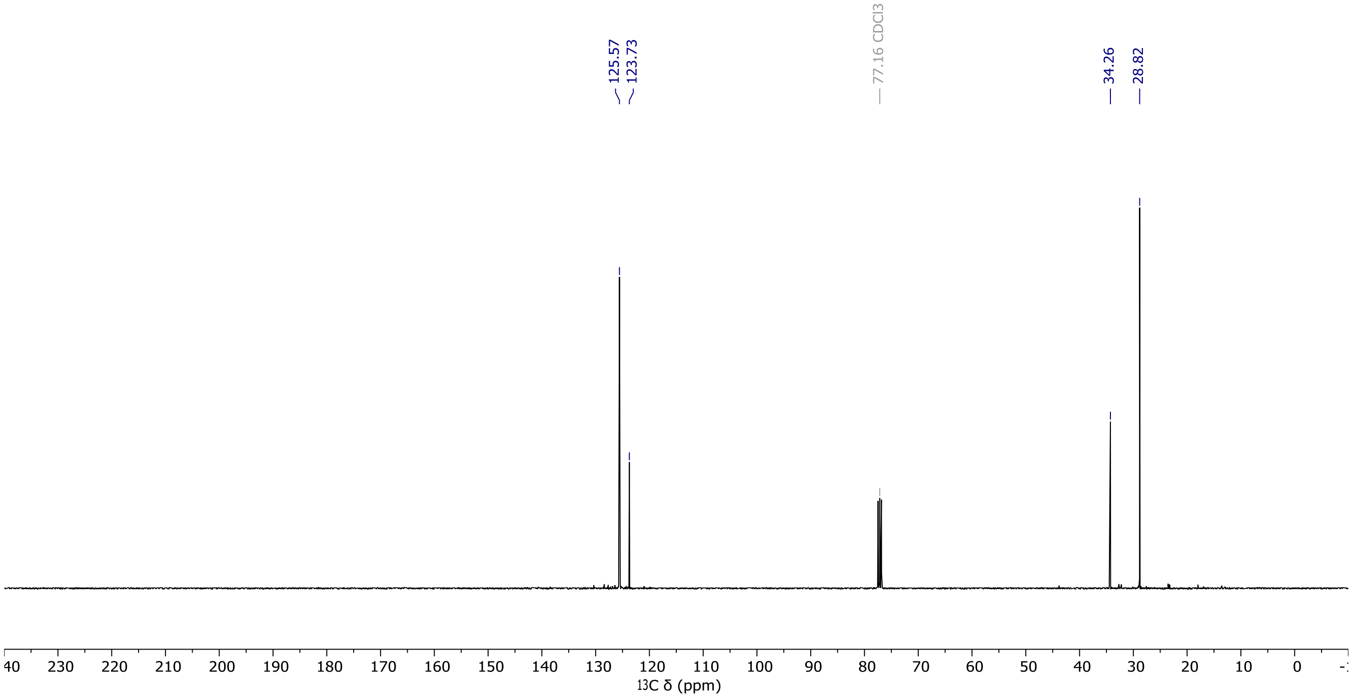

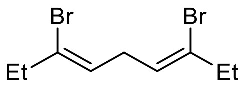

^{1}H NMR of (3*Z*,6*Z*)-3,7-dibromonona-3,6-diene (**3c**) in $CDCl_3$, 400 MHz

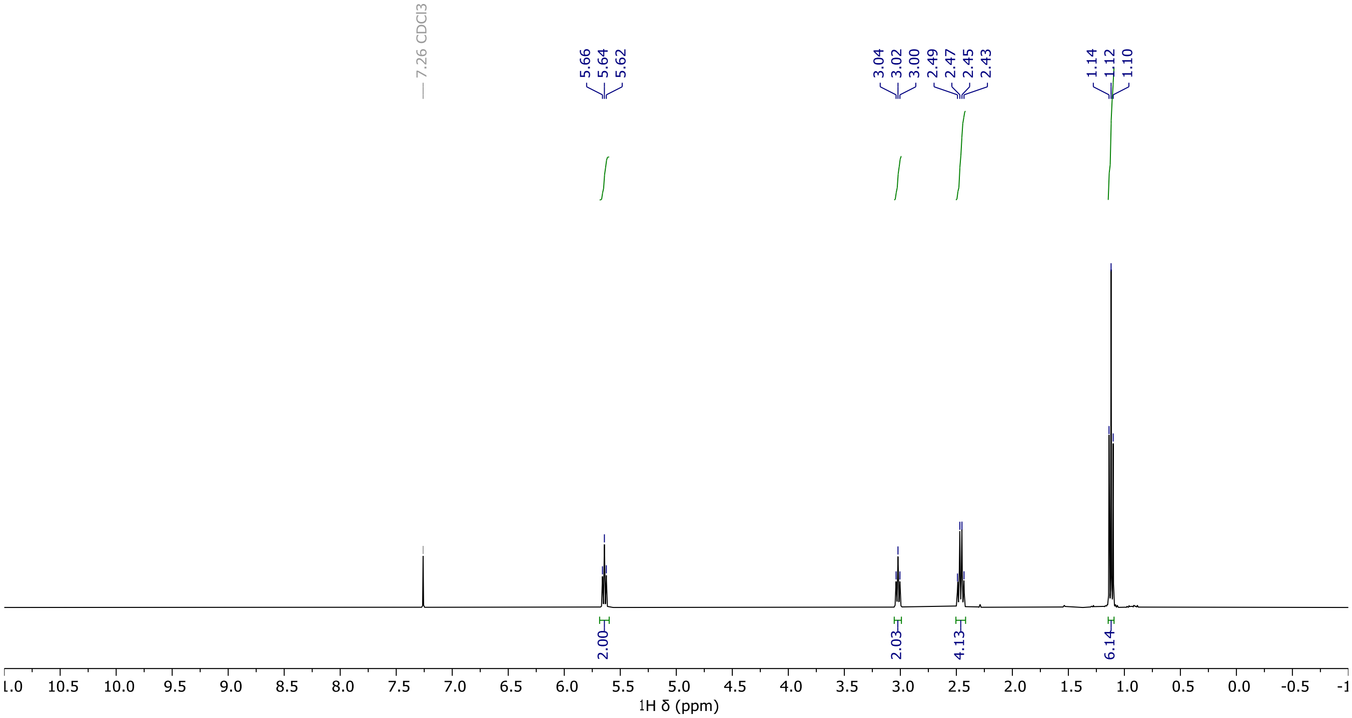

^{13}C{^{1}H} NMR of (3*Z*,6*Z*)-3,7-dibromonona-3,6-diene (**3c**) in $CDCl_3$, 101 MHz

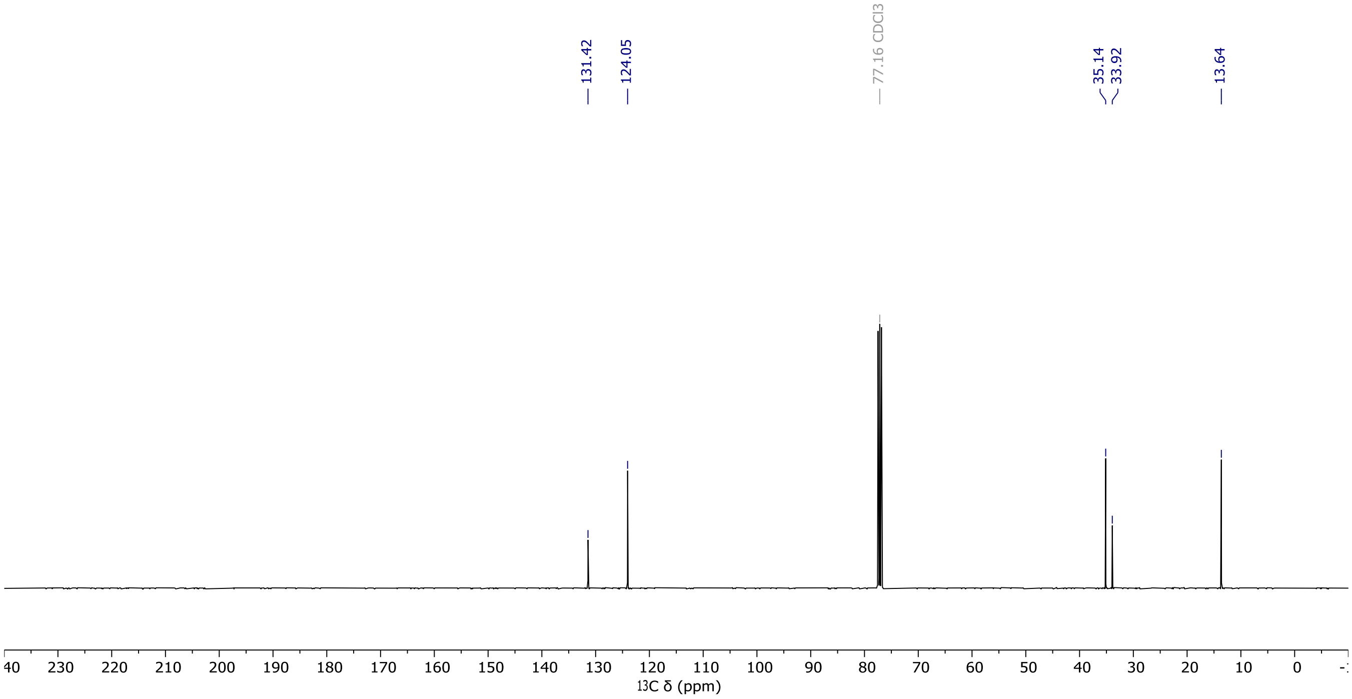

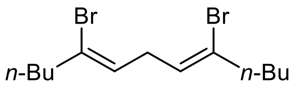

^{1}H NMR of (5*Z*,8*Z*)-5,9-dibromotrideca-5,8-diene (**3d**) in $CDCl_3$, 400 MHz

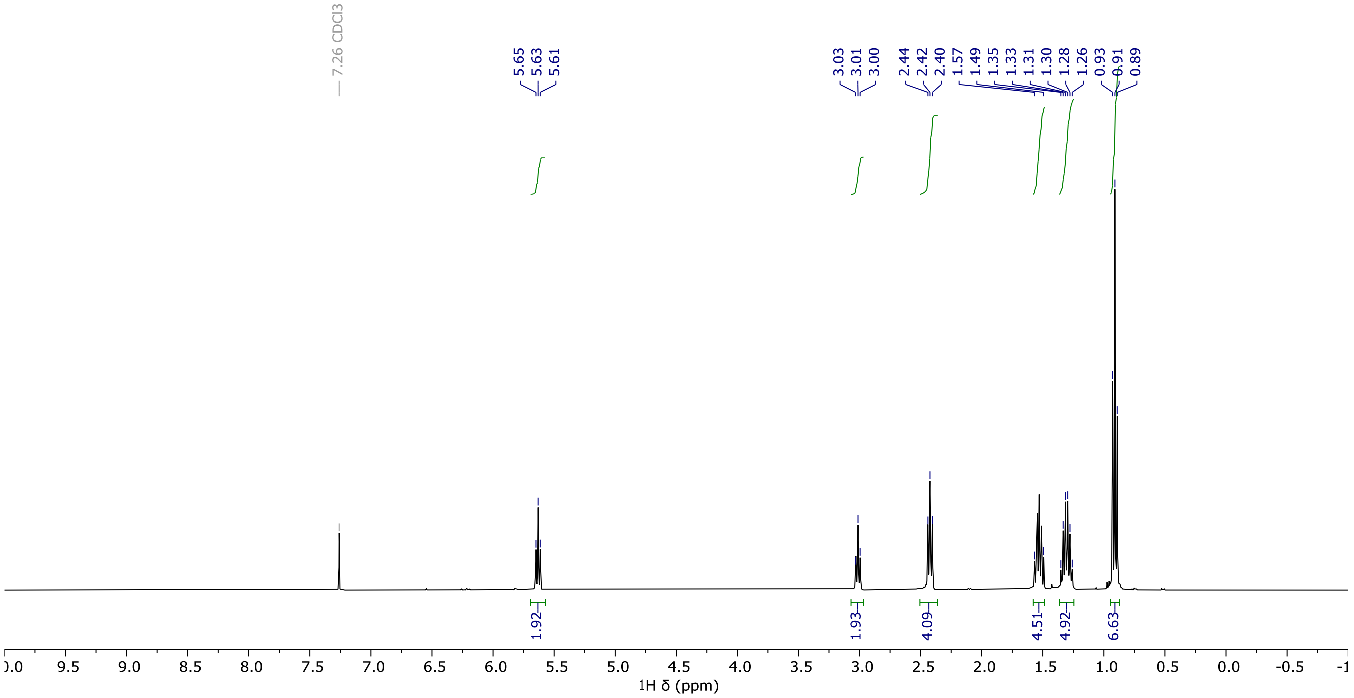

^{13}C{^{1}H} NMR of (5*Z*,8*Z*)-5,9-dibromotrideca-5,8-diene (**3d**) in $CDCl_3$, 101 MHz

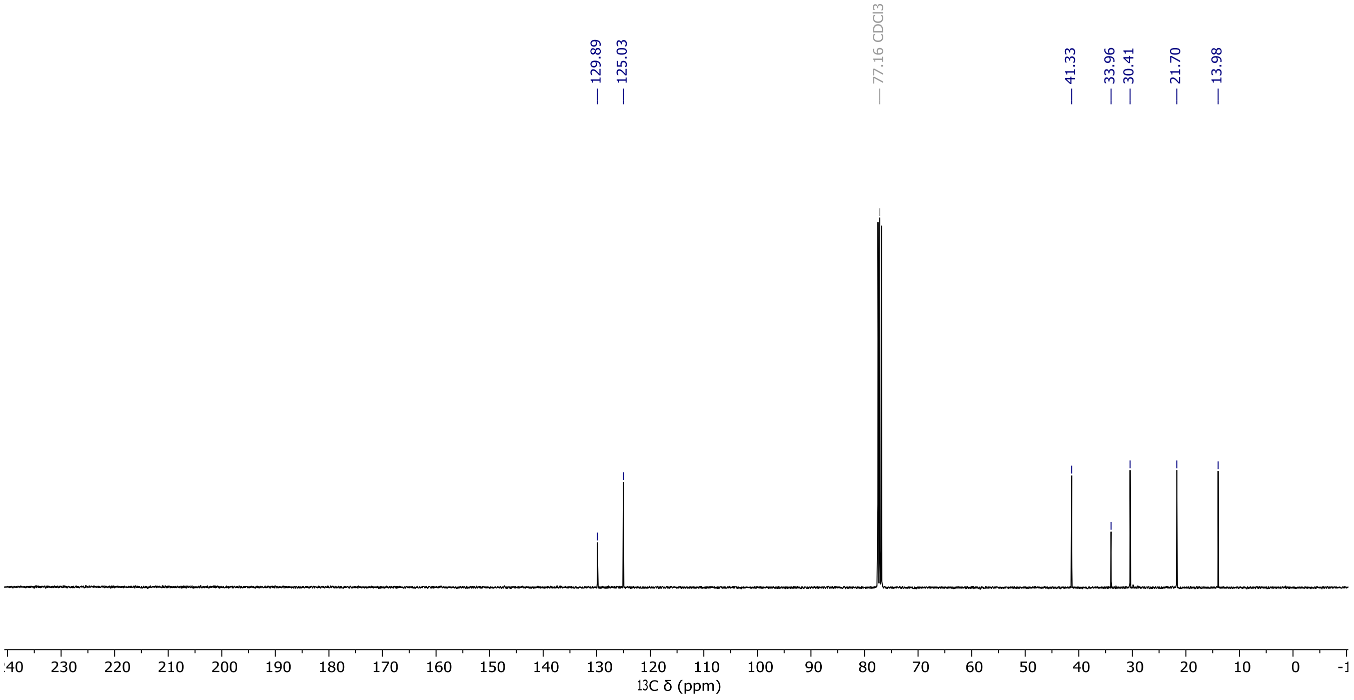

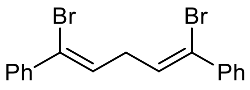

^{1}H NMR of (1*Z*,4*Z*)-1,5-dibromo-1,5-diphenylpenta-1,4-diene (**3e**) in $CDCl_3$, 400 MHz

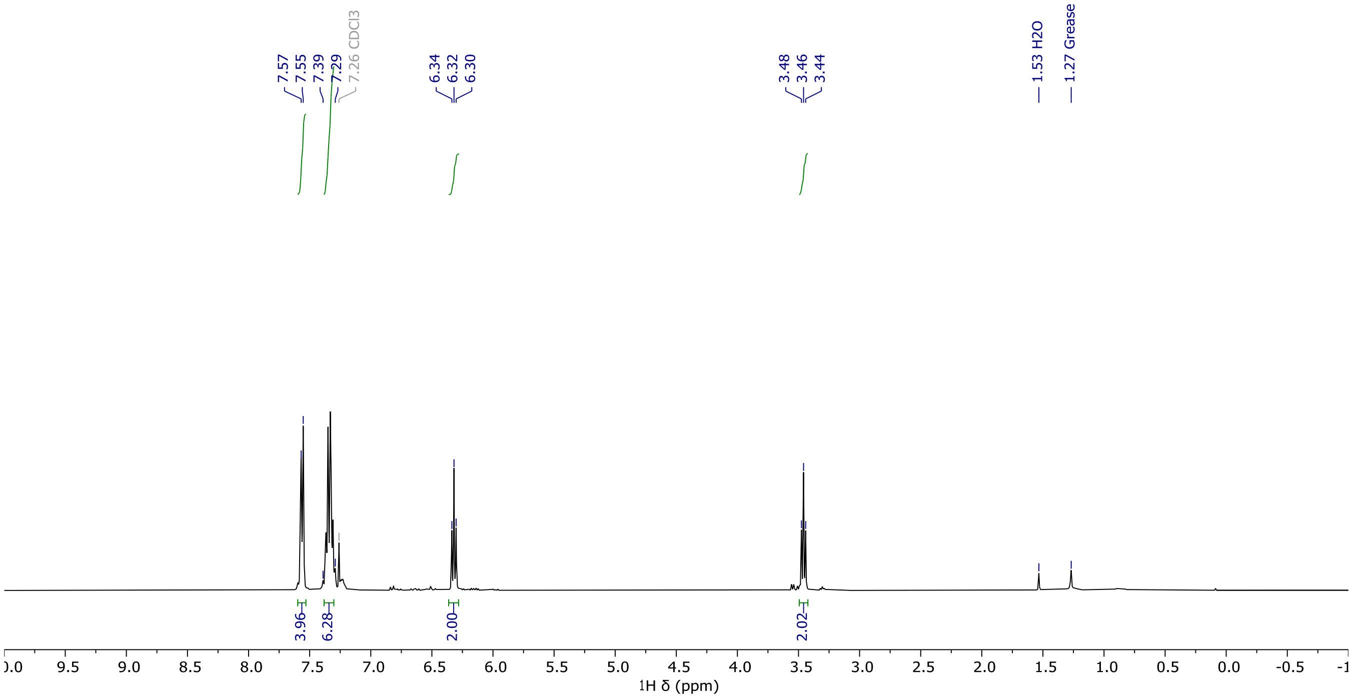

^{13}C{^{1}H} NMR of (1*Z*,4*Z*)-1,5-dibromo-1,5-diphenylpenta-1,4-diene (**3e**) in $CDCl_3$, 400 MHz

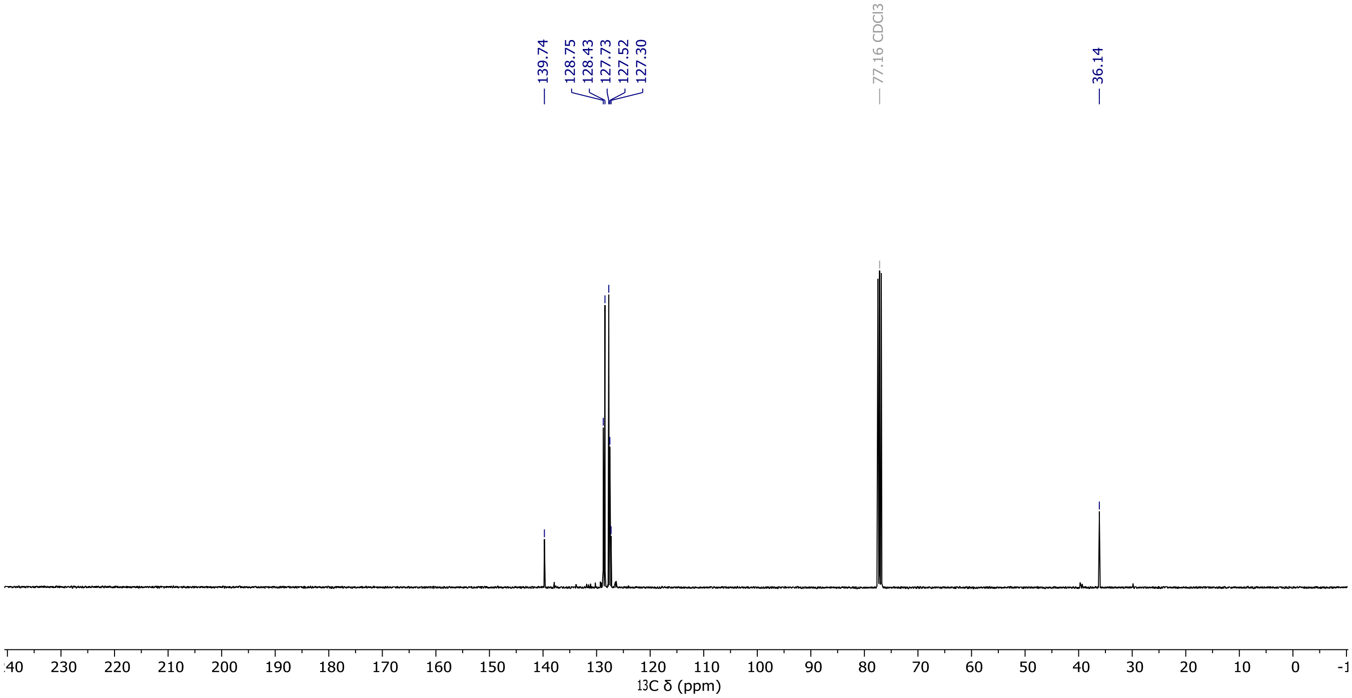

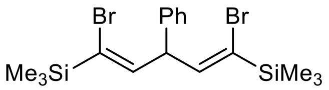

^{1}H NMR of ((1*Z*,4*Z*)-1,5-dibromo-3-phenylpenta-1,4-diene-1,5-diyl)bis(trimethylsilane) (**3f-TMS**) in $CDCl_3$, 400 MHz

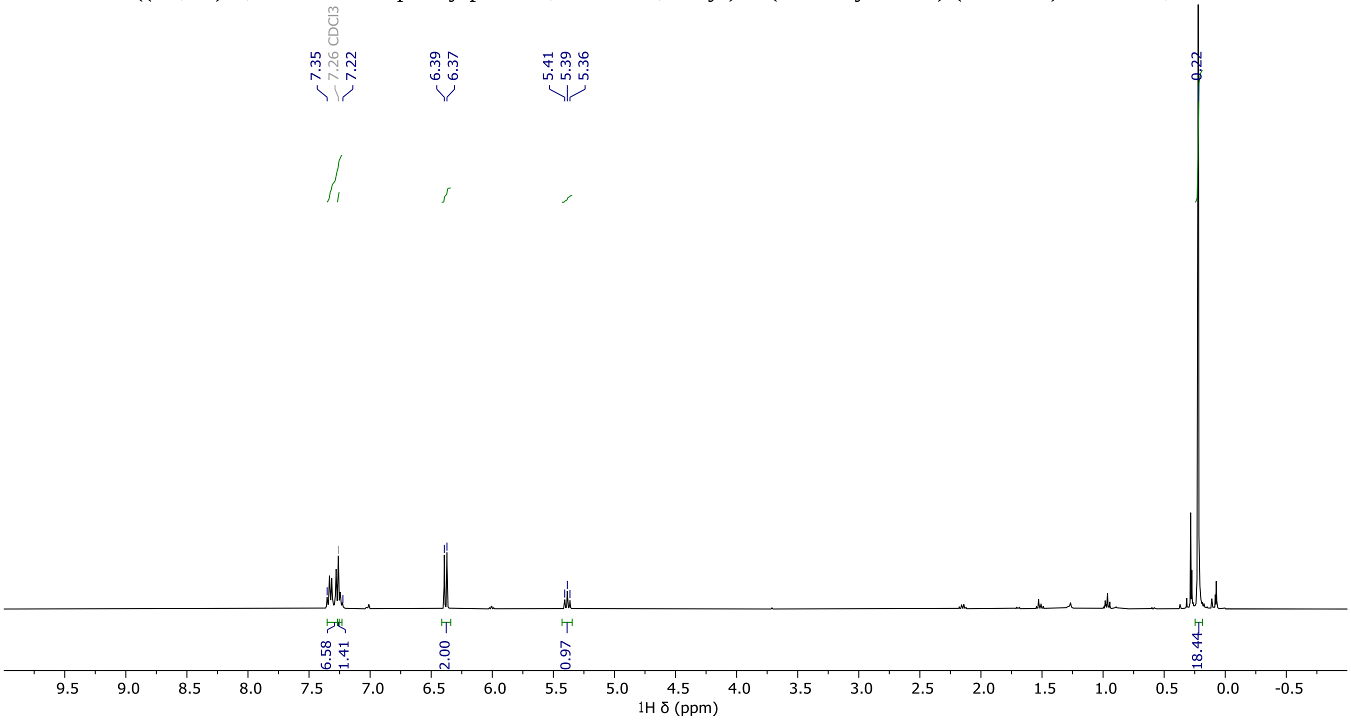

^{13}C{^{1}H} NMR of ((1*Z*,4*Z*)-1,5-dibromo-3-phenylpenta-1,4-diene-1,5-diyl)bis(trimethylsilane) (**3f-TMS**) in $CDCl_3$, 101 MHz

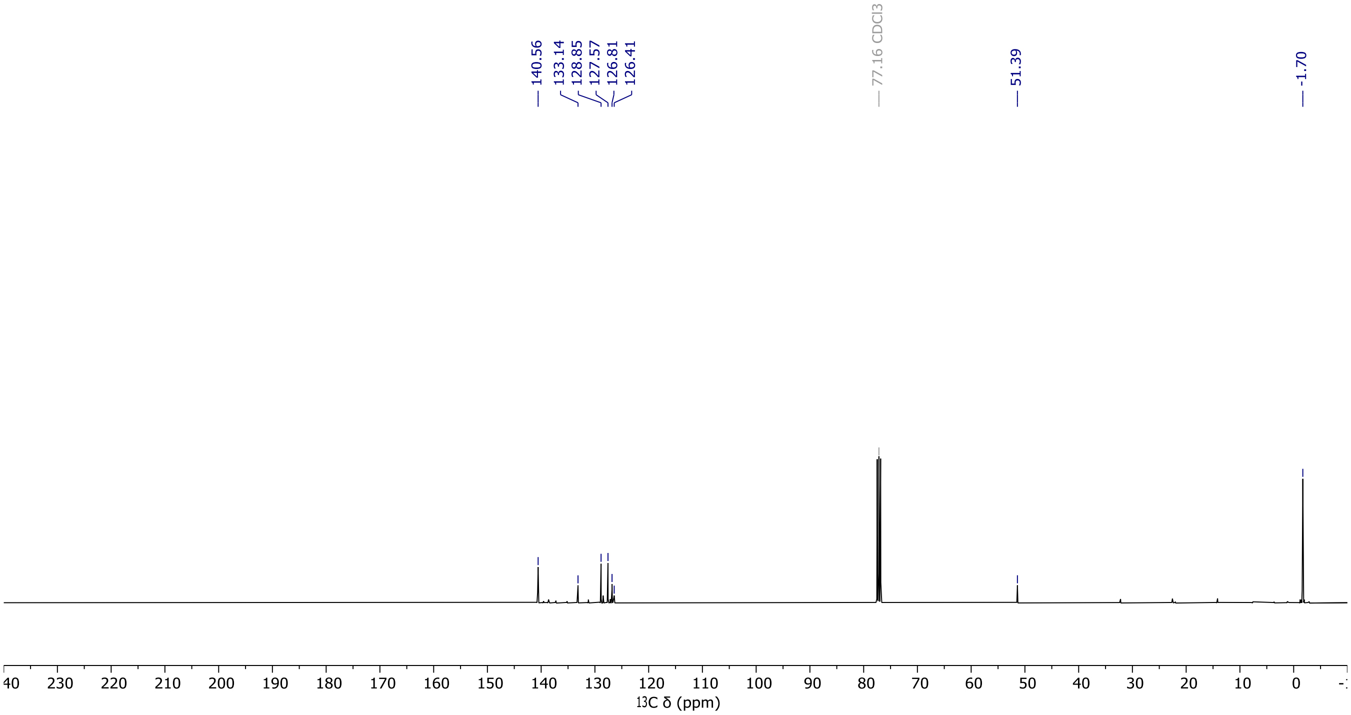

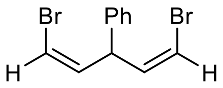

^{1}H NMR of ((1*Z*,4*Z*)-1,5-dibromopenta-1,4-dien-3-yl)benzene (**3f**) in $CDCl_3$, 400 MHz

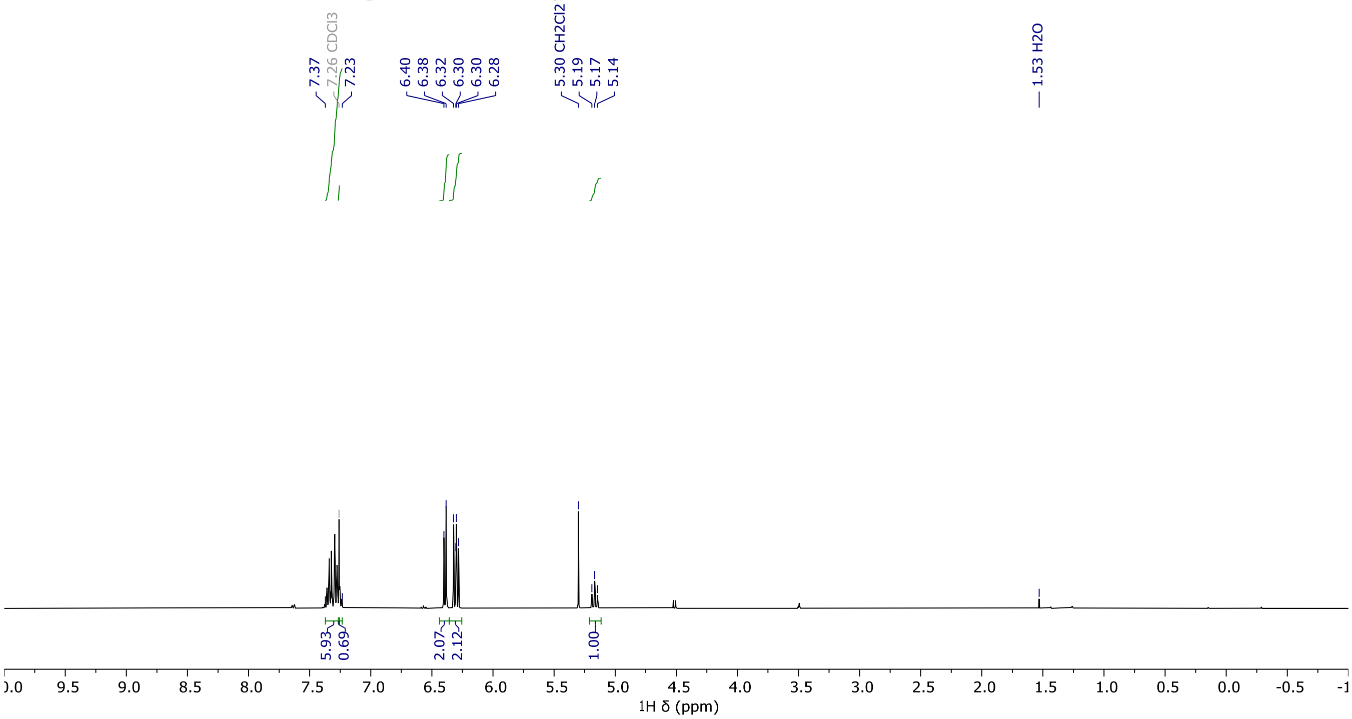

^{13}C{^{1}H} NMR of ((1*Z*,4*Z*)-1,5-dibromopenta-1,4-dien-3-yl)benzene (**3f**) in $CDCl_3$, 101 MHz

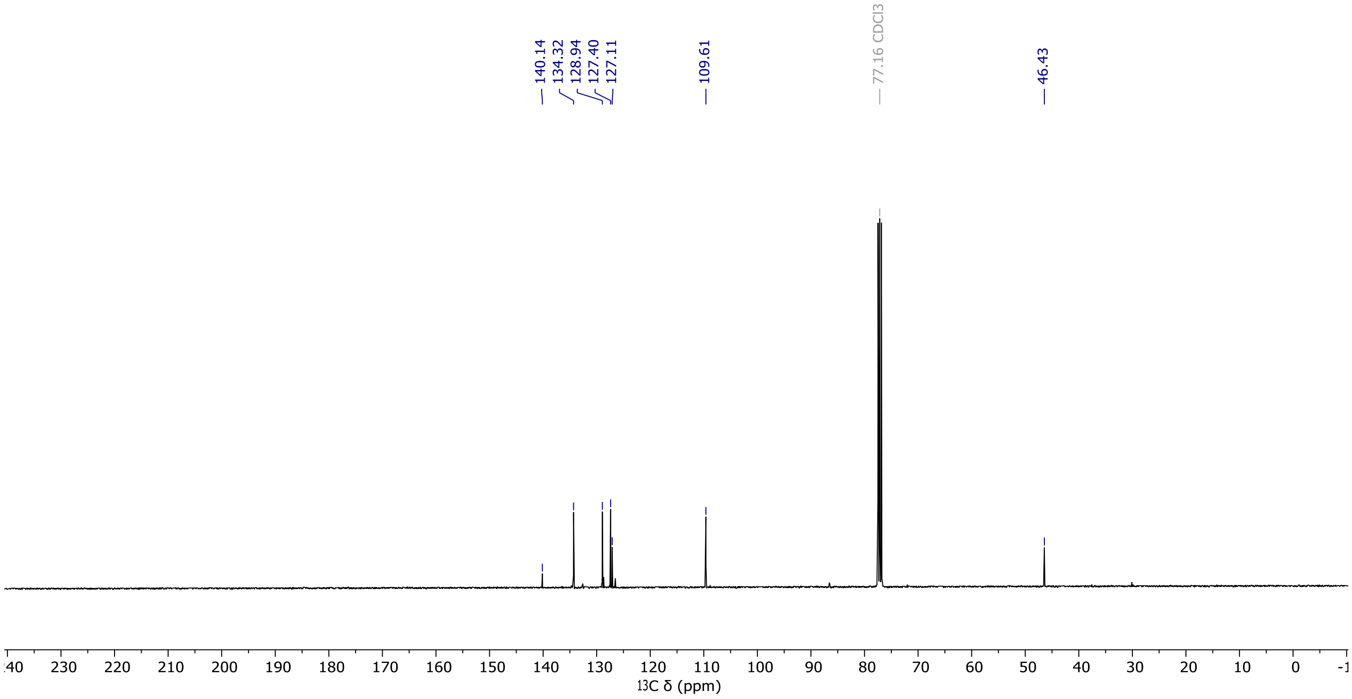

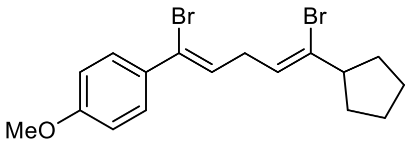

^{1}H NMR of 1-((1*Z*,4*Z*)-1,5-dibromo-5-cyclopentylpenta-1,4-dien-1-yl)-4-methoxybenzene (**3g**) in $CDCl_3$, 400 MHz

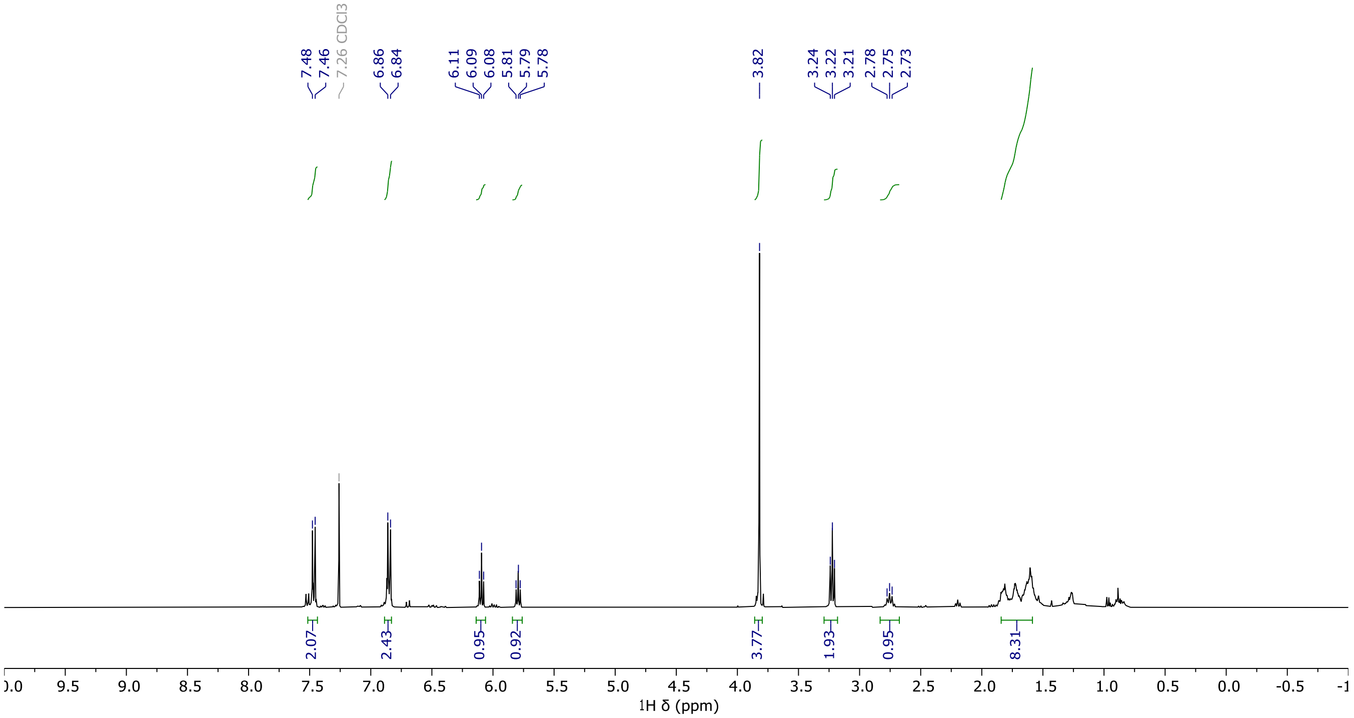

^{13}C{^{1}H} NMR of 1-((1*Z*,4*Z*)-1,5-dibromo-5-cyclopentylpenta-1,4-dien-1-yl)-4-methoxybenzene (**3g**) in $CDCl_3$, 101 MHz

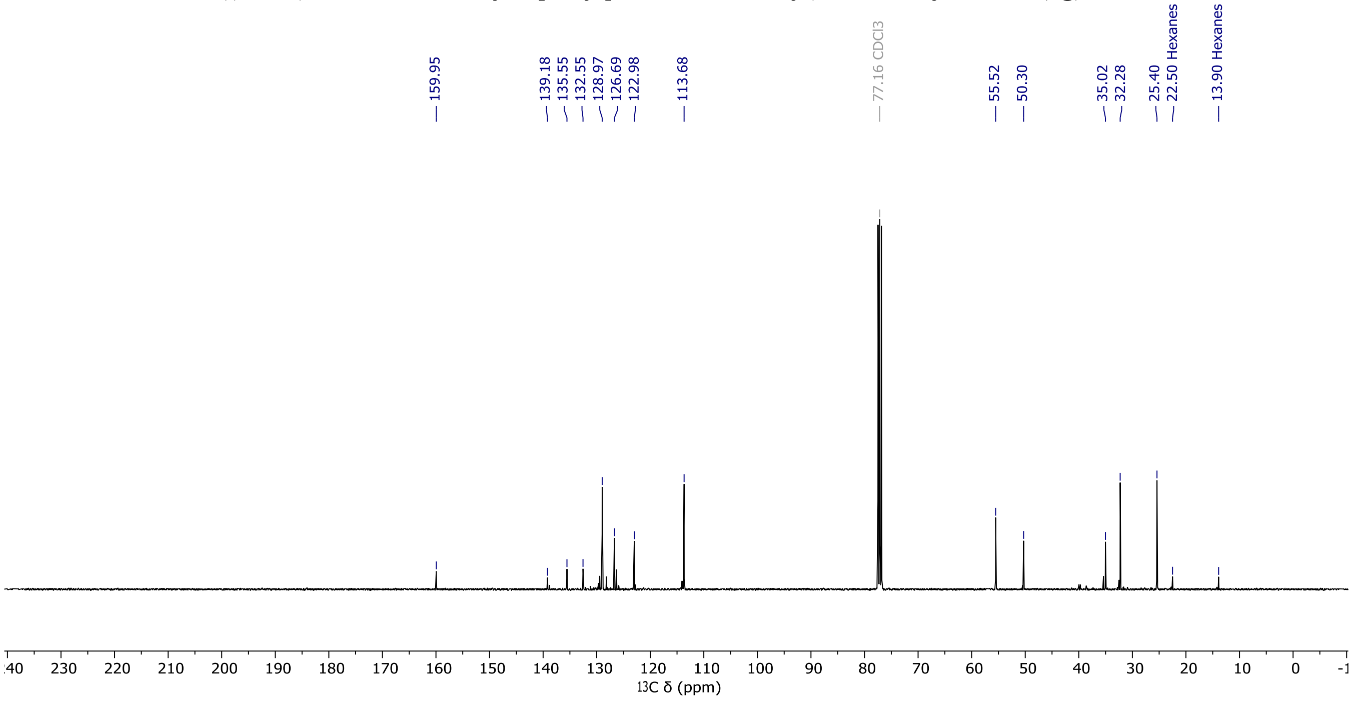

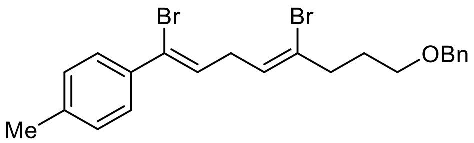

^{1}H NMR of 1-((1*Z*,4*Z*)-8-(benzyloxy)-1,5-dibromoocta-1,4-dien-1-yl)-4-methylbenzene (**3h**) in $CDCl_3$, 400 MHz

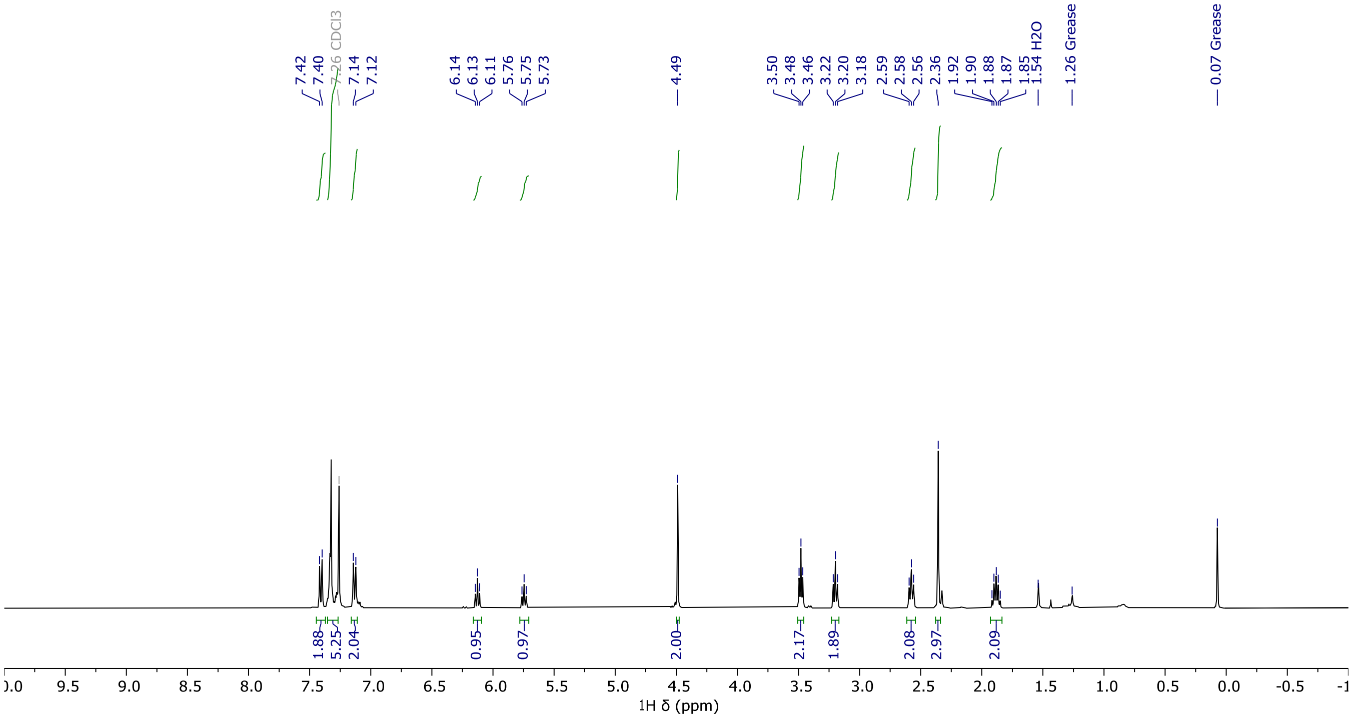

^{13}C{^{1}H} NMR of 1-((1*Z*,4*Z*)-8-(benzyloxy)-1,5-dibromoocta-1,4-dien-1-yl)-4-methylbenzene (**3h**) in $CDCl_3$, 101 MHz

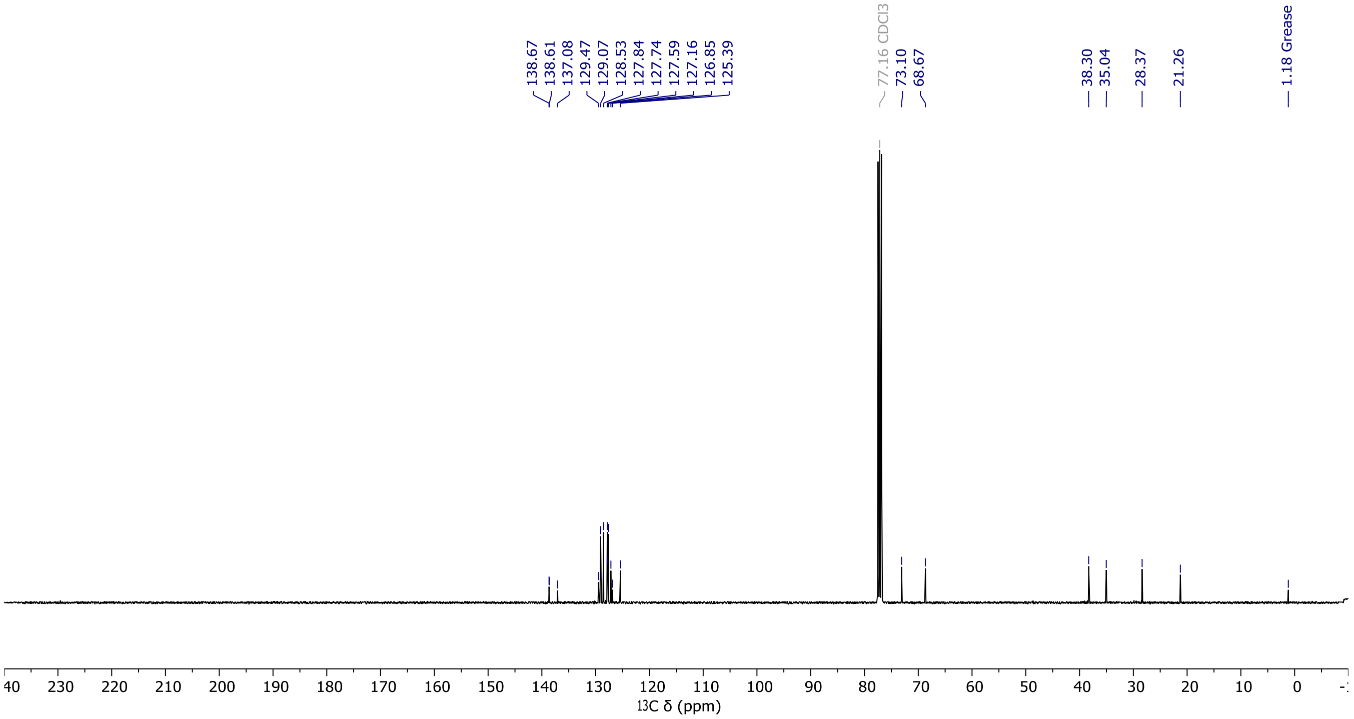

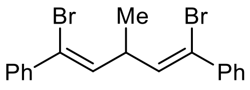

^{1}H NMR of ((1*Z*,4*Z*)-1,5-dibromo-3-methylpenta-1,4-diene-1,5-diyl)dibenzene (**3i**) in $CDCl_3$, 400 MHz

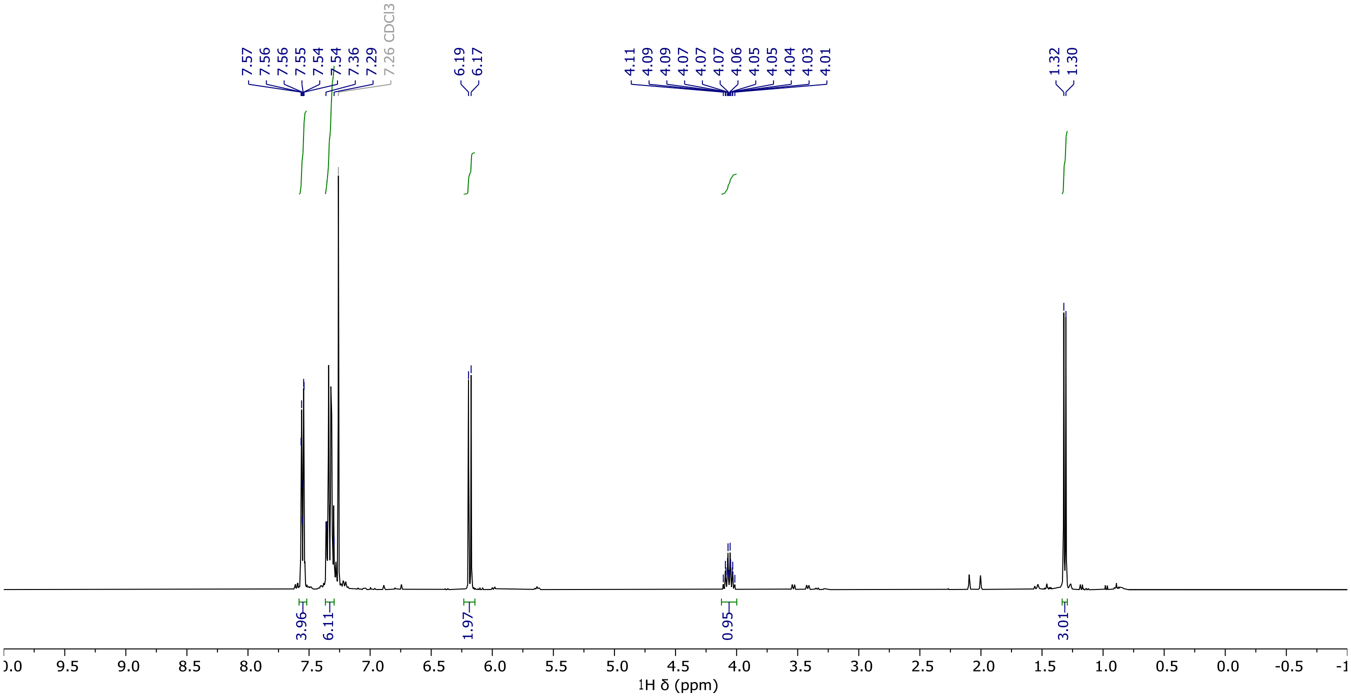

^{13}C{^{1}H} NMR of ((1*Z*,4*Z*)-1,5-dibromo-3-methylpenta-1,4-diene-1,5-diyl)dibenzene (**3i**) in $CDCl_3$, 101 MHz

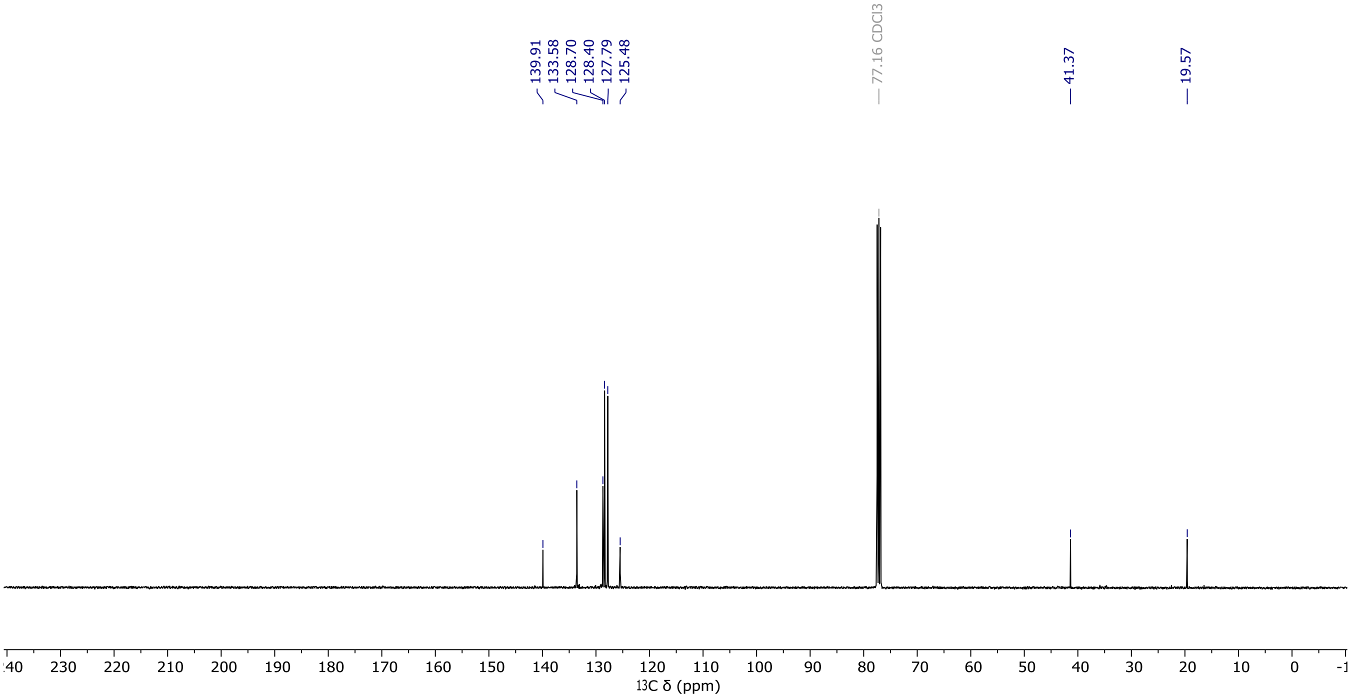

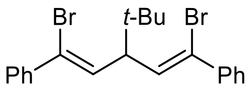

^{1}H NMR of ((1*Z*,4*Z*)-1,5-dibromo-3-(*tert*-butyl)penta-1,4-diene-1,5-diyl)dibenzene (**3k**) in $CDCl_3$, 400 MHz

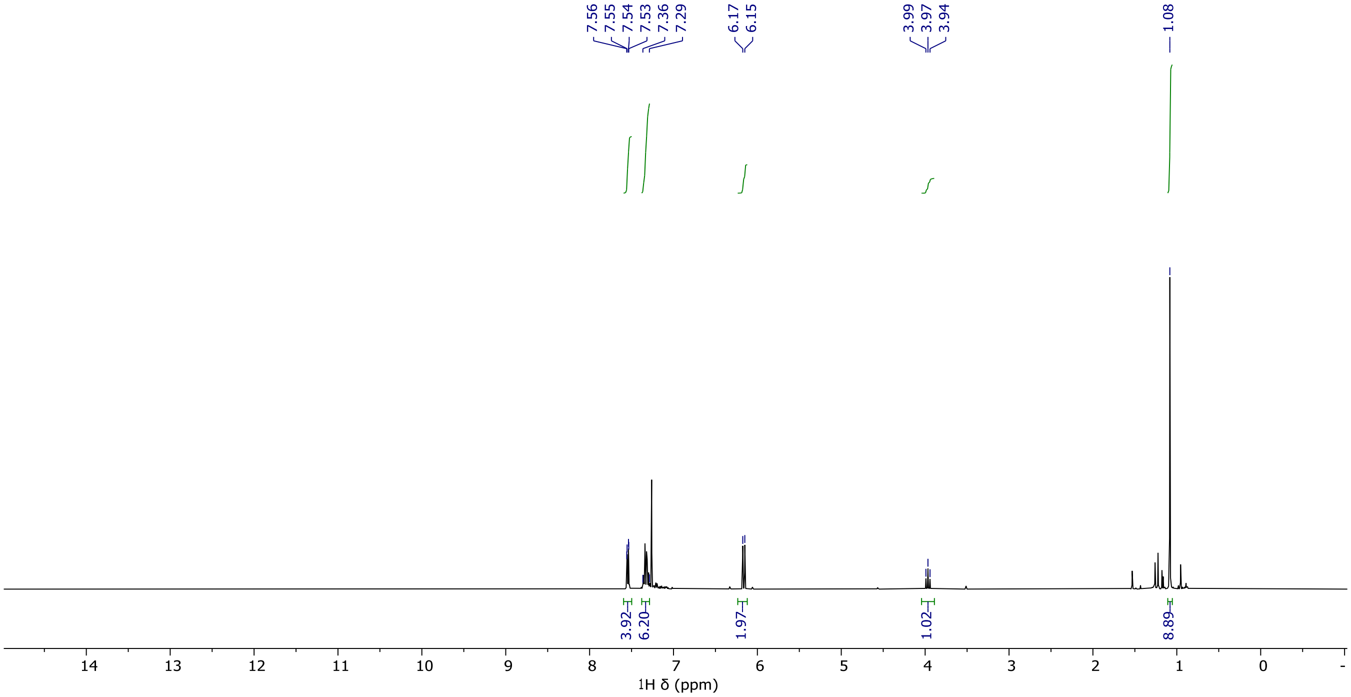

^{13}C{^{1}H} NMR of ((1*Z*,4*Z*)-1,5-dibromo-3-(*tert*-butyl)penta-1,4-diene-1,5-diyl)dibenzene (**3k**) in $CDCl_3$, 101 MHz

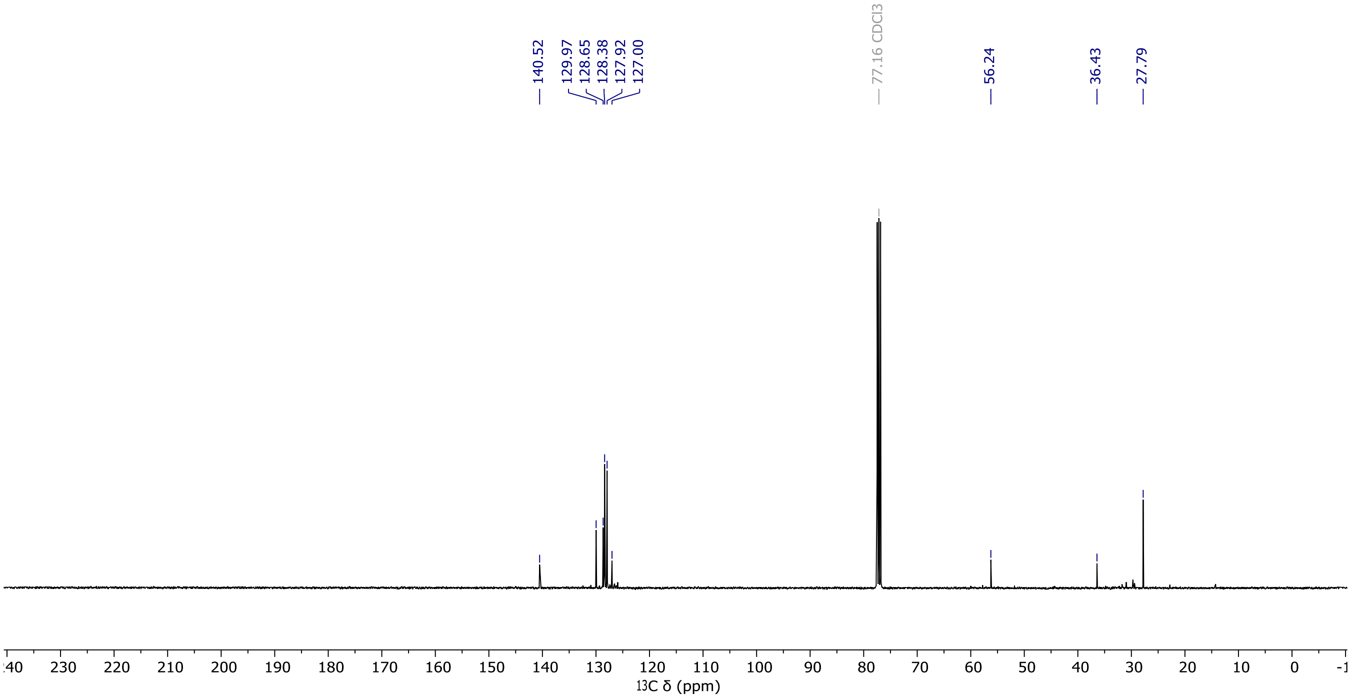

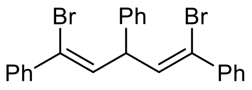

^{1}H NMR of ((1*Z*,4*Z*)-1,5-dibromopenta-1,4-diene-1,3,5-triyl)tribenzene (**3l**) in $CDCl_3$, 400 MHz

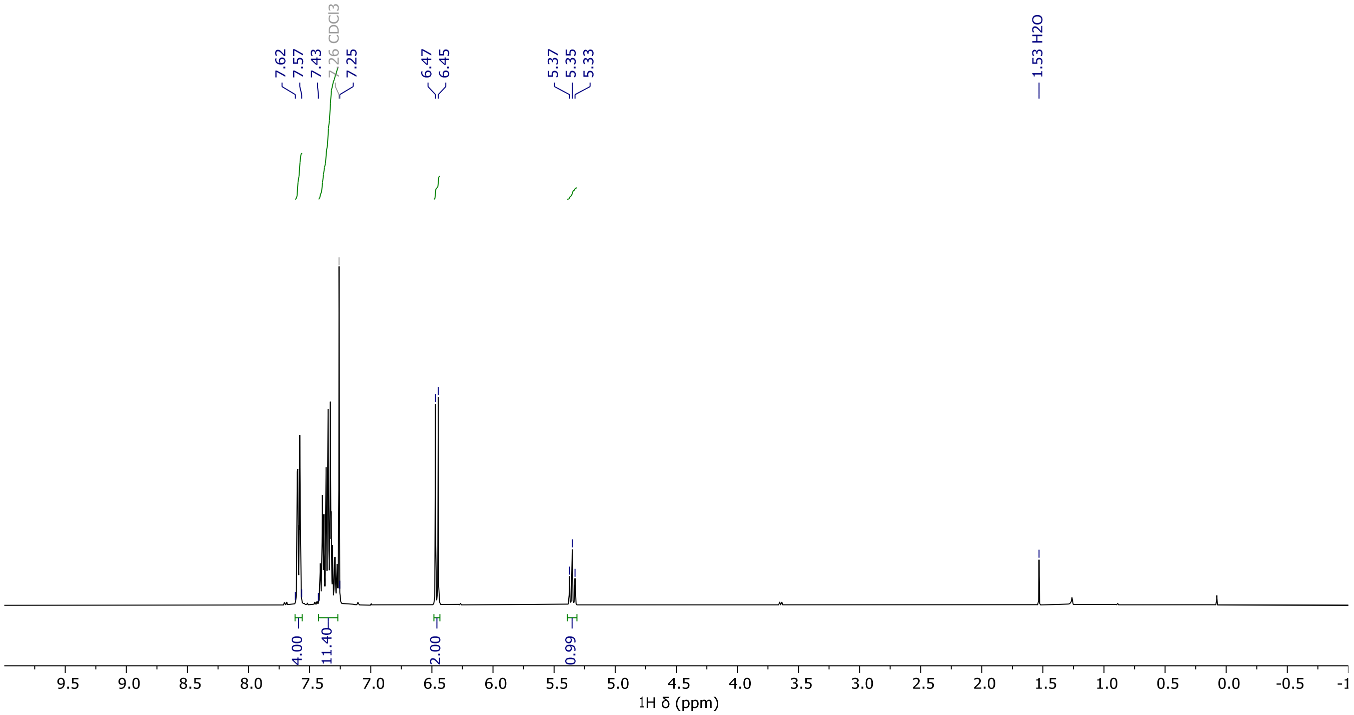

^{13}C{^{1}H} NMR of ((1*Z*,4*Z*)-1,5-dibromopenta-1,4-diene-1,3,5-triyl)tribenzene (**3l**) in $CDCl_3$, 101 MHz

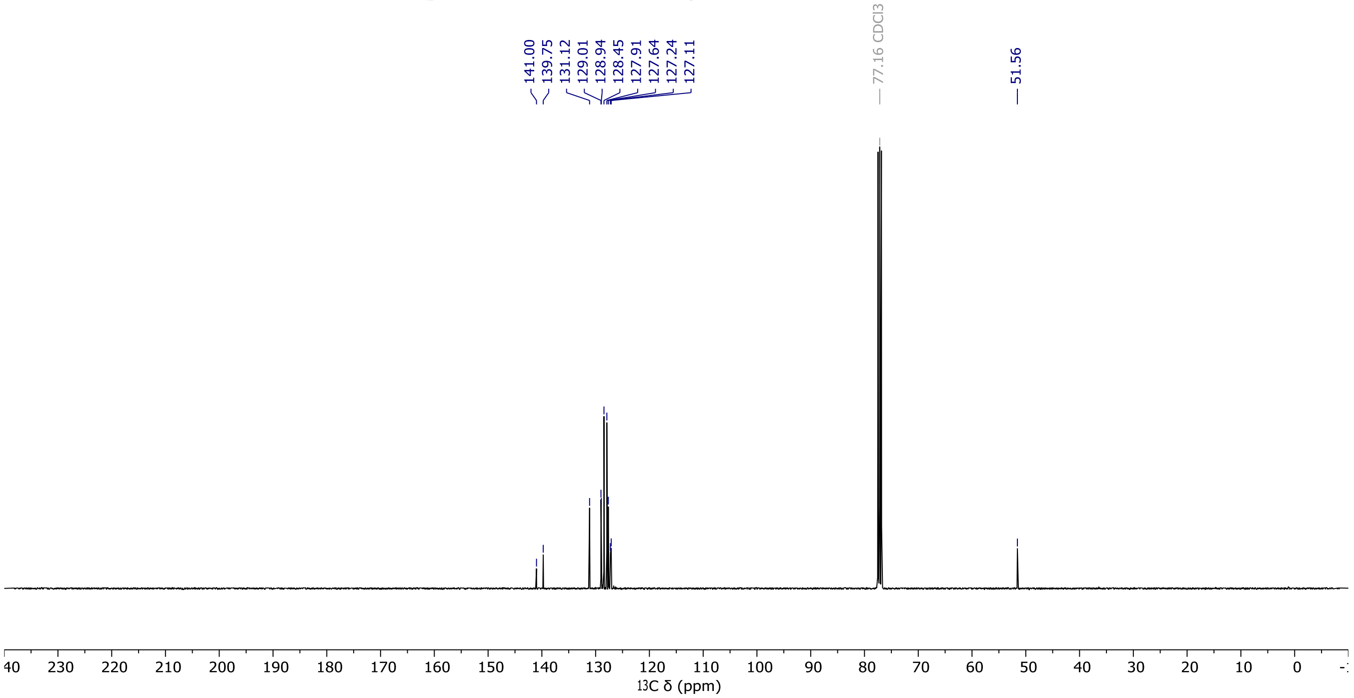

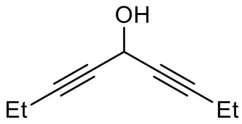

^{1}H NMR of nona-3,6-diyn-5-ol (**1c**) in $CDCl_3$, 400 MHz

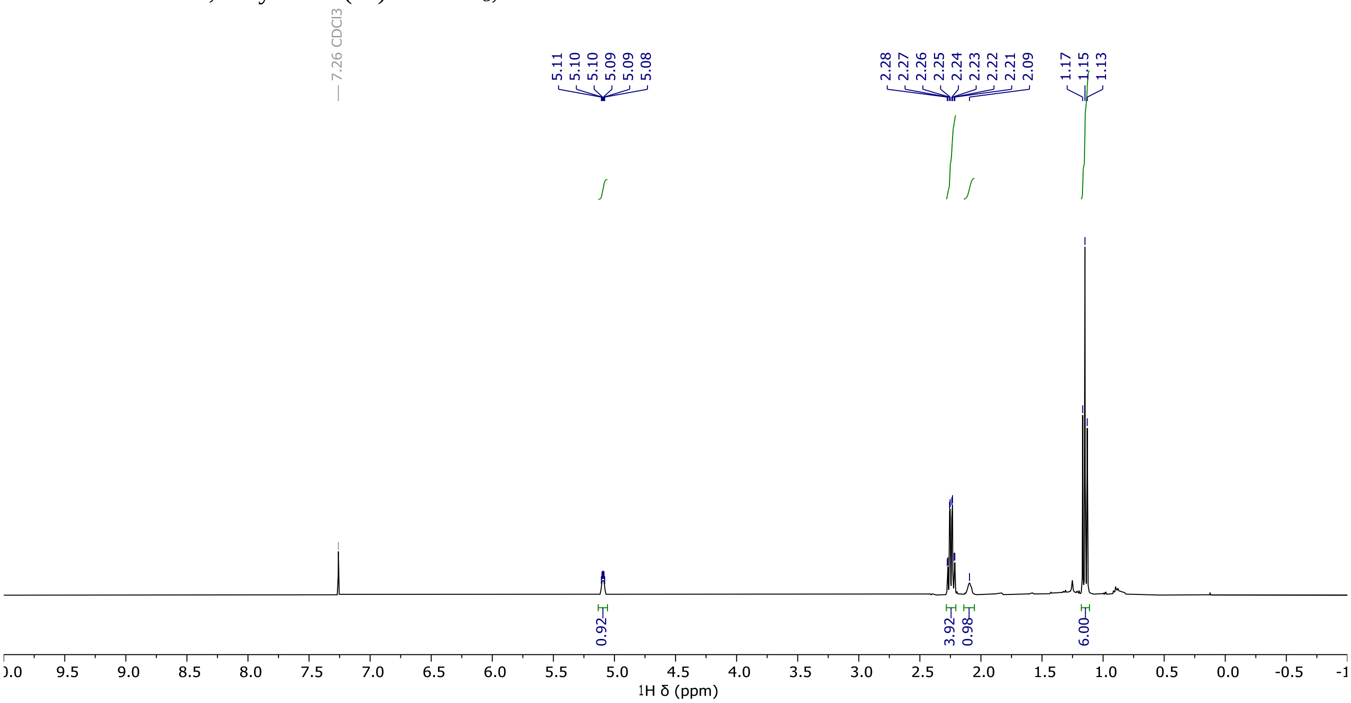

^{13}C{^{1}H} NMR of nona-3,6-diyn-5-ol (**1c**) in $CDCl_3$, 101 MHz

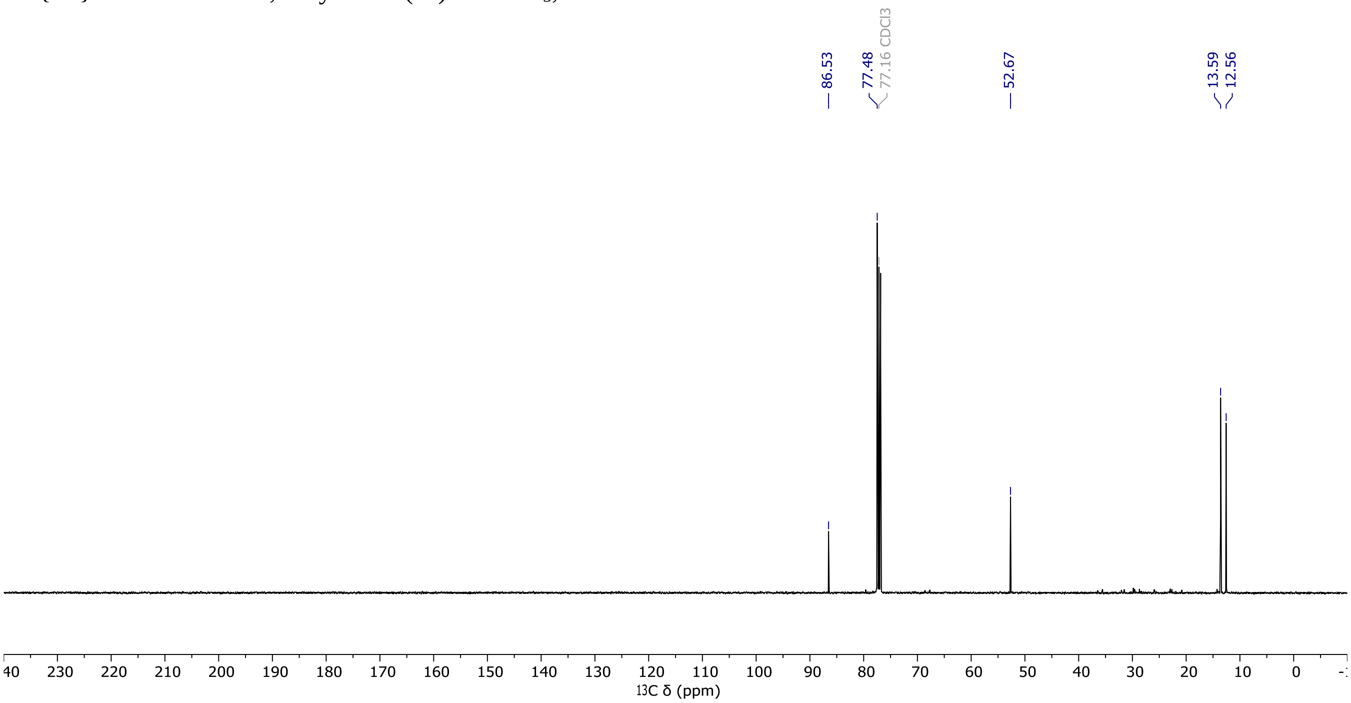

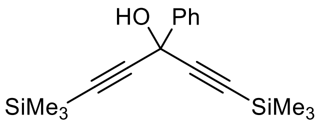

^{1}H NMR of 3-phenyl-1,5-bis(trimethylsilyl)penta-1,4-diyn-3-ol (**1f-TMS**) in $CDCl_3$, 400 MHz

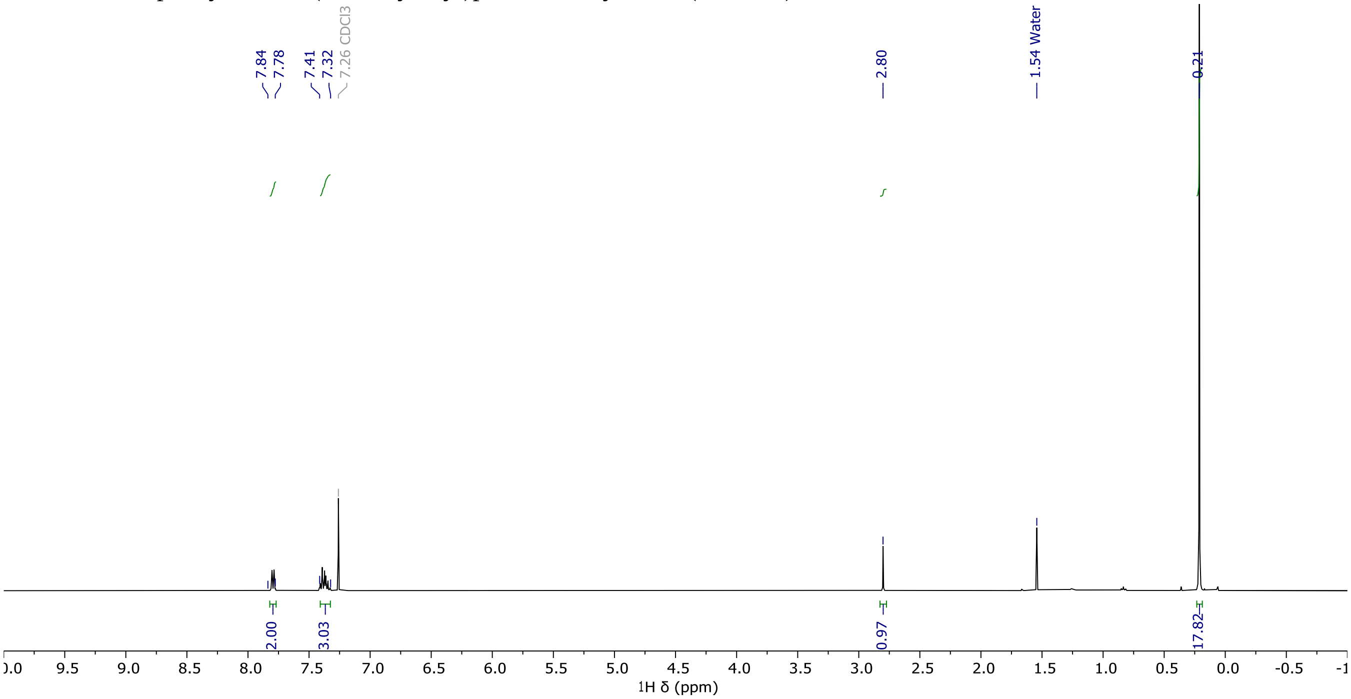

$^{13}C\{^{1}H\}$ NMR of 3-phenyl-1,5-bis(trimethylsilyl)penta-1,4-diyn-3-ol (**1f-TMS**) in $CDCl_3$, 101 MHz

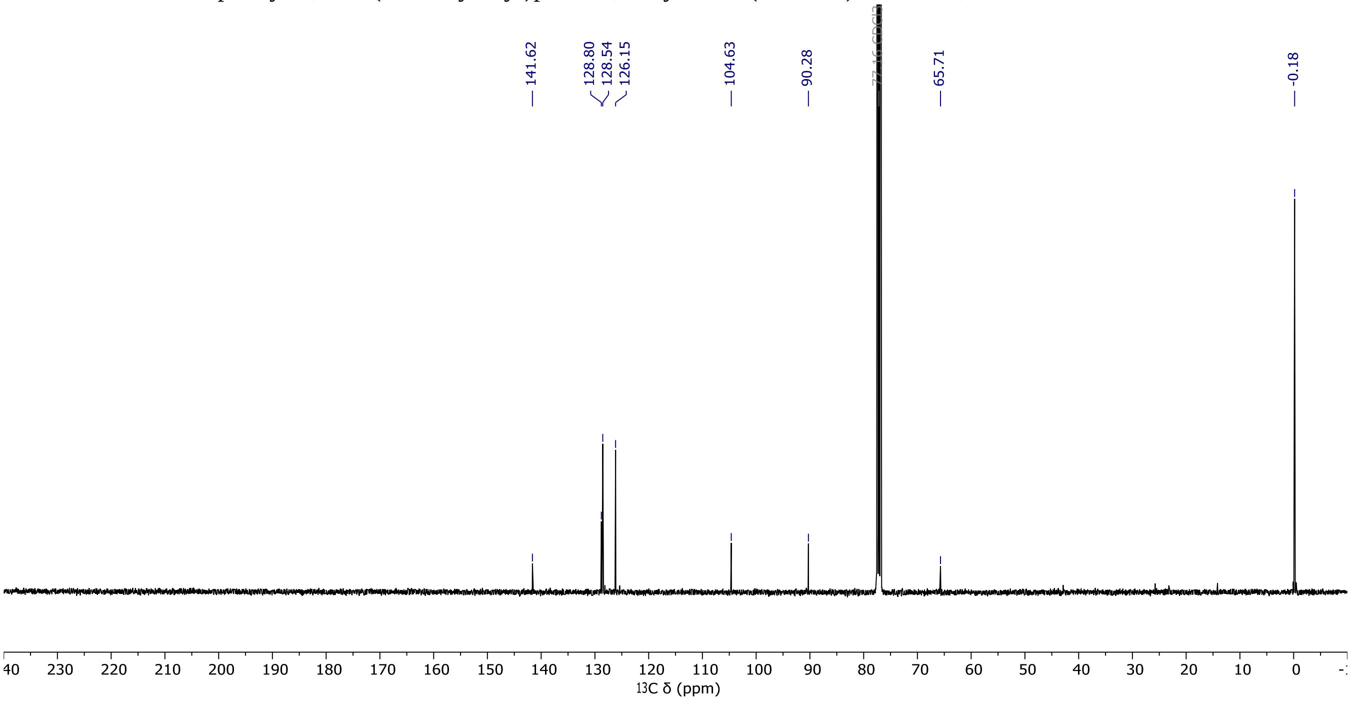

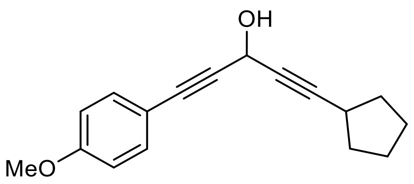

^{1}H NMR of 1-cyclopentyl-5-(4-methoxyphenyl)penta-1,4-diyn-3-ol (**1g**) in $CDCl_3$, 400 MHz

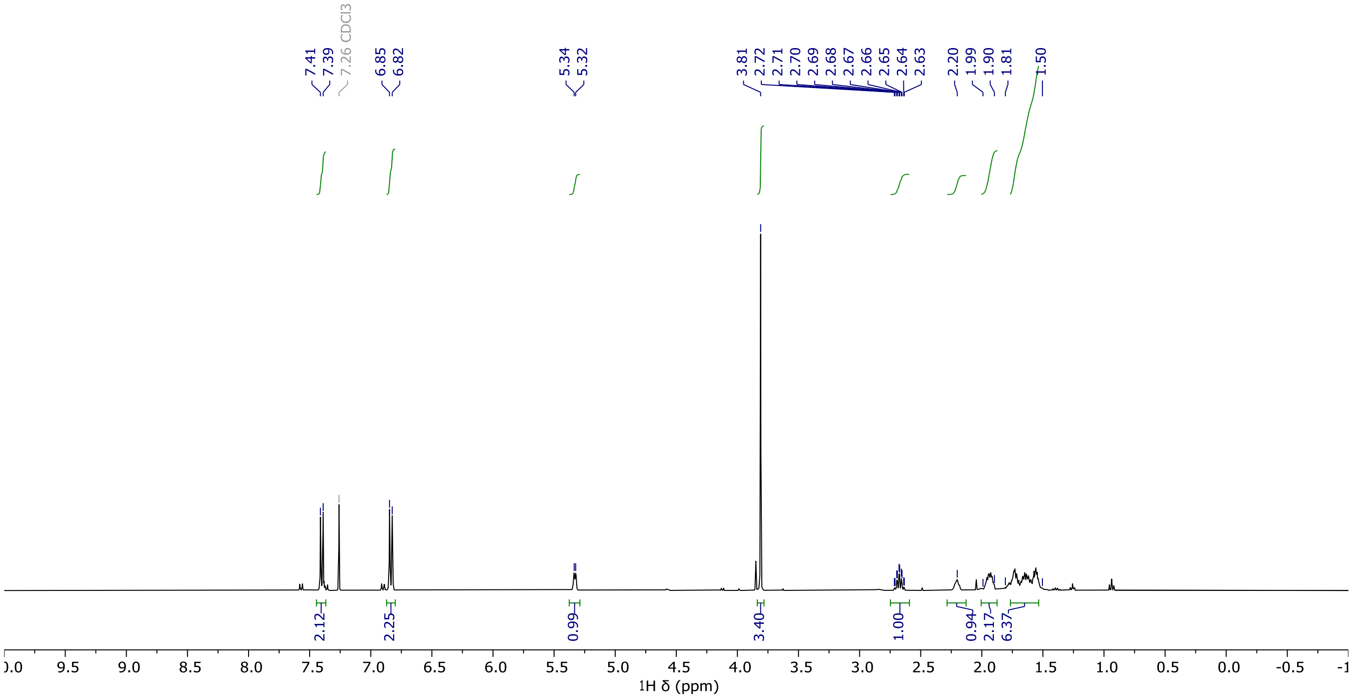

$^{13}C\{^1H\}$ NMR of 1-cyclopentyl-5-(4-methoxyphenyl)penta-1,4-diyn-3-ol (**1g**) in $CDCl_3$, 101 MHz

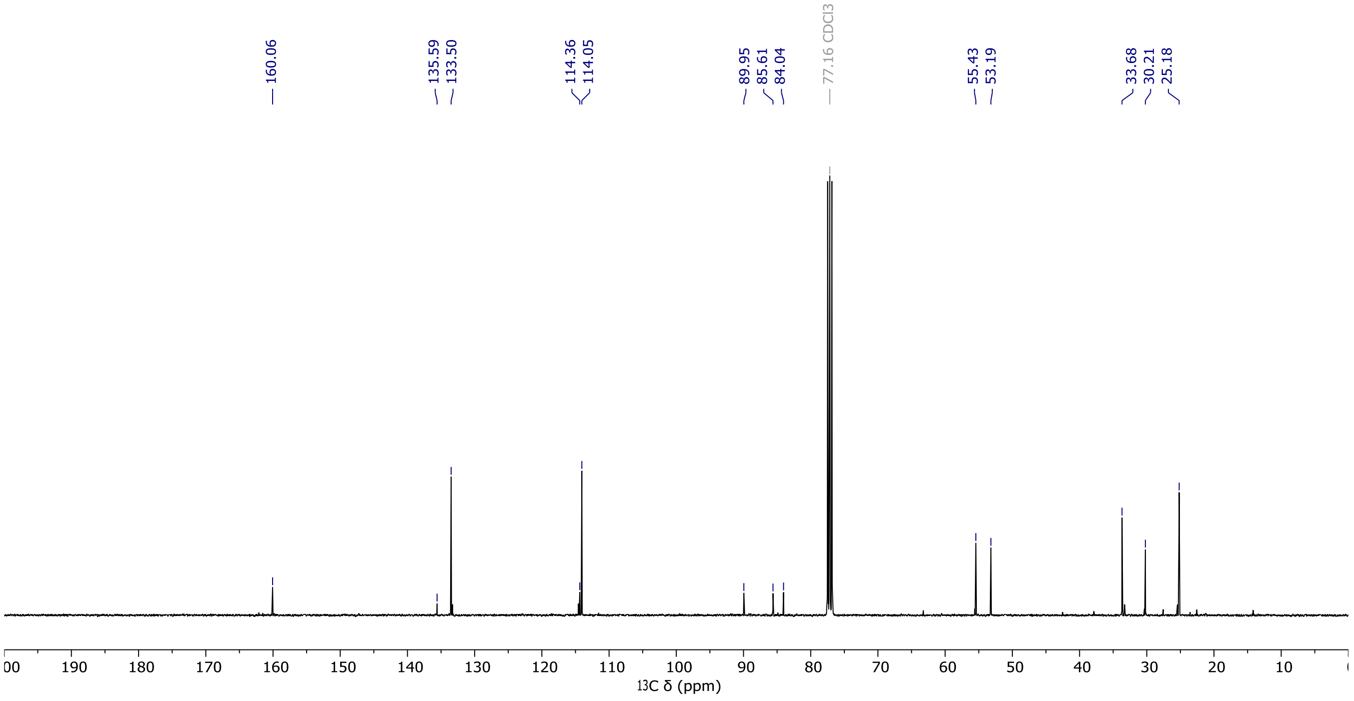

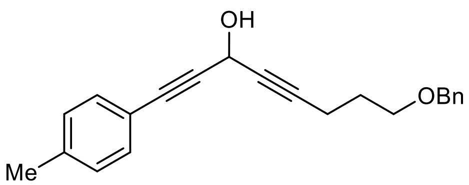

^{1}H NMR of 8-(benzyloxy)-1-(*p*-tolyl)octa-1,4-diyn-3-ol (**1h**) in $CDCl_3$, 400 MHz

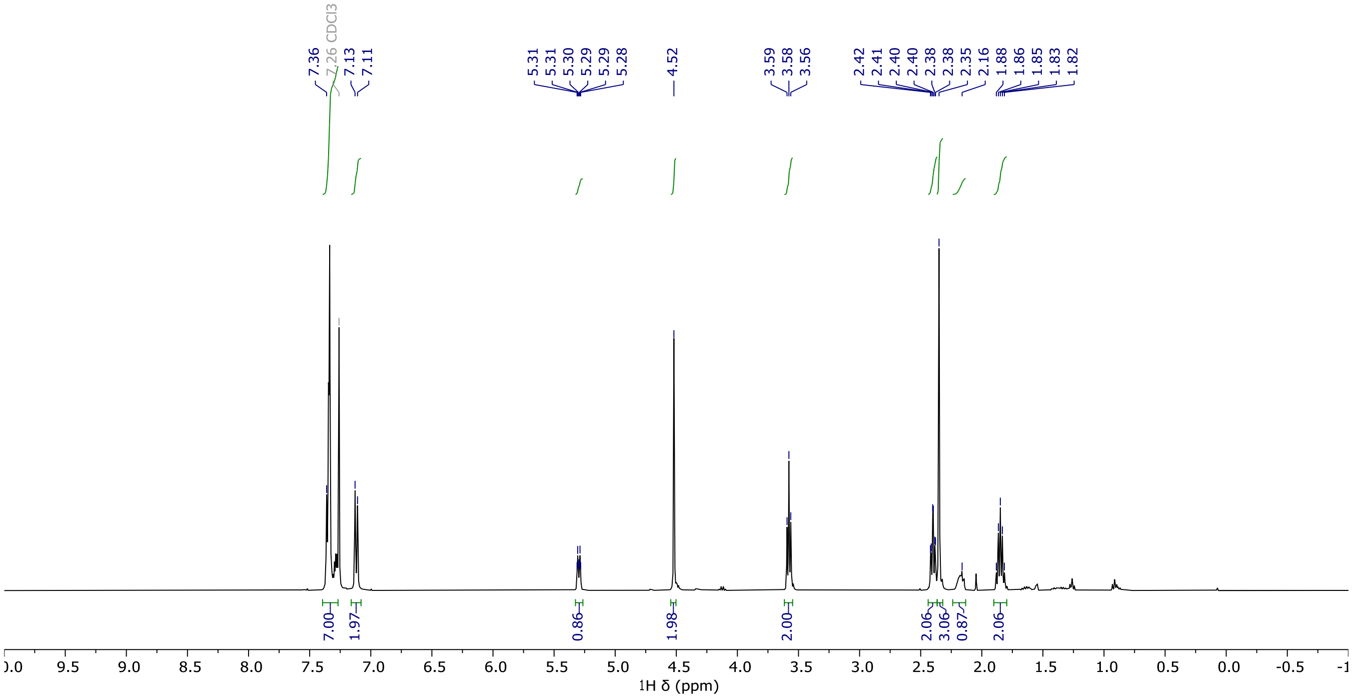

$^{13}C\{^{1}H\}$ NMR of 8-(benzyloxy)-1-(*p*-tolyl)octa-1,4-diyn-3-ol (**1h**) in $CDCl_3$, 101 MHz

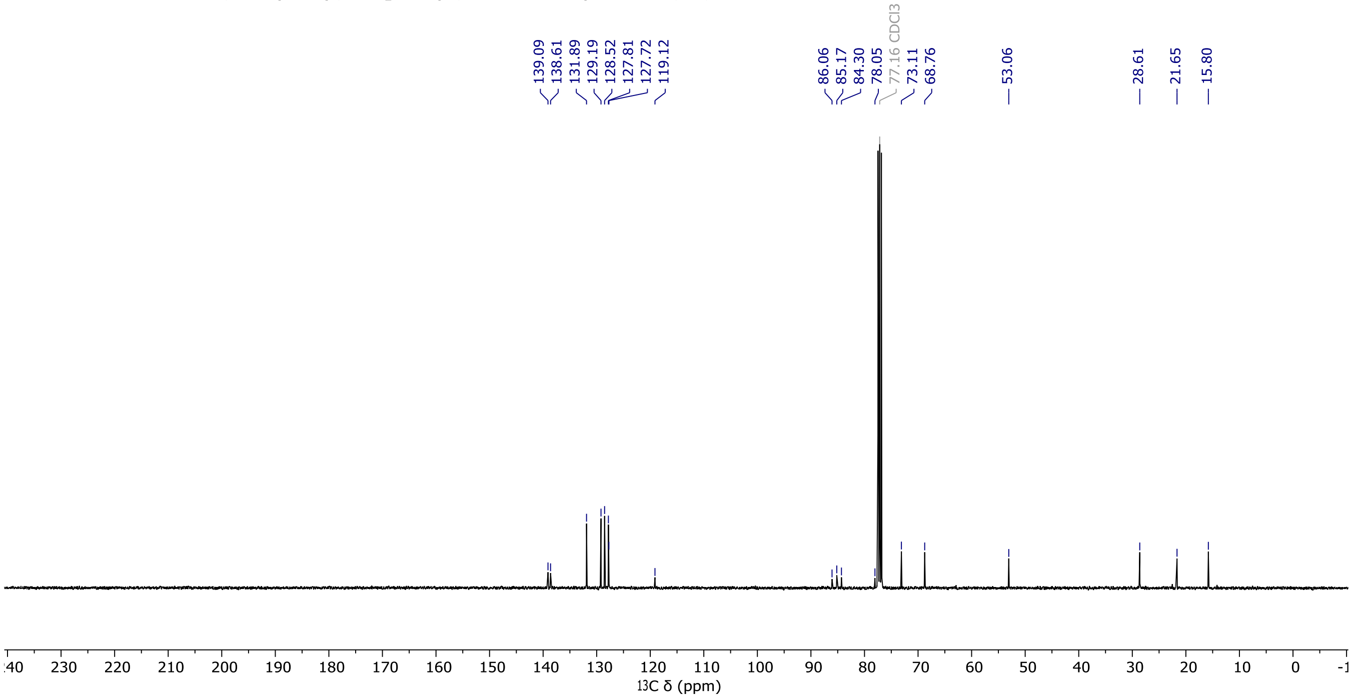

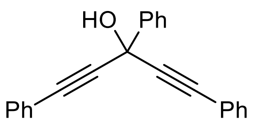

^{1}H NMR of 1,3,5-triphenylpenta-1,4-diyn-3-ol (**1l**) in $CDCl_3$, 400 MHz

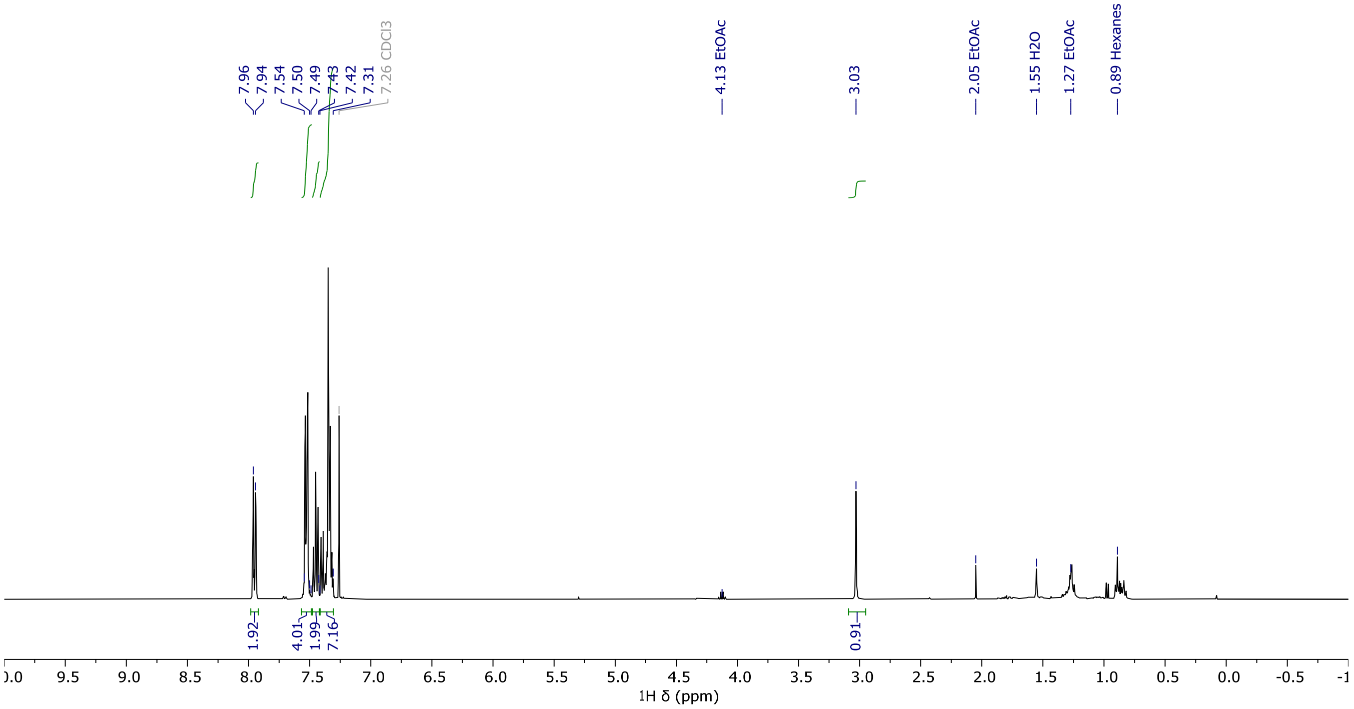

^{13}C{^{1}H} NMR of 1,3,5-triphenylpenta-1,4-diyn-3-ol (**1l**) in $CDCl_3$, 101 MHz

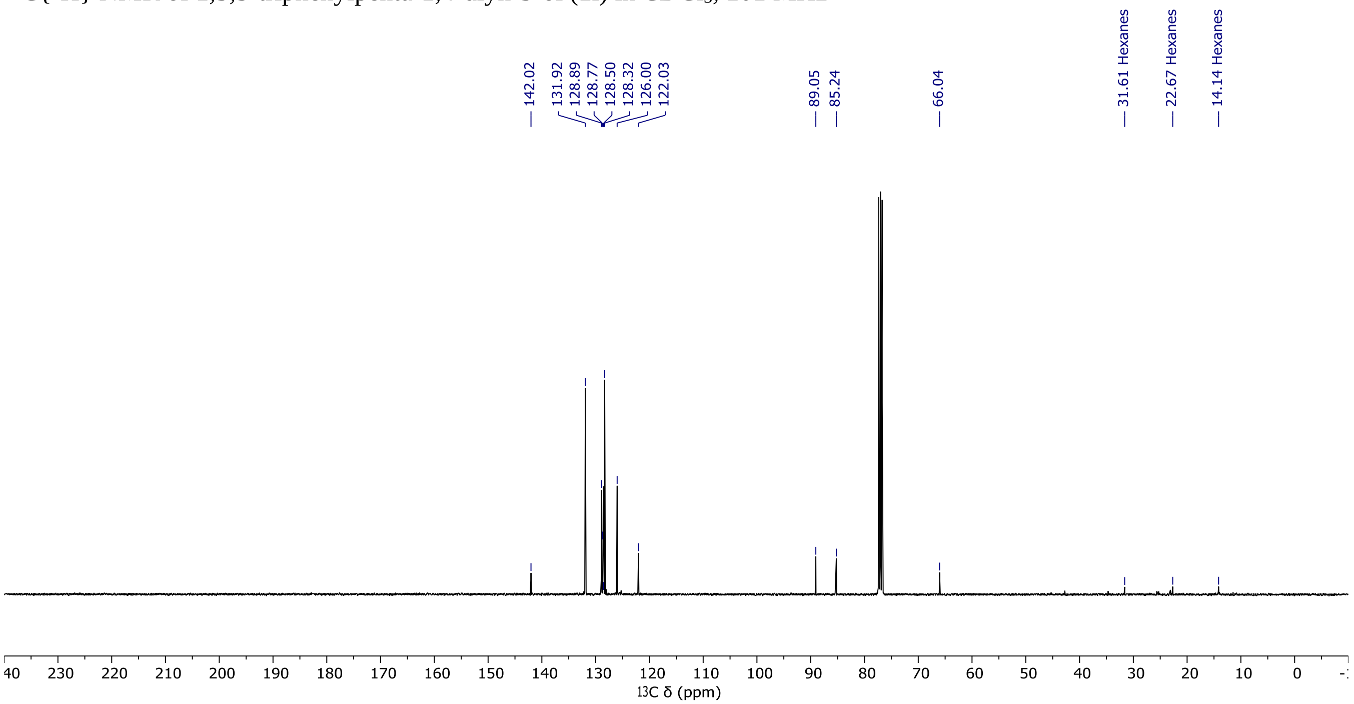

Supplement: Supplementary file 1 — ol3c02838_si_001.pdf [file ol3c02838_si_001.pdf]
